# Chromosome-level echidna genome illuminates evolution of multiple sex chromosome system in monotremes

**DOI:** 10.1093/gigascience/giae112

**Published:** 2025-01-09

**Authors:** Yang Zhou, Jiazheng Jin, Xuemei Li, Gregory Gedman, Sarah Pelan, Arang Rhie, Chuan Jiang, Olivier Fedrigo, Kerstin Howe, Adam M Phillippy, Erich D Jarvis, Frank Grutzner, Qi Zhou, Guojie Zhang

**Affiliations:** State Key Laboratory of Agricultural Genomics, BGI Research, Shenzhen 518083, China; BGI Research, Wuhan 430074, China; BGI Research, Hangzhou 310030, China; BGI Research, Wuhan 430074, China; College of Life Sciences, University of Chinese Academy of Sciences, Beijing 100049, China; Laboratory of Neurogenetics of Language, The Rockefeller University, New York, NY 10065, USA; Tree of Life, Wellcome Sanger Institute, Cambridge CB10 1SA, UK; Genome Informatics Section, Computational and Statistical Genomics Branch, National Human Genome Research Institute, National Institutes of Health, Bethesda, MD 20892, USA; College of Wildlife and Protected Area, Northeast Forestry University, Harbin 150040, China; The Vertebrate Genome Lab, The Rockefeller University, New York, NY 10065, USA; Tree of Life, Wellcome Sanger Institute, Cambridge CB10 1SA, UK; Genome Informatics Section, Computational and Statistical Genomics Branch, National Human Genome Research Institute, National Institutes of Health, Bethesda, MD 20892, USA; Laboratory of Neurogenetics of Language, The Rockefeller University, New York, NY 10065, USA; Howard Hughes Medical Institute, Chevy Chase, MD 20815, USA; School of Biological Sciences, The Environment Institute, The University of Adelaide, Adelaide 5005, Australia; The MOE Key Laboratory of Biosystems Homeostasis & Protection and Zhejiang Provincial Key Laboratory for Cancer Molecular Cell Biology, Life Sciences Institute, Zhejiang University, Hangzhou 310058, China; Center for Reproductive Medicine, The 2nd Affiliated Hospital, School of Medicine, Hangzhou 310052, China; Centre for Evolutionary & Organismal Biology, Zhejiang University School of Medicine, Hangzhou 310058, China; Centre for Evolutionary & Organismal Biology, Zhejiang University School of Medicine, Hangzhou 310058, China; Liangzhu Laboratory, Zhejiang University Medical Center, Hangzhou 311121, China; Women’s Hospital, School of Medicine, Zhejiang University, Hangzhou 310006, China

**Keywords:** echidna, monotreme, sex chromosome evolution, multiple sex chromosomes

## Abstract

**Background:**

A thorough analysis of genome evolution is fundamental for biodiversity understanding. The iconic monotremes (platypus and echidna) feature extraordinary biology. However, they also exhibit rearrangements in several chromosomes, especially in the sex chromosome chain. Therefore, the lack of a chromosome-level echidna genome has limited insights into genome evolution in monotremes, in particular the multiple sex chromosomes complex.

**Results:**

Here, we present a new long reads–based chromosome-level short-beaked echidna (*Tachyglossus aculeatus*) genome, which allowed the inference of chromosomal rearrangements in the monotreme ancestor (2n = 64) and each extant species. Analysis of the more complete sex chromosomes uncovered homology between 1 Y chromosome and multiple X chromosomes, suggesting that it is the ancestral X that has undergone reciprocal translocation with ancestral autosomes to form the complex. We also identified dozens of ampliconic genes on the sex chromosomes, with several ancestral ones expressed during male meiosis, suggesting selective constraints in pairing the multiple sex chromosomes.

**Conclusion:**

The new echidna genome provides an important basis for further study of the unique biology and conservation of this species.

## Introduction

An understanding of chromosome evolution has been fundamental for mammalian comparative studies [1, 2]. Large-scale chromosomal rearrangement is an important source of genetic variation and has contributed to adaptation and speciation, and dissection of the underlying mechanisms requires high-quality genomes [3–5]. High-quality genomes are also an important basis for understanding species biology and for long-term application in species conservation [6–9]. Monotremes, including platypus (*Ornithorhynchus anatinus*) and 4 echidna species (Tachyglossidae), comprise the sister group of therians and the most basal mammalian lineage. Due to their unique phylogenetic position in mammal evolution, these species hold the key to understanding the evolutionary changes of major mammalian lineages since their divergence from the common ancestor with other mammals [9–11]. In addition, monotremes are iconic in Australia, and much of their extraordinary biology is still unexplored. These species have a karyotype with 7 or 8 pairs of large chromosomes and many small chromosomes reminiscent of the microchromosomes in reptiles but of different origins [12, 13]. Compared to therians, the monotreme karyotypes are highly rearranged [14]. Thus, the monotreme genomes are valuable for gaining the insight of mammalian and monotreme genome evolution, as well as understanding the changes in genome architecture of reptiles and mammals.

One of the most remarkable features of the genome of egg-laying mammals is their special sex chromosome system, consisting of multiple X and Y chromosomes. In most sex chromosome systems, the sex chromosomes typically exist as 1 pair, with recombination suppression often initially driven by intrachromosomal rearrangement such as inversion [15, 16]. In some lineages, one of the sex chromosomes would fuse with an autosome, leading to a trivalent sex chromosome system. For example, in the male Japan Sea stickleback, the ancestral Y is fused with an ancestral autosome LG9, resulting in a X1X2Y system where the X1 is the ancestral X chromosome (LG19) and X2 is the neo-X chromosome resulting from the fusion event (LG9) [17]. Such fusions may offer evolutionary advantages, such as enabling sex-biased inheritance of genes favored by 1 sex [18] and driving speciation events [17]. However, it may also introduce difficulty in pairing and segregating the multiple sex chromosomes into offspring cells [19] and disrupting gene expression during spermatogenesis due to meiotic sex chromosome inactivation [20]. The evolutionary process of the multiple sex chromosomes in monotremes and its consequences can be even more complicated. In males, there are 9 (echidna) or 10 sex chromosomes (platypus), pairing in a head-to-tail manner via the pseudoautosomal regions (PARs) and forming a meiotic chromosome chain [21–23]. It is established now that this system originated independently from the therian XY sex chromosome system [10, 23] and probably evolved via a series of reciprocal translocation events between the proto sex chromosomes and autosomes [9, 10, 24]. Therefore, the upstream sex-determinant genes are distinct between the 2 mammalian groups, with *SRY* being the key player in therian mammals and *AMHY* being the most likely candidate in monotremes [10]. This complex system has furthermore undergone independent evolution after the 2 species diverged from each other. There are 5 Xs and 5 Ys in male platypus, while there are 5 Xs but only 4 Ys in male echidna [23]. Of these chromosomes, the third Y and the fourth X chromosomes (Y3, X4) of platypus and the fifth X chromosome (X5) of echidna are homologous to the autosome in the other species [23] and are considered to evolve via reciprocal translocation after their speciation [25]. Therefore, the complicated system in monotremes serves as a model example to demonstrate the unusual driving force of high-frequency reciprocal translocations during sex chromosome evolution and the resulting constraint of the multiple sex chromosome system, such as the need to successfully segregate multiple X and Y into different sperms.

Previously, we have tracked the evolution of the monotreme genome, particularly the sex chromosome with a chromosome-level platypus genome and a draft echidna genome [9]. However, the draft echidna genome, especially the Y chromosomes, is still incomplete and largely fragmented in sequence. These 2 major lineages in monotremes diverged around 55 million years ago [9], with an average dS value in coding regions at around 0.1907, implying a substantial divergence in their genetic properties. A more complete echidna genome is to provide a more comprehensive understanding on the evolution across major mammal groups and the divergence within the monotreme lineage. In this study, we produced an improved chromosome-level short-beaked echidna *Tachyglossus aculeatus* (NCBI:txid9261) assembly to further explore the genomic features of these young and unusual sex chromosomes. We also conducted the first genome-wide screen of the ampliconic genes on the monotreme sex chromosomes, unveiling potential selection constraints on the multiple sex chromosome systems.

## Results

### A chromosome-level short-beaked echidna genome

**Figure 1:**
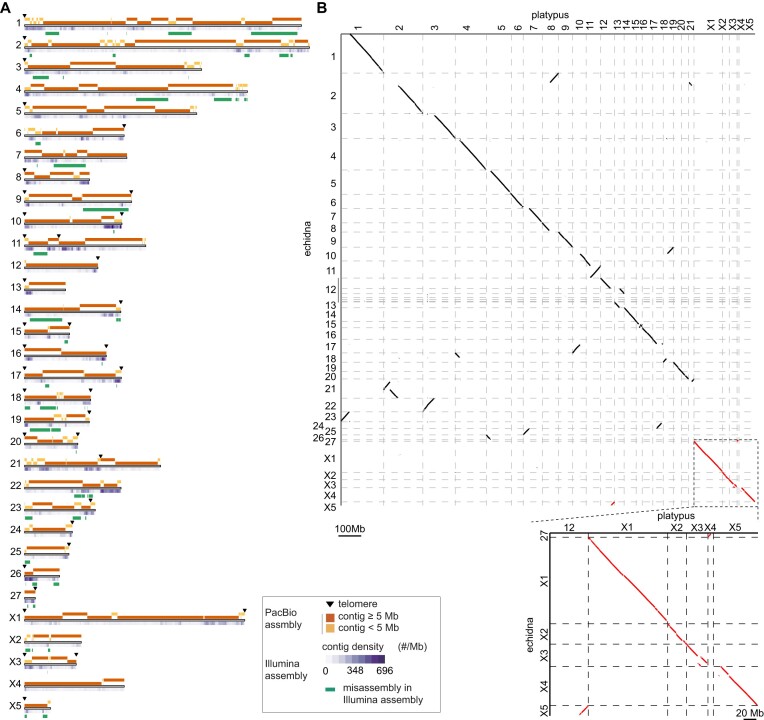
Genome assembly of short-beak echidna. (A) Schematic plot mapping of the assembled contigs onto echidna chromosomes. Orange rectangles on top represent contigs ≥5 Mb in the new assembly, and yellow rectangles represent contigs <5 Mb. The heatmap represents the density of contigs in Illumina-based assembly mapping onto chromosome counting based on the number of contigs per 1-Mb region. Assembled telomere sequences are shown in black triangles in the plot. Coordinates of Illumina-based assembly artifacts corrected in the PacBio-based assembly are shown in green. (B) Dotplot showing the genome synteny between platypus and echidna. The overall synteny (86.94%) is well kept between the 2 species, but there are still 2.60% and 10.46% intra- and interchromosomal rearrangements, respectively. Zoom-in alignment shows that most sex chromosomes are in high synteny and homology, except platypus X4 and echidna X5, which are homologous to the autosome in the other species.

We utilized PacBio long reads, 10X-linked reads, and Bionano and Hi-C data to produce the chromosome-level genome assembly for a male short-beaked echidna, following the VGP assembly pipeline v1.6 (Supplementary Tables S1 and S2). Briefly, PacBio long reads were first used to construct contigs, and scaffolds were generated iteratively with 3 scaffolding technologies (i.e., 10X, Bionano, and Hi-C). We further identified the sex-linked sequence based on the sequencing depth difference between male and female. The new PacBio-based assembly includes 27 autosomes and 5 X and 4 Y chromosomes, with a ∼966-fold improvement on contig N50 compared to the published short read–based assembly (GCA_015598185.1) (Supplementary Table S2). Telomeres have been assembled on 28 of the total 32 chromosomes (Fig. 1A, Supplementary Table S3). Notably, 183.44 Mb and 9.18 Mb of the X and Y sex-differentiated regions on the 5 X (X-Div, X divergent) and 4 Y chromosomes (Y-Div, Y divergent), respectively, were identified (Supplementary Table S4, Supplementary Fig. S1). We also utilized the Hi-C data to filter and infer the possible chromosome origin for previously unplaced X, Y, and PAR scaffolds (Supplementary Table S5, Supplementary Fig. S2). In summary, 99.82% and 98.25% of the assembled X-Div and Y-Div sequences can be assigned to the 9 sex chromosomes, representing a more continuous and complete sequence compared to the previous assembly (Supplementary Table S2). Based on the estimation from karyotype images in Rens et al. [23], we found that most of the 9 chromosomes have over 98% completeness except Y3 (21.34%) and X5 (22.44%), which have accumulated exceptionally high repeat contents [23] (Supplementary Table S6). Evaluation by male-specific transcripts [10] also showed that all male-specific genes were fully covered, except only 1 was fragmented (coverage <50%) in the new PacBio-based assembly (Supplementary Table S7). In contrast, 2 were fragmented and 3 were missing in the previous assembly (Supplementary Table S7).

Alignment between the new and old echidna assemblies (PacBio-based GCA_015852505.1 vs. Illumina-based GCA_015598185.1) revealed 66 large putative structural variants (>100 Kb). Although the sequenced individuals were collected from different locations, these large putative structural variants were likely to be misassembly artifacts in either assembly. Based on the examination of raw PacBio, 10X-linked reads, and Hi-C data, we found that the genome structure of 65 regions was correct in the new echidna genome (Fig. 1A, Supplementary Table S8, Supplementary Fig. S3); only 1 was an error in our new assembly, which has been manually fixed in the latest release. Moreover, ∼74.27% gaps or an estimated size of 179.51 Mb sequences in the previous assembly were closed in the new PacBio-based assembly (Supplementary Fig. S4, Supplementary Table S9), contributing to the new annotation of 21,334 exons from 6,493 protein-coding genes. This is consistent with the improved BUSCO evaluation, which shows that 90.80% of the 9,226 mammalian conserved orthologs are complete and presented as a single copy in the PacBio-based assembly, compared to only 59.20% in the Illumina-based one (Supplementary Fig. S4, Supplementary Table S2).

### Genome evolution of platypus and echidna

**Figure 2:**
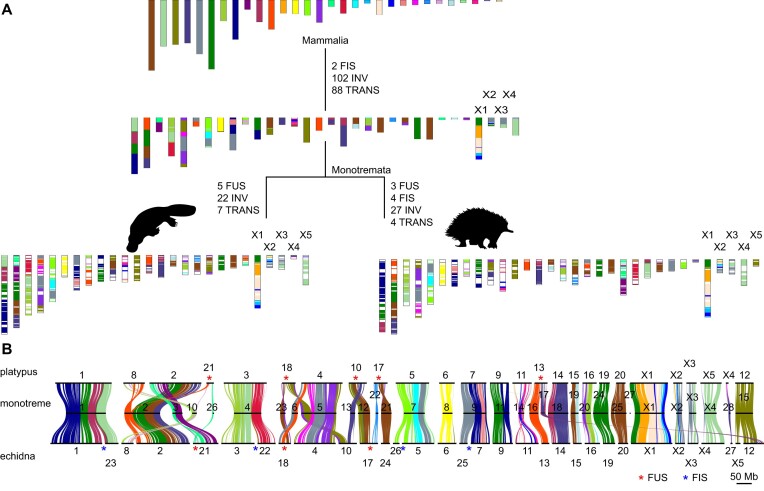
Karyotype evolution of monotremes. (A) 2n = 64 ancestral karyotypes were inferred in the MRCA of monotremes, including 28 pairs of autosomes and 4 pairs of sex chromosomes, under a 300-Kb resolution. Conserved blocks were color-coded with the chromosomal source in the mammalian ancestor. The length of the conserved blocks in the ancestors is taken as the length of the conserved blocks in humans. Numbers of estimated rearrangements are shown for the evolution of monotreme MRCA to the extant species. FUS: fusion; INV: inversion; TRANS: translocation; FIS: fission. A more complete reconstruction of karyotype evolution is available in Supplementary Fig. S8, and a similar reconstruction under a 500-Kb resolution is available in Supplementary Fig. S9. The yellow-colored chromosome is the one that evolved into therian X chromosome. (B) Conserved block between the monotreme MRCA and the extant monotremes shows the chromosome rearrangement events during evolution. Alignment of the conserved blocks was color-coded with the chromosomal source in the mammalian ancestor. Fusion (FUS) and fission (FIS) events are marked with red and blue asterisks, respectively.

There are 2n = 63 and 64 chromosomes in male and female short-beaked echidna and long-beaked echidna, respectively, while there are only 2n = 52 chromosomes in platypus [23, 26], suggesting that chromosome fusion or fission events might have occurred since platypus–echidna divergence. Direct comparison between the 2 species uncovered other genomic rearrangements, including inversions and translocations (Fig. 1B, Supplementary Figs. S5–S7). To systematically investigate evolution of the genomic rearrangements during the divergence of monotremes, especially those involved in sex chromosome evolution, we reconstructed the karyotype of the monotreme ancestor with chromosomal assemblies of placentals (human, bovine, and sloth), marsupials (opossum and Tasmanian devil), monotremes (platypus and echidna), and reptilian out-groups (chicken, turtle, and common wall lizard), under a 300-Kb and 500-Kb resolution. Based on the genomic data and the previous fluorescence *in situ* hybridization (FISH) and *in silico* reconstruction [14, 27–29], we inferred an ancestral karyotype of 2n = 64 of the monotremes’ most recent common ancestor (MRCA), including 28 pairs of autosomes and 4 pairs of sex chromosomes. Although this number is closer to the karyotype number of echidna than that of platypus, the echidna genome experienced more lineage-specific rearrangement than platypus (Fig. 2A, Supplementary Figs. S8 and S9, Supplementary Tables S10–S12). Thirteen monotreme ancestral chromosomes (MON8, 11, 14, 18, 19, 20, 24, 25, 28, and X1–4) were preserved as individual chromosomes in both species; some have experienced genomic rearrangement events in either or both species, while others have experienced genomic rearrangement events in either or both species (Fig. 2B). For example, the breakage of MON4 produced 2 echidna chromosomes while it has remained intact as chr3 in platypus; the fusion of MON12 and MON22 produced 1 echidna chromosome while remaining separate as chr10 and chr17 in platypus (Fig. 2B). These interchromosomal rearrangements were consistent with the previous findings by FISH [23]. However, the whole genome alignment also provided refined details in intrachromosomal rearrangements. For example, the echidna chr11 and chr21 experienced intrachromosomal inversion after divergence from platypus, indicated by both the ancestral reconstruction (Fig. 2B) as well as the telomere remnant at the inversion breakpoints (Supplementary Fig. S7). Interestingly, the centromere monomer sequences of the 2 species are distinct [9], probably associated with the chromosomal rearrangements. Furthermore, recent studies of vertebrate chromosome evolution suggested that the avian microchromosomes can be dated back to the ancestor of the amniote [30], and the mammalian macrochromosomes likely evolved by a series of chromosome fusions and translocations [31]. Our reconstruction confirmed this inference by finding that each chicken microchromosome can be mapped to 1 mammalian ancestral chromosome (Supplementary Fig. S10).

The ancestral karyotype reconstruction also provides novel insight into the dynamic evolution of the monotreme sex chromosome complex. Four of the 5 extant sex chromosomes (platypus chrX1–X3, chrX5 and echidna chrX1–X4) were established in the MRCA (Fig. 2B) [23]. The lineage-specific sex chromosomes (i.e., platypus X4 and echidna X5) originated independently from 2 different ancestral autosomes (Fig. 2B), as initially reported by cross-species *in situ* hybridization [23]. Specifically, MON28 is maintained as a single autosome chr27 in echidna but becomes chrX4 in platypus (Fig. 2B). MON15 remained as a single chromosome chr12 in platypus but was separated into the echidna chr12 and chrX5 (Fig. 2B).

### Monotreme sex chromosomes have both shared and independently formed evolutionary strata

Our previous work suggested that the multiple sex chromosome system in platypus evolved from an ancestral chromosome ring structure via a series of reciprocal translocations between proto-sex chromosomes and autosomes [10, 24] as well as chromosome fusions [9]. Among the 5 pairs of monotreme sex chromosomes, 4 are shared between platypus and echidna, but how each monotreme lineage evolved their distinct sex chromosome complex after they diverged from their common ancestor 55 million years ago (MYA) remains to be elucidated [9]. By projecting our ancestral karyotype reconstruction to the platypus and echidna sex chromosomes, we found that the monotreme ancestral sex chromosomes (i.e., echidna X1–X4 and platypus X1–X3, X4) consist of homologous fragments from different ancestral chromosomes (Supplementary Fig. S11a, Supplementary Tables S11 and S12) [9]. Specifically, parts of each 2 neighboring sex chromosomes are homologous to 2 adjacent regions of the same ancestral chromosome (Supplementary Fig. S11a), forming the PARs and the sex-differentiated regions (SDRs). This suggests that a high number of translocations occurred before the monotremes evolved their extant sex chromosome configuration. The species-specific sex chromosomes (i.e., platypus Y3, X4 and echidna X5) originated from different mammalian ancestral chromosomes (MAMs) (Supplementary Tables S11 and S12). Consistent results could be confirmed by the projection using the chicken genome (Supplementary Fig. S11b, Supplementary Table S13).

**Figure 3:**
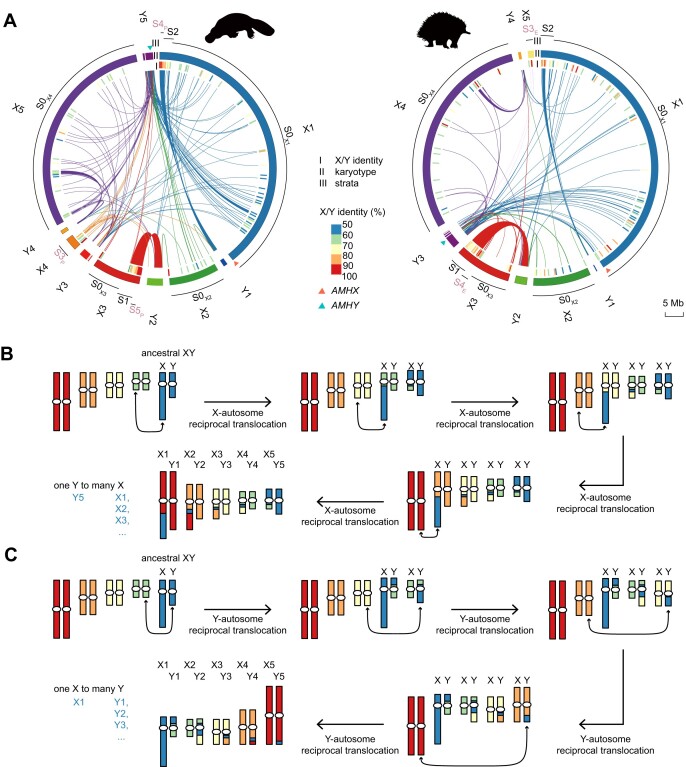
X/Y sequence alignment and the 2 possible reciprocal translocation scenarios in multiple sex chromosome evolution. (A) Tracks from inside out (I–III): X/Y identity, karyotype (PAR excluded), strata. The orthologous chromosomes, echidna Y3 and platypus Y5, are homologous to multiple X chromosomes in both species, including X1, X2, and X3 and echidna X4 (platypus X5). The species-specific sex chromosome is homologous to the sex chromosome it paired with during meiosis. Three strata (S0–S2) are ancestral (black) while the younger 3 (S3–S5) evolved independently in the 2 lineages (brown). Only assigned X and Y are shown. Independent strata are marked with a subscript “P” or “E” indicating the strata evolved in platypus or echidna, respectively. (B, C) Evolution of the sex chromosome chain by a series of reciprocal translocations between ancestral autosomes and X (B) or Y (C). (B) The reciprocal translocation between the ancestral X and the ancestral autosomes will distribute the ancestral X to the ancestral autosomes, resulting in a “one Y to many X” homology relationship in the end. (C) The reciprocal translocation between the ancestral Y and the ancestral autosomes will distribute the ancestral Y to the ancestral autosomes, resulting in a “one X to many Y” homology relationship in the end. Based on our observation in platypus and echidna, the translocation between autosomes and X is more possible for the evolution in monotreme sex chromosome evolution.

In many species, sex chromosome evolution is characterized by stepwise recombination suppression, which would lead to the stratified pattern of different sequence divergence levels between X and Y sex-differentiated regions termed “evolutionary strata” along the sex chromosome [10, 16]. Previously, we inferred 7 strata in the sex chromosome chain by X/Y gametologs and their phylogeny [9], but this could be impacted by the limited number of gametolog pairs and possible gene conversion between the pair [32]. Here with more gametolog pairs from the more complete echidna genome, we found that the pairwise dS values between gametolog pairs in the previously identified S0–S4 strata located on the X1–X4 chromosomes did not show significant differences (Supplementary Fig. S12a). Interestingly, among these X/Y gametolog pairs, over 80% of the Y gametologs are located on the 1 Y chromosome echidna Y3 or its homologous platypus Y5 [23] (Supplementary Fig. S13), respectively (Supplementary Tables S14–S16). The X/Y sequence alignments also revealed that the echidna Y3 (or platypus Y5) exhibit the largest (>60%) aligned region on X1, followed by smaller alignments with X2, X3, and X4 (or platypus X5) (Fig. 3A, Supplementary Table S17); in contrast, the other Ys are mostly homologous to their neighboring Xs (Supplementary Table S17). On the other hand, we have not found 1 X chromosome that exhibits as many alignable fragments to many Ys. Instead, when excluding echidna Y3 and platypus Y5, all X chromosomes are aligned most to their neighboring Ys (Supplementary Table S17). Such a pattern of “one Y to many X” can be achieved only via a series of autosome-X translocation (Fig. 3B) instead of autosome-Y translocation, which may produce the opposite “one X to many Y” result (Fig. 3C). Notably, we found that *AMHX* in platypus should locate near the end of chrX1 (Supplementary Fig. S11) and in the same syntenic region as in echidna (Fig. 3A), instead of our previous inference at the middle part of X1 [9].

**Figure 4:**
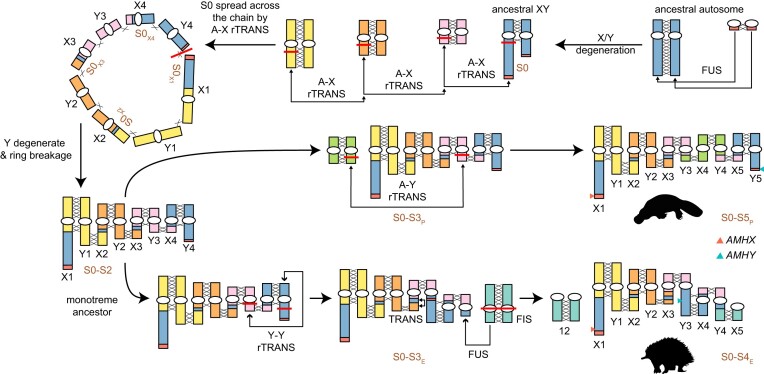
Model for the sex chromosome evolution in monotremes.

Both the X/Y divergence and X/Y homology pattern suggest an alternative monotreme sex chromosome evolution model contrary to our previous hypothesis that recombination suppression happened after reciprocal translocations. Instead, the recombination suppression might have already been initiated on the ancestral X (X1) and Y (echidna Y3 or platypus Y5) in the monotreme ancestor to form the ancestral stratum S0. Subsequently, a series of translocations between the nonrecombining X and autosome occurred, producing the scattered homology between 1 ancestral Y and 4 ancestral X chromosomes (except for the echidna X5 and platypus X4), leaving similar dS levels of X/Y gametologs across different X chromosomes (Fig. 4). In addition, by distributing the ancestral nonrecombining X to different chromosomes, the pairing Y chromosome can no longer recombine with the X-counterpart (e.g., during meiosis, echidna Y3 only pairs with X3 and X4 but not X1 and X2), leading to the accumulation of deleterious mutations on the Y chromosomes. Moreover, such reciprocal translocations may also initiate the recombination suppression between the neighboring sex chromosomes (e.g., X2–Y2), creating gametologs with younger ages and unlikely to be involved in sex determination. Under such a scenario, we proposed that there were at least 6 and 5 evolutionary strata in platypus and echidna, respectively; the oldest 3 evolved ancestrally in the monotreme MRCA, while the youngest 3 or 2 evolved independently in the 2 lineages (Fig. 3A, Supplementary Figs. S12b, S14, and S15; Supplementary Tables S14 and S16). The oldest stratum S0 was delineated to be distributed across all 4 ancestral X chromosomes (named by their extant residing chromosomes as echidna S0_X1_–S0_X4_ and similarly in platypus). According to the gametolog phylogeny while controlling for gene conversion (Supplementary Fig. S14, Supplementary Table S18) and that both X and Y are from different chromosomes, we considered that S1 (X2–Y2) and S2 (X1–Y1) derived from different MAMs as different strata but formed in the monotreme ancestor. An additional translocation further occurred in echidna, leading to a synteny disruption between the 2 monotremes (see below).

The evolution of the sex chromosome complex in monotreme MRCA involves both chromosome fusion and reciprocal translocation between ancestral autosomes or between ancestral autosomes vs. 1 pair of ancestral XY chromosomes and the oldest stratum S0 that evolved. The translocation distributes the ancestral X segments and S0 into many ancestral autosomes, results in a “one Y to multiple X” homology relationship, and possibly forms a ring structure. The Y sequence degeneration further inhibits the pairing and breaks the ring into a chain. S1 and S2 later evolved in the monotreme ancestor and caused PAR erosion. The system then undergoes different evolutionary trajectories between platypus and echidna by recruiting different autosomes into the complex after they split. In platypus, a reciprocal translocation happened between autosomes and ancestral Y3, recruited the autosomes into X4 and part of Y3/Y4, and evolved into its independent stratum, S_P_. In echidna, a Y3–Y4 reciprocal translocation happened and altered the X/Y pairing order. The ancestral Y4 in echidna further experienced chromosome fusion with part of autosome sequences and formed S3_E_. Additional translocation also happened in echidna X3, disrupting its synteny (including S1 and S4_E_) with platypus. Recombination suppression further happened independently in platypus and echidna on X1 and X3 and formed the youngest 2 strata. The coordinates of the putative sex-determining gene *AMHX/Y* are also labeled in platypus and echidna (rTRANS: reciprocal translocation; TRANS: translocation; FUS: fusion; FIS: fission; A, autosome). Different ancestral chromosomes are filled with different colors.

Among 3 younger strata (S3–S5) that evolved independently in the 2 species, S3_P_ (platypus S5) and S3_E_ (echidna S5) are located on the species-specific X (i.e., platypus X4 and echidna X5, respectively) [23, 25], although the support of independent evolution from gametolog phylogeny is ambiguous (Supplementary Fig. S14c, Supplementary Table S18). Previous studies and the above ancestral karyotype reconstruction showed that the species-specific X chromosomes of these 2 species are homologous to an autosome in the other species, thus providing a unique model to study the lineage-specific genomic changes involved in the sex chromosome evolution. In echidna, 88.81% of the assembled X5 shows a similar sequencing depth between male and female (Supplementary Fig. S16a), which indicates this is a recently evolved X chromosome and only contains a small nonrecombining region. Nine genes reside in the remaining 1.8 Mb X-Div on X5 (Supplementary Fig. S17a). Interestingly, an ∼300-Kb inversion was identified between the X-Div region of echidna X5 and its orthologous region in platypus chr12, spanning 1 gene *TACR3* (Supplementary Fig. S17a). This inversion may have contributed to one of the recombination suppressions on echidna X5 (Supplementary Fig. S17c) and led to the degeneration of its Y counterpart. In humans, *TACR3* resides on the autosome, encodes receptors for neurokinin B, and is associated with hypogonadotropic hypogonadism [33]. In both humans and platypus, the gene is mainly expressed in somatic tissues, but in echidna, the gene shows the highest (though not specific) expression in testis (Supplementary Fig. S18), suggesting recent adaptation for a testis-related function. The remaining X-Div on echidna X5 is homologous to a platypus scaffold (scaffold_344_arrow_ctg1) located on platypus X3 by our Hi-C analysis (Supplementary Figs. S2 and S16a) and only contains genes encoding olfactory receptors and vomeronasal receptors (Supplementary Fig. S17a). Thus, in addition to the previous FISH experiment showing that echidna X5 is mapped to the platypus chr12 [23], our observation here suggests that the evolution of echidna X5 may also involve some rearrangement with a part of the ancestral X3. We found longer X/Y alignment remained in the region homologous to platypus scaffold_344_arrow_ctg1 (5,239 bp, 0.95% of the X-Div) than that in the inversion region (1,000 bp, 0.25% of the X-Div), while the sequence divergence level is similar between the 2 regions (2-sided Wilcoxon rank-sum test, *P* = 0.8571). Thus, we hypothesized that echidna X5 first experienced an inversion on the X, then fused with the monotreme ancestral X3 sex chromosome. We also performed a similar analysis to platypus X4 (Supplementary Text). In contrast to echidna X5, platypus X4 did not undergo such inversion. The recombination suppression on X4 started at the chromosome end distant to the current PAR and eroded to the current boundary (Supplementary Fig. S17).

Platypus’ second youngest stratum, S4_P_, is located in X1, where the orthologous region in echidna remains as PAR (Supplementary Fig. S15). The youngest platypus and echidna strata S5_P_ and S4_E_ are located near the respective PAR boundary of the ancestral X3 with supports from various gametologs (Supplementary Fig. S15, Supplementary Table S18). Interestingly, besides an overall high level of synteny between platypus and echidna of the ancestral Xs (Fig. 1B), we identified 1 translocation on X3 between the 2 species. Such translocation spans 2 strata, the ancestral S1 and S5_P_ (or S4_E_) (Fig. 3A, Supplementary Fig. S19a), with a length of at least 4.5 Mb and 29 protein-coding genes. This pattern, as well as our ancestral karyotype reconstruction (Supplementary Tables S8 and S9) and alignment with other mammals, demonstrated that the translocation is more likely to happen specifically in echidna (Supplementary Fig. S19b).

Based on these observations and Dohm et al. [25], we also proposed a model to explain the evolution of the complex sex chromosome system in monotremes after the platypus–echidna split (Fig. 4). After speciation, in platypus, a reciprocal translocation may have happened between an autosome and the ancestral Y3, creating its X4–Y4 containing a new stratum, S3_P_. In echidna, an ancestral Y3–Y4 translocation first happened to exchange the pairing relationship with X. This follows a chromosome fission of an ancestral autosome and a Y-autosome fusion to form the current chr12 and Y4, recruiting the extant X5 into the sex chromosome system similar to the case of neo-X evolution in *Drosophila miranda* [34], and create its specific S3_E_. The young strata (S4_P_, S5_P_ in platypus and S4_E_ in echidna) further evolved independently in the 2 species. A translocation also happened on echidna X3, changing the genomic coordinate of 2 strata (S1 and S4_E_). Based on X/Y sequence divergence, we estimate the ages of the evolutionary strata. The multiple sex chromosome started during the very first recombination suppression on the ancestral sex chromosome at approximately 80 MYA (Supplementary Table S19), followed by spreading the ancestral X fragments across the complex via a series of X-autosome translocations. The species-specific X (platypus X4 and echidna X5) stopped its recombination around 19 and 27 MYA, respectively (Supplementary Table S19).

### The evolution of sex-linked ampliconic genes

One of the notable features of the sex chromosome is that some genes have undergone amplifications to produce highly identical (>99%) copies termed ampliconic genes (AGs) [35]. These genes have been observed to be organized as tandem arrays [36, 37] or inverted repeats described as palindromes [38]. Previous studies have revealed the existence of AGs in both X and Y chromosomes in therian and the Z chromosome in chicken [23, 38–42], as well as on the recently evolved X and Y chromosomes of *D. miranda* [43]. However, to date, only limited information about genome architecture is available for the Y chromosomes of the egg-laying mammals [10]. Utilizing the gene annotation from the long-read assemblies and the male sequencing depth information, in platypus and echidna, we found 10 and 5 X-linked AGs and 12 and 11 Y-linked AGs, respectively (Supplementary Tables S20 and S21), in contrast to the large number of ampliconic genes in eutherian mammals and chicken [40, 44]. Our platypus and echidna Y-linked AG dataset each contains 3 and 4 types of newly reported Y-linked AGs (Supplementary Tables S20 and S21). However, these AG numbers might be underestimated because some may have been collapsed during the genome assembly. As found in human, great ape, mouse, and chicken, in monotremes, both X and Y AGs were found to be predominantly expressed in testis (Supplementary Table S22), consistent with the previous finding from a small subset of these families [10]. Interestingly, only a few of them also testis-specific were expressed in human, suggesting that most of the genes were masculinized in monotremes only after becoming sex-linked (Supplementary Table S23).

**Figure 5:**
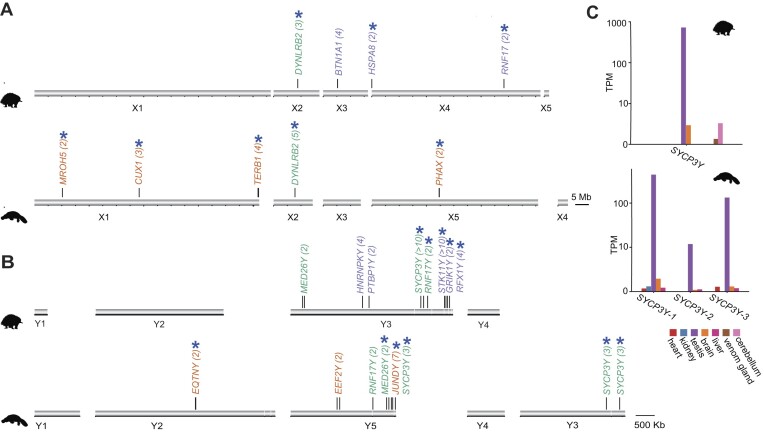
Ampliconic genes in monotremes. (A) Distribution of ampliconic genes in echidna (purple) and platypus (orange) X chromosomes. Green, genes that are ampliconic in both species. The estimated copy number for each ampliconic gene is shown in parentheses. Ampliconic genes with testis-specific expression are marked by asterisks. Homologous chromosomes are shown in the same column. (B) Distribution of ampliconic genes in echidna (purple) and platypus (orange) Y chromosomes. Green, genes that are ampliconic in both species. The estimated copy number for each ampliconic gene is shown in parentheses. Ampliconic genes with testis-specific expression are marked by asterisks. Homologous chromosomes are shown in the same column. (C) Testis-specific expression pattern of ampliconic gene *SYCP3Y* in both echidna and platypus.

Similar to the observation in the comparison between human and mouse ampliconic genes, in monotremes, most ampliconic genes were independently amplified after their divergence about 55 million years ago [9] (Fig. 5A, B). Only 1 X-linked (*DYNLRB2X*s) and 3 Y-linked (*SYCP3Y*s, *RNF17Y*s, and *MED26Y*s) AGs were shared between echidna and platypus. As expected, all these shared X-linked and Y-linked AGs are located on the ancestral sex chromosomes shared by platypus and echidna. The AGs shared between the 2 monotremes should have evolved in their common ancestor and are likely to be important for both species and have been maintained through the degeneration process of the Y chromosomes. For example, we found the Y-linked AG *SYCP3Y* is amplified in both platypus and echidna. *SYCP3Y*s is thought to evolve from an autosomal copy *SYCP3* [10], which encodes protein to form the synaptonemal complex at meiotic prophase I [45]. In this study, we further confirmed that such duplications from autosomes were ancestral in monotreme MRCA at the early stage of sex chromosome evolution (*SYCP3–SYCP3Y* dS ∼0.7, Supplementary Table S24, Supplementary Fig. S20). Interestingly, monotreme *SYCP3Y*s shares a higher sequence identity with *SYCP3* in other mammals than its autosomal paralog *SYCP3* and harbors a newly evolved motif that enables self-association and normal function in the synaptonemal complex [46]. Both *SYCP3Y*s are expressed predominantly in testis (Fig. 5C; Supplementary Tables S20 and S21). Many proteins that act in meiotic and postmeiotic cells are highly transcribed in premeiotic cells. Analysis of the platypus spermatogenesis single-nucleus RNA sequencing data [47] revealed that *SYCP3Y*s is mainly expressed in spermatocytes, which are in the meiosis I stage, where the sex chromosomes are paired and chained [48] (Supplementary Fig. S21). It may be that the amplified *SYCP3Y* genes evolved a male-specific function at meiosis associated with the formation of the complex sex chromosome chain. We hypothesized that these amplifications may be due to the need for the unique pairing and segregation of the multiple sex chromosomes during male meiosis [21, 22].

## Discussion

A high-quality genome is important for the understanding of evolution, particularly the sex chromosome since it is difficult to sequence and assemble [8]. Analysis on monotreme genomes has revolutionized our understanding of mammalian sex chromosome evolution, but we still lack a good understanding of how the complex monotreme sex chromosome system evolved. Here we presented an improved chromosome-level short-beaked echidna assembly constructed from the latest sequencing technologies. This enables us to reconstruct the monotreme ancestral karyotype and investigate the evolutionary trajectory of monotreme sex chromosomes in unprecedented detail. With the newly improved echidna assembly, we explored the different evolution trajectories of the specific species and the ancestral sex chromosome in greater detail than was possible before. The discovery of homologies for echidna Y3 and platypus Y5, with multiple X chromosomes and other Ys homologous only to their neighboring Xs, supports the idea of reciprocal translocations between the ancestral autosomes and the ancestral X instead of the Y [24, 49]. In addition, no significant difference in dS (or X/Y identity) is found among the gametologs on the 4 ancestral X chromosomes (Supplementary Fig. S12), suggesting that the sex chromosome has already diverged ancestrally and the ancestral evolutionary stratum was spread across the chain via a series of autosome-X reciprocal translocations (Fig. 4).

Multiple sex chromosome systems have been found in a variety of species, including therians, avians, frogs, insects, and plants, forming chain- or ring-like structures [24, 50–52]. These systems are typically composed of 3 (trivalent) or 4 (quadrivalent) chromosomes resulting from 1 or 2 translocation events. In contrast, the monotreme sex chromosome complex evolved over 80 million years, with recent changes after platypus and echidnas diverged. Such a complex requires precise formation of a chain at meiosis and alternate segregation. Indeed, previous studies have shown that the sex chromosome chain is assembled in an order starting from Y5 and ending with X1 during meiosis [53]. In addition, dynamic cohesin was observed in platypus prophase I, where the protein differentially loaded at the paired and unpaired regions [54]. Here, we found gene family expansion signals potentially arising from the evolution of the multiple sex chromosome system. We confirmed *SYCP3Y* amplification in platypus [46] and also found such amplification in echidna, suggesting that the gene expansion is ancestral and may be associated with the evolution of the sex chromosome complex or play a role in its organization. Interestingly, pairwise dS values between *SYCP3* and *SYCP3Y* are around 0.7, which falls within the range of the pairwise dS value of the S0 gametologs (Supplementary Table S15). This suggests that the duplication of *SYCP3Y* from *SYCP3* likely occurred around the same time as the early divergence of the ancestral X and Y chromosomes, predating the reciprocal translocation events. It is possible that the preadaptation by *SYCP3Y* acquisition facilitated the reciprocal translocations in sex chromosome evolution by fulfilling the requirement for alternate segregation of the sex chromosome complex. Ampliconic genes have been discovered on the sex chromosomes of many other species, such as mammals and fruit flies [40, 43]. Several evolutionary processes, including male beneficial mutation and meiotic conflict, have been proposed as the cause for this genomic event [43]. In monotremes, the special need for pairing and segregation of the multiple sex chromosome system in males may provide additional evolutionary drive to gene amplification.

In conclusion, our results provide a comprehensive evolutionary history of monotreme sex chromosomes and uncover novel aspects of their genetic composition, including sex-linked gene amplification. Future work still needs to uncover the mechanisms of alternative segregation and sex-specific function of genes, particularly those that have undergone ampliconic expansion. Expression of those genes at specific stages in spermatogenesis is indicative of reproductive function. This new and more complete echidna genome will continue to refine our understanding of sex chromosome evolution, organization, and function in monotremes and other mammals.

## Methods

### Sample collection, genome sequencing, assembling, and sex-linked sequence identification

Echidna sample Emale12 was collected under AEC permits S-492006, S-032–2008, and S-2011–146 at Upper Barnard River (New South Wales, Australia) during the breeding season, and the muscle sample was frozen into liquid nitrogen and used for PacBio sequencing. Other echidna genomic sequencing data, including 10X, Bionano, and Hi-C, were obtained from Zhou et al. [9]. The genome was assembled following the VGP assembly pipeline v.1.6. Genome completeness was evaluated using BUSCO (v5.7.1) (RRID:SCR_015008) [55] and compleasm (v0.2.6) [56], a faster and more accurate reimplementation of BUSCO, with mammalia_odb10 as the database. Male and female Illumina short reads were obtained from NCBI (male: PRJNA576333; female: PRJNA202404) and mapped to the genome using BWA MEM (v0.7.17) [57]. Sex-linked sequences were identified with the same procedure described in Zhou et al. [9]. Briefly, male and female Illumina short reads were mapped to the new echidna assembly using BWA MEM with default parameters. Coverage was extracted with samtools (v1.9) (RRID:SCR_002105) [58], normalized by the peak coverage, and was then calculated in 5-kb windows with bedtools (v2.29.2) (RRID:SCR_006646) [59]. Scaffolds (>10 kb) of over 60% of windows with normalized F/M coverage ratio between 1.5 and 2.5 were identified as X-linked and between 0.0 and 0.3 as Y-linked. Coverage of candidate X- and Y-linked scaffolds was also visualized with ggplot2 (v3.3.5) (RRID:SCR_014601) and manually examined to delineate the PAR within each scaffold. In addition, we further removed possible false positives of the unplaced sex-linked scaffolds, based on the interaction strength under a 100-Kb resolution obtained from Hi-C, with the same method described in Yang et al. [60]. Briefly, interaction strength between each candidate’s unplaced sex-linked scaffold and the assigned autosome and X/Y were compared. We only kept the unplaced sex-linked scaffolds if its interaction with the assigned X/Y was significantly higher than that with the assigned autosome (1-sided Wilcoxon rank-sum test). We also visualized the Hi-C maps of each of these scaffolds and their assigned chromosomes with the hicexplorer package (v3.7.2) (RRID:SCR_022111) [61] and manually confirmed the results with the maps. The estimated sex chromosome sizes were inferred using the same method as described in Rhie et al. [8]. PARs were included for both X and Y completeness evaluation. For example, X1Y1 PAR and Y1×2 PAR were summed with Y1 Y-Div and compared with the expected Y1 size to evaluate the completeness of Y1. We also collected echidna male-specific transcripts from Cortez et al. [10] to evaluate the completeness of the Y gene dataset. Transcript sequences were mapped to the reference genome with BLAT (v319) (RRID:SCR_011919) [62] with parameter “-fine.” Only mapping results to Y-Div were kept.

### Examination of the Illumina-based assembly gap filling status in the PacBio-based assembly

We used a similar method as Bickhart et al. [63] to identify the gap-filling status in the PacBio assembly. Briefly, 500-bp fragments upstream and downstream of each gap in the Illumina assembly were extracted and then aligned back to the PacBio assembly by BWA MEM (RRID:SCR_010910) [57]. If a gap is too close (<200 bp) to the end of the scaffold or its size is <5 bp, the gap is excluded in further analysis. If both fragments aligned successfully (aligning rate >70%) to the same scaffold in the PacBio assembly and the intervening sequence of the PacBio assembly did not contain any ambiguous base (N), the gap was considered closed. If the 2 fragments were aligned to different scaffolds, the gap was considered a trans-scaffold break. If 1 or both fragments did not align to PacBio assembly, or the intervening sequence contained the ambiguous bases, the gap was considered open.

### PAR identification with Hi-C

The above method of sex-linked sequence identification can only identify PAR, which is assembled with X-Div or Y-Div. We found that 2 PARs (i.e., X3Y3 and Y3×4 PAR) cannot be identified based on the above depth method from the echidna genome. Under the general Hi-C assumption that the intrachromosomal interaction is larger than the interchromosomal interaction [64], we therefore used the Hi-C interaction matrix to identify PAR sequences from the unplaced scaffolds. We assume that, if an unplaced scaffold is X3Y3 (or Y3×4) PAR, its interaction with X3 and Y3 (or Y3 and X4) should be stronger than the interaction with other sex chromosomes and autosomes. Thus, for each unplaced scaffold, we extracted its Hi-C interaction under a 100-Kb resolution with X3, Y3 (or Y3, X4) and compared the dataset with the Hi-C interaction with each other anchored sex chromosomes as well as autosomes. If the unplaced scaffold had a significantly higher Hi-C interaction with X3 and Y3 (or Y3 and X4) than the Hi-C interaction with every other anchored chromosome under the 1-sided Wilcoxon rank-sum test, we considered it the X3Y3 (or Y3×4) PAR. We also tried this method in platypus to identify X4Y4 PAR, but no unplaced scaffolds showed significantly higher interaction with the anchored X4 and Y4 when compared to other chromosomes.

### Comparison between the platypus and echidna assembly

We used lastZ (v1.04.00) (RRID:SCR_018556) [65] to align the new echidna assembly to the platypus assembly with parameter set “–hspthresh=4500 –gap=600,150 –ydrop=15000 –notransition.” Only alignments over 10 Kb were kept for plotting in Fig. 1. Dotplot was generated with the custom Python script. To generate the pairwise alignment between sex-linked sequences, we also performed lastZ alignment between the 2 assemblies, with the parameter set the same as mentioned above and a matrix for closely related species. We confirmed the structural variants between the 2 genomes with PacBio, 10X-linked reads, and Hi-C data. Since the homology between echidna and platypus is not available for all chromosomes [23], in this study, we assigned scaffolds to chromosomes based on the mashmap alignment between the 2 species, except for the sex chromosome, whose nomenclature is based on Rens et al. [23] (Supplementary Table S3).

### Ancestral karyotype reconstruction

We utilized the genomic information to reconstruct the ancestral karyotype of monotremes with a similar method as in Zhou et al. [9]. The *Ornithorhynchus anatinus* genome (GCF_004115215.2) was used as reference and genomes of *Bos taurus* (GCF_002263795.1), *Choloepus didactylus* (GCF_015220235.1), *Gallus gallus* (GCF_016699485.2), *Homo sapiens* (GCA_000001405.28), *Monodelphis domestica* (GCA_000002295.1), *Podarcis muralis* (GCA_004329235.1), *Sarcophilus harrisii* (GCA_902635505.1), *Tachyglossus aculeatus*, and *Trachemys scripta elegans* (GCF_013100865.1) were aligned to the reference using lastZ with parameter set “–step=19 –hspthresh=2200 –inner=2000 –ydrop=3400 –gappedthresh=10000” and a matrix for distantly related species. Genomes were softmasked before running lastZ. Conserved segments among the species were extracted from the NET result with DESCHRAMBLER (git commit 28686dda39144f9d8223dce663aadf0621002643) [29] under a 300-Kb resolution, with the tree obtained from Timetree [66]. We required conserved segments to be uniquely and universally presented in all mammals but allowed segments missing or duplicated in the reptilian out-groups. Ancestral karyotype reconstruction was performed with ANGES (v1.01) [67] for all nodes after mammal radiation, and we further curated the results according to previous reconstruction by FISH or a bioinformatic method [9, 14, 27–29]. We also incorporated pairiwise gene synteny information inferred from MCScanX (RRID:SCR_022067) [68] to link the contiguous ancestral regions (CARs) at monotreme MRCA (Supplementary Table S10). The length of the ancestral chromosome was based on the length of the conserved blocks in human. We also performed a reconstruction under a 500-Kb resolution. The overall results were similar, except that there was no conserved segment for platypus chrX4 and echidna chr27 due to the 500-Kb threshold in monotreme ancestral karyotype reconstruction, and thus MON28 was not available in the result (Supplementary Table S12, Supplementary Fig. S9). Manual curation was performed to link PAR with X/Y-Div, which were separately assembled in the genome. Rearrangement events from monotreme MRCA to extant species were then inferred with GRIMM (v2.1) [69].

### Sex chromosome evolution

#### Chromosome painting with chicken genome sequence

To obtain the orthologous information between monotreme sex chromosomes and chicken genome, we aligned the chicken genome (GCF_016699485.2) to each monotreme genome with lastZ under parameter set “–step=19 –hspthresh=2200 –inner=2000 –ydrop=3400 –gappedthresh=10000” and a matrix for distantly related species. We only kept alignment ≥100 Kb. Gaps between alignment were filled with adjacent alignment results and visualized with ggplot2 (v3.3.6). Since each PAR was assembled in 1 copy in the haploid genome, we duplicated the PAR alignments and placed each with X and Y chromosomes for visualization. Y-linked scaffolds were ordered based on its length during visualization.

#### Confirmation of platypus *AMHX* genomic coordinate

Platypus *AMHX* is not assembled in the genome (GCF_004115215.2) used in this study. To locate the position of *AMHX* on chrX1, we extracted the *AMHX* located scaffold (Contig22983) from another platypus genome (OANA5) and combined it with GCF_004115215.2 to obtain a more complete assembly. Platypus Hi-C reads were aligned to this more complete genome with juicer (v1.6), and a hic file was generated. We split chrX1 into 100-Kb nonoverlapping windows and calculated the sum of the interaction strength (normalized with the SCALE method) of each window under 10 Kb with Contig22983 using straw (v0.08). Juicebox (v1.11.08) (RRID:SCR_021172) was used for Hi-C matrix visualization.

#### Strata

We used a similar method in Zhou et al. [9] to identify the strata in echidna and platypus sex chromosomes. Briefly, repeat annotation was obtained from NCBI; we performed additional repeat annotation using the Tandem Repeat Finder (v4.09) (RRID:SCR_022193) [70] and RepeatMasker (v4.1.0) [71], where the library was generated based on the respective monotreme genome with RepeatModeler (v1.0.8) (RRID:SCR_015027). Repeat in Y-Div and X-Div was then N-masked and aligned with lastZ, and the maf results were used to calculate X/Y identity in 1-Kb windows. We also performed additional lastZ alignment between Y-Div and other genomic regions (autosome + X-Div + PAR). X/Y alignment would be filtered out if the Y segments could be better aligned to autosome/PAR, defined as having a higher identity and longer alignment to autosome/PAR than to X-Div. Circos (v0.69–9) [72] was used to visualize X/Y alignment and sequence identity. X/Y gametolog pairs were identified by BLASTP using the Y gene protein sequences with all X + autosome gene protein sequences. Only Y genes best hit to X genes were kept, and we further examined the gene name to confirm their homology. X/Y gametolog CDS alignment was built using PRANK (v170427) [73], and dS was calculated using PAML codeml (v4.8) [74]. To confirm if the gametolog pairs originated ancestrally or independently in the 2 species, we obtained the protein sequences of the X and Y gametologs, performed multiple sequence alignment by PRANK, converted protein alignment back to CDS alignment, and then constructed each phylogeny tree by RAxML (v8.2.4) (RRID:SCR_006086) [75] with parameters “-f a -x 12345 -p 12345 -# 100 -m PROTGAMMALGX.” Geneconv (1.81a) [76] was used to detect gene conversion signal from the alignment.

#### Species-specific X evolution

The platypus and echidna lastZ result generated above was used here to obtain the alignment of the Xs between the 2 species. Gene distribution on the region was visualized with pyGenomeTracks (v3.7) [77]. N-masked X sequences were aligned to N-masked Y with lastZ under parameter set “–step=19 –hspthresh=2200 –inner=2000 –ydrop=3400 –gappedthresh=10000” and a matrix for distantly related species. We further filtered the alignment to remove the redundancy on X, and on the basis of the “net” and “maf” results, the identity of each alignment block was calculated in 1-Kb nonoverlapped windows. X/Y identity on different regions of echidna X5 and platypus X4 was classified according to the X alignment to the other species, and we performed a 1-sided Wilcoxon rank-sum test if there was a significant difference between the 2 regions. X/Y alignment was also visualized with circos (RRID:SCR_011798) [72] and color-coded according to the Xs.

#### Age calculation of the monotreme strata

We used a similar method as Zhou et al. [15] to infer the age of each stratum. Since the mutation rates of male and female are different, the rate of XY sequence divergence is not the same as the rate of divergence of an autosomal duplication. However, they can be connected by the male mutation rate $\alpha $, which is the ratio of the male and female mutation rates. Assuming the female mutation rate is ${\mu _f}$, the evolutionary rate of different chromosomes is


\begin{eqnarray*}
{\mathrm{A}}:{\mathrm{\ }}\frac{{\alpha + 1}}{2}{\mu _f}
\end{eqnarray*}

\begin{eqnarray*}
{\mathrm{X}}:{\mathrm{\ }}\frac{{2 + \alpha }}{3}{\mu _f}
\end{eqnarray*}

\begin{eqnarray*}
{\mathrm{Y}}:{\mathrm{\ }}\alpha {\mu _f}
\end{eqnarray*}

The divergence rate of autosome and XY is


\begin{eqnarray*}
{\mathrm{Autosome}}:\ \frac{{\alpha + 1}}{2}{\mu _f} + \frac{{\alpha + 1}}{2}{\mu _f} = \left( {1 + \alpha } \right){\mu _f}
\end{eqnarray*}

\begin{eqnarray*}
{\mathrm{XY}}:{\mathrm{\ }}\frac{{2 + \alpha }}{3}{\mu _f} + \alpha {\mu _f} = \frac{{2 + 4\alpha }}{3}{\mu _f}
\end{eqnarray*}

Thus, similar to Ross et al. [41], the ratio of rates of XY and the autosome sequence is


\begin{eqnarray*}
\frac{{2 + 4\alpha }}{3}{\mu _f}/\left( {1 + \alpha } \right){\mu _f} = \frac{{2 + 4\alpha }}{{3 + 3\alpha }}
\end{eqnarray*}

We took the platypus autosomal divergence rate ${\mu _{AA}}$ (i.e., the mutation rate), $7 \times {10^{ - 9}}$/site/year from Martin et al. [78], and the average male mutation bias $\alpha = 2.95$ estimated by Link et al. [79]. The platypus XY divergence rate ${\mu _{XY}}$ is thus $8.15 \times {10^{ - 9}}$/site/year.

Assuming the molecular clock, the age of each stratum $T$ can be calculated as $T = div/{\mu _{XY}}$, where the divergence between X and Y $div$ was inferred based on the pairwise X/Y lastZ alignments generated above. We extracted all alignments of each stratum, removed alignments that fell in coding regions or repetitive sequences identified by RepeatMasker and Tandem Repeats Finder (v4.09) [70], and concatenated them into a single sequence alignment. We only used X-Y3/Y5 alignment for the calculation of S0. Divergence was estimated with baseml in PAML package (v4.8) (RRID:SCR_014932) [74] under JC69 model, and the 95% confidence interval was estimated after 1,000 bootstraps. Divergence time of each stratum was calculated for each monotreme, and for the ancestral shared strata, we took the divergence time calculated from the larger alignment of the 2 monotremes in the main text.

### Ampliconic region analysis

We mainly followed Makova et al. [80] to identify the ampliconic region by 3 methods: lastZ, blastn, and sequencing depth. To detect palindrome (≥98% identity, arm length ≥8 Kb, spacer ≤500 Kb), we first performed lastZ alignment with parameter set “–self –format=general:name1,zstart1,end1,name2,strand2,zstart2+,end2+,id%,cigarx” and the palindrover obtained was used for palindrome detection. We further required the repeat content in the candidate palindrome to be <80%. Ampliconic region arranged in the array was detected with the BLASTN method. Basically, the X-linked (or Y-linked) sequences were repeat-masked and split into 5-Kb windows with 2-Kb overlaps. We BLASTNed the sequence to itself, and only alignments with >50% aligning rate and >99% identity were kept. We further merged the segments and required a merged length ≥10 Kb. We also considered depth information to identify ampliconic regions since the ampliconic regions might have collapsed during assembling. Briefly, we mapped male resequencing reads to the genome with BWA MEM and calculated the mean sequencing depth of each 5-Kb window after correcting with GC content with deepTools (v3.5.1) (RRID:SCR_016366) [81]. If the corrected sequencing depth of a nonPARX/Y window was larger than or equal to that of the autosomes, the window would be considered a candidate ampliconic region. We required the repeat content in the candidate ampliconic region identified by depth to be <80%. Ampliconic regions of the 3 methods were then merged with bedtools (v2.29.2) to obtain the final ampliconic region set. Genes with >80% of the length overlapping with the ampliconic regions were considered ampliconic genes. Olfactory receptor and vomeronasal receptor genes were excluded since they were found amplified in the whole genome and were not specifically sex-linked [9].

RNA sequencing data of platypus and echidna were obtained from NCBI with accession codes SRP000120, SRP102989, SRP233233, and SRP027593. Expression level as transcripts per million (TPM) was estimated with Kallisto (v0.46.1) [82] with parameter “–bias.” Expression was normalized with DESeq2 (v1.31.16) (RRID:SCR_015687) [83], and the gene expression tissue specificity was quantified as “tau” following the formula in Yanai et al. [84]. The expression profile of AGs in small nuclear RNA sequencing data of spermatogenesis was obtained from [47]. Human expression data were obtained from GTEx (RRID:SCR_013042), and the tissue specificity index tau was calculated with the same approach described above.

## Additional Files

**Supplementary Fig. S1**. Normalized depth distribution along example X, Y, and autosomal sequences.

**Supplementary Fig. S2**. Using Hi-C interaction strength (100 Kb resolution) between unplaced scaffold and anchored chromosomes to confirm which chromosome bears the scaffold. In echidna, higher interaction strength is observed between scaffold_101_arrow_ctg1 and X3, Y3, as well as between scaffold_145_arrow_ctg1 and Y3, X4, suggesting that scaffold_101_arrow_ctg1 and scaffold_145_arrow_ctg1 could be a X3Y3 PAR and Y3X4 PAR, respectively. Echidna scaffold_1_arrow_ctg1 has a higher interaction with both chr11 and chr27, and thus its exact origin chromosome is unknown. In platypus, scaffold_344_arrow_ctg1 has a higher interaction with X3, suggesting that this X-Div scaffold could be on X3.

**Supplementary Fig. S3**. Confirmation of an inversion artifact in the Illumina-based echidna assembly with Hi-C data under a 50-Kb resolution. Top heatmap: Hi-C map of the PacBio assembly; left heatmap: Hi-C map of the Illumina assembly. The SV breakpoints are highlighted with red dashed lines. Pairwise interaction strength in the four 200-Kb regions (a, b, c, and d) were extracted to confirm the SV. In the PacBio assembly, higher interaction was observed in a.vs.c than a.vs.d and b.vs.c; similarly, b.vs.d was higher than b.vs.c and a.vs.d. Both suggest that the genome structure (c-a-b-d) in the PacBio assembly is correct. In the Illumina-based assembly, a.vs.c > b.vs.c and b.vs.d > a.vs.d, but the order is c-b-a-d, suggesting that this genome structure in the Illumina assembly is wrong.

**Supplementary Fig. S4**. Improvement of the new echidna assembly, evaluated in contig length distribution (a), number of misassembly artifacts (b), BUSCO (c), and the number of bases (gap excluded) in sex chromosomes (d).

**Supplementary Fig. S5**. Confirmation of interchromosomal genomic rearrangement between platypus and echidna with Hi-C data under a 500-Kb resolution. Top heatmap: platypus; left heatmap: echidna. Coordinates of assembled telomeres are marked with black triangles.

**Supplementary Fig. S6**. Confirmation of interchromosomal genomic rearrangement between platypus and echidna with Hi-C data under a 500-Kb resolution. Top heatmap: platypus; left heatmap: echidna. Coordinates of assembled telomeres are marked with black triangles.

**Supplementary Fig. S7**. Confirmation of intrachromosomal genomic rearrangement between platypus and echidna with Hi-C data under a 500-Kb resolution. Top heatmap: platypus; left heatmap: echidna. Coordinates of assembled telomeres are marked with black triangles. Intrachromosomal SV breakpoints are highlighted with red dashed lines. Pairwise interaction strength in the four 5-Mb regions (a, b, c, and d) were extracted to confirm the SVs.

**Supplementary Fig. S8**. Ancestral karyotype reconstruction under a 300-Kb resolution.

**Supplementary Fig. S9**. Ancestral karyotype reconstruction under a 500-Kb resolution.

**Supplementary Fig. S10**. Mapping of mammalian ancestral chromosomes to chicken chromosomes. Chicken chromosomes are color-coded based on the homology of the mammalian ancestral chromosomes. Some of the microchromosomes have no color as they are unable to map to mammalian ancestral chromosomes.

**Supplementary Fig. S11**. *In silico* chromosome painting of mammalian ancestral karyotype (a) and orthologous chicken sequences (b) to each echidna and platypus sex chromosome. The recombination between PARs of X and Y is indicated by a bar (a) and crosses (b). We also labeled the genomic coordinates of the putative sex-determining gene *AMHX/Y*. Note that since the ancestral reconstruction is built based on the genome with only autosomes and Xs, we were not able to map ancestral chromosomes to the monotreme Y chromosomes.

**Supplementary Fig. S12**. Gametolog pair dS distribution for each stratum. (a) No siginficant difference can be found among the dS in S0_X1, S0_X2, S0_X3, and S0_X4 (note: platypus S0_X4 is on X5), suggesting that they may form ancestrally on a single chromome but then spread across the 4 ancestral Xs. (b) dS distribution for each stratum after merging S0_X1, S0_X2, S0_X3, and S0_X4 (or platypus S0_X5). Number in the brackets shows the number of gametolog pairs. The X chromosome locations are also noted for each stratum. Note that in echidna, only the X or Y gametolog is found in S3_E_ (Supplementary Table S15); therefore, no dS is available in the plot. We considered S1 and S2 as 2 different strata since they are located on 2 different chromosomes, and both are significantly different from S0; therefore, their recombination suppression was unlikely at the same time. Conclusion was drawn for S3P, S4P, and S5P for a similar reason.

**Supplementary Figure S13:** Alignment between echidna Y3 and platypus Y5.

**Supplementary Fig. S14**. Phylogeny of S1 (a), S2 (b), and S3_P_ (c) X/Y gametolog and the orthologs. (a) S4 gametolog pairs are clustered by sex chromosome instead of by species, and no gene conversion is detected, suggesting that S4 originated before species divergence. (b) While S2 gametolog pairs are clustered by species instead of by sex chromosomes, the bootstrap is low and gene conversion is detected between the X and Y of the same species, suggesting that S2 likely evolved ancestrally before species divergence. (c) S3_P_ gametolog pairs are clustered by species, but the orthologs in echidna locate on the autosome, and therefore, we consider the XY divergence happened independently in platypus. Red, X-linked gene; blue, Y-linked gene; black, autosomal gene. Bootstrap is noted at the internal nodes. Strong gene conversion signal (gene conversion ratio >10%) is marked by the link between genes. Pseudogenes are marked with a “p” suffix.

**Supplementary Fig. S15**. Phylogeny of S4_P_ (a), S5_P_, and S4_E_ (b) X/Y gametolog and the orthologs. Gametolog pairs are clustered by species instead of by sex chromosomes, and little gene conversion signal is detected, suggesting that these strata originated after species divergence. Red, X-linked gene; blue, Y-linked gene; black, autosomal gene; orange, PAR gene. Bootstrap is noted at the internal nodes. Strong gene conversion signal (gene conversion ratio >10%) is marked by link between genes.

**Supplementary Fig. S16**. Normalized male and female sequencing depth in echidna chrX5, scaffold_344_arrow_ctg1 and platypus chrX4, scaffold_1_arrow_ctg1. Red: female; blue: male; green: female-vs.-male depth ratio (f/m). PAR and nonPARX on the assembled echidna X5 and platypus X4 are marked in the plot. The female-vs.-male depth ratio is around 2 in echidna scaffold_344_arrow_ctg1, and the normalized depth of female and male is around four and one, respectively, suggesting that the scaffold is a nonPARX but is collapsed during assembling.

**Supplementary Fig. S17**. Alignment of the species-specific X and the autosomal sequences in the other monotreme. (a) Alignment between echidna chrX5:12,000,000–16,101,208 and the homologous sequences in platypus chr12:46,200,000–49,200,000 and scaffold_344_arrow_ctg1:1–741,479. Black bar indicates PAR. Duplicated genes encoding olfactory receptor (*OR*), vomeronasal receptor 1 (*V1R*), and 2 (*V2R*) are shown in blue, red, and orange, respectively. X/Y identity is calculated in a 1-Kb window and color-coded. (b) Alignment between platypus chrX4:4,800,000–8,639,456 and the homologous sequences in echidna chr27:1–1,000,000 and scaffold_1_arrow_ctg1:2,300,000–6,386,367. Black bar indicates PAR. Duplicated genes encoding olfactory receptor (*OR*), vomeronasal receptor 1 (*V1R*), 2 (*V2R*), and lipocalin (*LCN*) are shown in blue, red, orange, and green, respectively. X/Y identity is calculated in a 1-Kb window and color-coded. (c) Alignment of the inversion and its upstream region in human, opossum, platypus, and echidna shows that the inversion happened in echidna. Alignment of the inversion region is highlighted in red. (d) Significant X/Y identity is found between the region closer to the PAR boundary (chr27) than the region distant to PAB (scaffold_1_arrow_ctg1). X/Y sequence identity is calculated in a 1-Kb window. Two-sided Wilcoxon rank-sum test was performed.

**Supplementary Fig. S18**. The expression profile of *TACR3* in human, echidna (X-linked), and platypus (autosomal). Human expression data are obtained from https://www.gtexportal.org/home/gene/TACR3.

**Supplementary Fig. S19**. Confirmation of the translocation in X3. (a) Confirmation of translocation between platypus X3 and echidna X3 with Hi-C data under a 500-Kb resolution. Pairwise interaction strength in the four ∼5-Mb regions (a, b, c, and d) was extracted to confirm the translocation. Normalized male and female sequencing depth is also shown to indicate the involvement of a PAR-nonPARX transition. Normalized male and female depth, as well as female-vs.-male depth ratio, is also plotted along the two X3. PAR and gaps are marked as black and blue rectangles, respectively. (b) Alignment of the translocation and its upstream region in human, opossum, platypus, and echidna. Alignment of the translocation region is highlighted in red. Visualized region includes human chr6:1,949,424–2,785,777, opossum chr3:345,825,794–346,965,677, platypus: chrX3:17,430,000–18,000,000, and echidna chrX3:4,913,378–16,395,920.

**Supplementary Fig. S20**. Gene phylogeny of *SYCP3*s and *SYCP3Y*s.

**Supplementary Fig. S21**. The expression profile of *SYCP3Y* in platypus snRNA-seq data. Different colors indicate different cell types: blue, spermatogonia (SG); green, spermatocytes (SC); orange, round spe rmatids (round_SD); pink, elongated spermatids (elongated SD); yellow, other somatic cells. Plots were obtained from https://apps.kaessmannlab.org/SpermEvol/. Note that different annotations are used in Murat et al. and this study, and therefore, gene IDs are different.

**Supplementary Table S1**. Statistics of the sequencing data used in echidna genome assembling.

**Supplementary Table S2**. Statistics of the monotreme assemblies.

**Supplementary Table S3**. Chromosome assignment in echidna.

**Supplementary Table S4**. Identified X-Div and Y-Div sequences in platypus and echidna. Scaffolds with ‘?’ marked in the “sex chromosome” column indicate that the information was inferred via Hi-C.

**Supplementary Table S5**. The mean of interaction strength between unplaced scaffold with sex chromosome or autosome.

**Supplementary Table S6**. Echidna sex chromosome sequence assigned percentage.

**Supplementary Table S7**. Mapping information of male-specific transcripts to the 2 echidna assemblies.

**Supplementary Table S8**. Confirmation of the assembly errors in GCA_015598185.1 by the comparison with GCF_015852505.1. Genomic coordinate is based on GCF_015852505.1. INV: inversion; TRANS: translocation; INVTR: inverted translocation; P: support to PacBio-based assembly; I: support to Illumina-based assembly.

**Supplementary Table S9**. Gap-filling state in the echidna assembly.

**Supplementary Table S10**. CAR-ordered information during ancestral karyotype reconstruction under a 300-Kb and 500-Kb resolution. CAR: continuous ancestral region.

**Supplementary Table S11**. Reconstructed ancestral karyotype information under a 300-Kb resolution.

**Supplementary Table S12**. Reconstructed ancestral karyotype information under a 500-Kb resolution.

**Supplementary Table S13**. *In silico* chromosome painting information between each monotreme and chicken.

**Supplementary Table S14**. The genomic coordinates of each stratum.

**Supplementary Table S15**. X/Y gametolog pairs in monotremes.

**Supplementary Table S16**. Statistics of X/Y gametolog pairs number in monotremes S0.

**Supplementary Table S17**. The statistic of XY alignment.

**Supplementary Table S18**. Summary of gene conversion between X and Y.

**Supplementary Table S19**. The divergence of each sex chromosome in echidna and platypus.

**Supplementary Table S20**. Expression matrix of sex-linked genes in platypus (units in TPM).

**Supplementary Table S21**. Expression matrix of sex-linked genes in echidna (units in TPM).

**Supplementary Table S22**. The enrichment test of testis-specific expression in ampliconic genes. Olfactory and vomeronasal receptor genes are excluded in the analysis.

**Supplementary Table S23**. Statistics of the number of testis-specific AGs and their ortholog expression profile in humans.

**Supplementary Table S24**. Autosome-derived Y and its autosomal homologs in monotremes.

giae112_Supplemental_Files

giae112_GIGA-D-24-00337_Original_Submission

giae112_GIGA-D-24-00337_Revision_1

giae112_Response_to_Reviewer_Comments_Original_Submission

giae112_Reviewer_1_Report_Original_Submission Thomas Liehr -- 9/30/2024

giae112_Reviewer_2_Report_Original_Submission Dmitry A Filatov --10/4/2024

giae112_Reviewer_3_Report_Original_Submission Vladimir Trifonov --10/10/2024

## Abbreviations

AG: ampliconic gene; BUSCO: Benchmarking Universal Single-Copy Orthologs; MON: monotreme ancestral chromosome; MRCA: most recent common ancestor; PAR: pseudoautosomal region; SDR: sex-differentiated region.

## Data Availability

The genomic data generated in this study have been submitted to the NCBI BioProject database under accession number PRJNA1191144. The genome assembly is available at NCBI under BioProject PRJNA607237. All additional supporting data are available in the *GigaScience* repository, GigaDB [85].

## References

[1] Damas J, Corbo M, Lewin H. Vertebrate chromosome evolution. Annu Rev Anim Biosci. 2021;9:1–27. 10.1146/annurev-animal-020518-114924. 33186504

[2] Ferguson-Smith MA, Trifonov V. Mammalian karyotype evolution. Nat Rev Genet. 2007;8(12):950–62. 10.1038/nrg2199. 18007651

[3] Guerrero RF, Kirkpatrick M. Local adaptation and the evolution of chromosome fusions. Evolution. 2014;68(10):2747–56. 10.1111/evo.12481. 24964074

[4] Rieseberg L . Box 1. Chromosomal rearrangements and meiosis. Trends Ecol Evol. 2001;7(16):351–58. 10.1016/s0169-5347(01)02187-5 11403867

[5] Yin Y, Fan H, Zhou B, et al. Molecular mechanisms and topological consequences of drastic chromosomal rearrangements of Muntjac deer. Nat Commun. 2021;12(1):6858. 10.1038/s41467-021-27091-0. 34824214 PMC8617201

[6] Dussex N, Van Der Valk T, Morales HE, et al. Population genomics of the critically endangered kākāpō. Cell Genomics. 2021;1(1):100002 10.1016/j.xgen.2021.100002. 36777713 PMC9903828

[7] Jebb D, Huang Z, Pippel M, et al. Six reference-quality genomes reveal evolution of bat adaptations. Nature. 2020;583(7817):578–84. 10.1038/s41586-020-2486-3. 32699395 PMC8075899

[8] Rhie A, McCarthy SA, Fedrigo O, et al. Towards complete and error-free genome assemblies of all vertebrate species. Nature. 2021;592(7856):737–46. 10.1038/s41586-021-03451-0. 33911273 PMC8081667

[9] Zhou Y, Shearwin-Whyatt L, Li J, et al. Platypus and echidna genomes reveal mammalian biology and evolution. Nature. 2021;592(7856):756–62. 10.1038/s41586-020-03039-0. 33408411 PMC8081666

[10] Cortez D, Marin R, Toledo-Flores D, et al. Origins and functional evolution of Y chromosomes across mammals. Nature. 2014;508(7497):488–93. 10.1038/nature13151. 24759410

[11] Warren WC, Hillier LDW, A. J, et al. Genome analysis of the platypus reveals unique signatures of evolution. Nature. 2008;453(7192):175–83. 10.1038/nature06936. 18464734 PMC2803040

[12] Deakin J, Graves J, Rens WJC, et al. The evolution of marsupial and monotreme chromosomes. Cytogenet Genome Res. 2012;137(2–4):113–29. 10.1159/000339433. 22777195

[13] McMillan D, Miethke P, Alsop AE, et al. Characterizing the chromosomes of the platypus (Ornithorhynchus anatinus). Chromosome Res. 2007;15:961–74. 10.1007/s10577-007-1186-2. 18185982

[14] Ruiz-Herrera A, Farré M, Robinson T. Molecular cytogenetic and genomic insights into chromosomal evolution. Heredity. 2012;108(1):28–36. 10.1038/hdy.2011.102. 22108627 PMC3238115

[15] Zhou Q, Zhang J, Bachtrog D, et al. Complex evolutionary trajectories of sex chromosomes across bird taxa. Science. 2014;346(6215):1246338. 10.1126/science.1246338. 25504727 PMC6445272

[16] Lahn BT, Page DC. Four evolutionary strata on the human X chromosome. Science. 1999;286(5441):964–67. 10.1126/science.286.5441.964. 10542153

[17] Kitano J, Ross JA, Mori S, et al. A role for a neo-sex chromosome in stickleback speciation. Nature. 2009;461(7267):1079–83. 10.1038/nature08441. 19783981 PMC2776091

[18] Charlesworth D, Charlesworth B. Sex differences in fitness and selection for centric fusions between sex-chromosomes and autosomes. Genet Res. 1980;35(2):205–14. 10.1017/S0016672300014051. 6930353

[19] Ashley T . X-autosome translocations, meiotic synapsis, chromosome evolution and speciation. Cytogenet Genome Res. 2002;96(1–4):33–39. 10.1159/000063030. 12438777

[20] Eicher EM . X-autosome translocations in the mouse: total inactivation versus partial inactivation of the X chromosome. Adv Genet. 1970;15:175–259. 10.1016/S0065-2660(08)60074-7. 4936423

[21] Grützner F, Rens W, Tsend-Ayush E, et al. In the platypus a meiotic chain of ten sex chromosomes shares genes with the bird Z and mammal X chromosomes. Nature. 2004;432(7019):913–17. 10.1038/nature03021. 15502814

[22] Rens W, Grützner F, O'brien PC, et al. Resolution and evolution of the duck-billed platypus karyotype with an X1Y1×2Y2×3Y3×4Y4×5Y5 male sex chromosome constitution. Proc Natl Acad Sci USA. 2004;101(46):16257–61. 10.1073/pnas.0405702101. 15534209 PMC528943

[23] Rens W, O'Brien PC, Grützner F, et al. The multiple sex chromosomes of platypus and echidna are not completely identical and several share homology with the avian Z. Genome Biol. 2007;8:1–21. 10.1186/gb-2007-8-11-r243. PMC2258203 18021405

[24] Gruetzner F, Ashley T, Rowell DM, et al. How did the platypus get its sex chromosome chain? A comparison of meiotic multiples and sex chromosomes in plants and animals. Chromosoma. 2006;115:75–88. 10.1007/s00412-005-0034-4. 16344965

[25] Dohm JC, Tsend-Ayush E, Reinhardt R, et al. Disruption and pseudoautosomal localization of the major histocompatibility complex in monotremes. Genome Biol. 2007;8:1–16. 10.1186/gb-2007-8-8-r175. PMC2375005 17727704

[26] Wrigley JM, Graves J. Karyotypic conservation in the mammalian order Monotremata (subclass Prototheria). Chromosoma. 1988;96(3):231–47. 10.1007/BF00302363. 3359880

[27] Deakin JE, Delbridge ML, Koina E, et al. Reconstruction of the ancestral marsupial karyotype from comparative gene maps. BMC Evol Biol. 2013;13(1):1–15. 10.1186/1471-2148-13-258. 24261750 PMC4222502

[28] Froenicke L . Origins of primate chromosomes—as delineated by Zoo-FISH and alignments of human and mouse draft genome sequences. Cytogenet Genome Res. 2005;108(1–3):122–38. 10.1159/000080810. 15545724

[29] Kim J, Farré M, Auvil L, et al. Reconstruction and evolutionary history of eutherian chromosomes. Proc Natl Acad Sci USA. 2017;114(27):E5379–E88. 10.1073/pnas.1702012114. 28630326 PMC5502614

[30] Uno Y, Nishida C, Tarui H, et al. Inference of the protokaryotypes of amniotes and tetrapods and the evolutionary processes of microchromosomes from comparative gene mapping. PLoS One. 2012;7(12):e53027. 10.1371/journal.pone.0053027. 23300852 PMC3534110

[31] Waters PD, Patel HR, Ruiz-Herrera A, et al. Microchromosomes are building blocks of bird, reptile, and mammal chromosomes. Proc Natl Acad Sci USA. 2021;118(45):e2112494118. 10.1073/pnas.2112494118. 34725164 PMC8609325

[32] Marais G, Galtier N. Sex chromosomes: how XY recombination stops. Curr Biol. 2003;13(16):R641–R43. 10.1016/S0960-9822(03)00570-0. 12932341

[33] Topaloglu AK, Reimann F, Guclu M, et al. TAC3 and TACR3 mutations in familial hypogonadotropic hypogonadism reveal a key role for Neurokinin B in the central control of reproduction. Nat Genet. 2009;41(3):354–58. 10.1038/ng.306. 19079066 PMC4312696

[34] Zhou Q, Bachtrog D. Sex-specific adaptation drives early sex chromosome evolution in Drosophila. Science. 2012;337(6092):341–45. 10.1126/science.1225385. 22822149 PMC4107656

[35] Hughes JF, Page DC. The biology and evolution of mammalian Y chromosomes. Annu Rev Genet. 2015;49:507–27. 10.1146/annurev-genet-112414-055311. 26442847

[36] Miga KH, Koren S, Rhie A, et al. Telomere-to-telomere assembly of a complete human X chromosome. Nature. 2020;585(7823):79–84. 10.1038/s41586-020-2547-7. 32663838 PMC7484160

[37] Rhie A, Nurk S, Cechova M, et al. The complete sequence of a human Y chromosome. Nature. 2023;621:1–11. 10.1038/s41586-023-06457-y PMC10752217 37612512

[38] Skaletsky H, Kuroda-Kawaguchi T, Minx PJ, et al. The male-specific region of the human Y chromosome is a mosaic of discrete sequence classes. Nature. 2003;423(6942):825–37. 10.1038/nature01722. 12815422

[39] Bellott DW, Skaletsky H, Pyntikova T, et al. Convergent evolution of chicken Z and human X chromosomes by expansion and gene acquisition. Nature. 2010;466(7306):612–16. 10.1038/nature09172. 20622855 PMC2943333

[40] Mueller JL, Skaletsky H, Brown LG, et al. Independent specialization of the human and mouse X chromosomes for the male germ line. Nat Genet. 2013;45(9):1083–87. 10.1038/ng.2705. 23872635 PMC3758364

[41] Ross MT, Grafham DV, Coffey AJ, et al. The DNA sequence of the human X chromosome. Nature. 2005;434(7031):325–37. 10.1038/nature03440. 15772651 PMC2665286

[42] Soh YS, Alföldi J, Pyntikova T, et al. Sequencing the mouse Y chromosome reveals convergent gene acquisition and amplification on both sex chromosomes. Cell. 2014;159(4):800–13. 10.1016/j.cell.2014.09.052. 25417157 PMC4260969

[43] Bachtrog D, Mahajan S, Bracewell R. Massive gene amplification on a recently formed Drosophila Y chromosome. Nat Ecol Evol. 2019;3(11):1587–97. 10.1038/s41559-019-1009-9. 31666742 PMC7217032

[44] Bhowmick BK, Satta Y, Takahata N. The origin and evolution of human ampliconic gene families and ampliconic structure. Genome Res. 2007;17(4):441–50. 10.1101/gr.5734907. 17185645 PMC1832091

[45] Yuan L, Pelttari J, Brundell E, et al. The synaptonemal complex protein SCP3 can form multistranded, cross-striated fibers in vivo. J Cell Biol. 1998;142(2):331–39. 10.1083/jcb.142.2.331. 9679134 PMC2133048

[46] Casey AE, Daish TJ, Grutzner F. Identification and characterisation of synaptonemal complex genes in monotremes. Gene. 2015;567(2):146–53. 10.1016/j.gene.2015.04.089. 25981592

[47] Murat F, Mbengue N, Winge SB, et al. The molecular evolution of spermatogenesis across mammals. Nature. 2023;613(7943):308–16. 10.1038/s41586-022-05547-7. 36544022 PMC9834047

[48] Page SL, Hawley R. The genetics and molecular biology of the synaptonemal complex. Annu Rev Cell Dev Biol. 2004;20:525–58. 10.1146/annurev.cellbio.19.111301.155141. 15473851

[49] Tsend-Ayush E, Kortschak RD, Bernard P, et al. Identification of mediator complex 26 (Crsp7) gametologs on platypus X1 and Y5 sex chromosomes: a candidate testis-determining gene in monotremes?. Chromosome Res. 2012;20:127–38. 10.1007/s10577-011-9270-z. 22215486

[50] Blackmon H, Ross L, Bachtrog D. Sex determination, sex chromosomes, and karyotype evolution in insects. JHERED. 2017;108(1):78–93. 10.1093/jhered/esw047. PMC6281344 27543823

[51] Gunski RJ, Cañedo AD, Garnero ADV, et al. Multiple sex chromosome system in penguins (Pygoscelis, Spheniscidae). CCG. 2017;11(3):541. 10.3897/CompCytogen.v11i3.13795. PMC5646662 29093802

[52] Miura I, Shams F, Lin S-M, et al. Evolution of a multiple sex-chromosome system by three-sequential translocations among potential sex-chromosomes in the Taiwanese frog Odorrana swinhoana. Cells. 2021;10(3):661. 10.3390/cells10030661. 33809726 PMC8002213

[53] Daish T, Casey A, Grützner F. Platypus chain reaction: directional and ordered meiotic pairing of the multiple sex chromosome chain in Ornithorhynchus anatinus. Reprod Fertil Dev. 2009;21(8):976–84. 10.1071/RD09085. 19874721

[54] Casey AE, Daish TJ, Barbero JL, et al. Differential cohesin loading marks paired and unpaired regions of platypus sex chromosomes at prophase I. Sci Rep. 2017;7(1):4217. 10.1038/s41598-017-04560-5. 28652620 PMC5484699

[55] Manni M, Berkeley MR, Seppey M, et al. BUSCO update: novel and streamlined workflows along with broader and deeper phylogenetic coverage for scoring of eukaryotic, prokaryotic, and viral genomes. Mol Biol Evol. 2021;38(10):4647–54. 10.1093/molbev/msab199. 34320186 PMC8476166

[56] Huang N, Li H. compleasm: a faster and more accurate reimplementation of BUSCO. Bioinformatics. 2023;39(10):btad595. 10.1093/bioinformatics/btad595. 37758247 PMC10558035

[57] Li H, Durbin R. Fast and accurate short read alignment with Burrows–Wheeler transform. Bioinformatics. 2009;25(14):1754–60. 10.1093/bioinformatics/btp324. 19451168 PMC2705234

[58] Danecek P, Bonfield JK, Liddle J, et al. Twelve years of SAMtools and BCFtools. *Gigascience*. 2021;10(2):giab008. 10.1093/gigascience/giab008. 33590861 PMC7931819

[59] Quinlan AR, Hall I. BEDTools: a flexible suite of utilities for comparing genomic features. Bioinformatics. 2010;26(6):841–42. 10.1093/bioinformatics/btq033. 20110278 PMC2832824

[60] Yang C, Zhou Y, Marcus S, et al. Evolutionary and biomedical insights from a marmoset diploid genome assembly. Nature. 2021;594(7862):227–33. 10.1038/s41586-021-03535-x. 33910227 PMC8189906

[61] Ramírez F, Bhardwaj V, Arrigoni L, et al. High-resolution TADs reveal DNA sequences underlying genome organization in flies. Nat Commun. 2018;9(1):189. 10.1038/s41467-017-02525-w. 29335486 PMC5768762

[62] Kent WJ . BLAT—the BLAST-like alignment tool. Genome Res. 2002;12(4):656–64. 10.1101/gr.229202. 11932250 PMC187518

[63] Bickhart DM, Rosen BD, Koren S, et al. Single-molecule sequencing and chromatin conformation capture enable de novo reference assembly of the domestic goat genome. Nat Genet. 2017;49(4):643–50. 10.1038/ng.3802. 28263316 PMC5909822

[64] Dudchenko O, Batra SS, Omer AD, et al. De novo assembly of the Aedes aegypti genome using Hi-C yields chromosome-length scaffolds. Science. 2017;356(6333):92–95. 10.1126/science.aal3327. 28336562 PMC5635820

[65] Harris RS . Improved pairwise alignment of genomic DNA. PhD thesis. Philadelphia: The Pennsylvania State University; 2007. https://www.bx.psu.edu/~rsharris/rsharris_phd_thesis_2007.pdf.

[66] Kumar S, Stecher G, Suleski M, et al. TimeTree: a resource for timelines, timetrees, and divergence times. Mol Biol Evol. 2017;34(7):1812–19. 10.1093/molbev/msx116. 28387841

[67] Jones BR, Rajaraman A, Tannier E, et al. ANGES: reconstructing ANcestral GEnomeS maps. Bioinformatics. 2012;28(18):2388–90. 10.1093/bioinformatics/bts457. 22820205

[68] Wang Y, Tang H, DeBarry JD, et al. MCScanX: a toolkit for detection and evolutionary analysis of gene synteny and collinearity. Nucleic Acids Res. 2012;40(7):e49. 10.1093/nar/gkr1293. 22217600 PMC3326336

[69] Tesler G . GRIMM: genome rearrangements web server. Bioinformatics. 2002;18(3):492–93. 10.1093/bioinformatics/18.3.492. 11934753

[70] Benson G . Tandem repeats finder: a program to analyze DNA sequences. Nucleic Acids Res. 1999;27(2):573–80. 10.1093/nar/27.2.573. 9862982 PMC148217

[71] Smit A, Hubley R, Green P. RepeatMasker Open-4.0. http://www.repeatmasker.org. Accessed 1 December 2022.

[72] Krzywinski M, Schein J, Birol I, et al. Circos: an information aesthetic for comparative genomics. Genome Res. 2009;19(9):1639–45. 10.1101/gr.092759.109. 19541911 PMC2752132

[73] Löytynoja AJ . Phylogeny-aware alignment with PRANK. Methods Mol Biol. 2014;1079:155–70. 10.1007/978-1-62703-646-7_10. 24170401

[74] Yang Z . PAML 4: phylogenetic analysis by maximum likelihood. Mol Biol Evol. 2007;24(8):1586–91. 10.1093/molbev/msm088. 17483113

[75] Stamatakis AJB . RAxML version 8: a tool for phylogenetic analysis and post-analysis of large phylogenies. Bioinformatics. 2014;30(9):1312–13. 10.1093/bioinformatics/btu033. 24451623 PMC3998144

[76] Sawyer S . Statistical tests for detecting gene conversion. Mol Biol Evol. 1989;65:526–38. 10.1093/oxfordjournals.molbev.a040567. 2677599

[77] Lopez-Delisle L, Rabbani L, Wolff J, et al. pyGenomeTracks: reproducible plots for multivariate genomic datasets. Bioinformatics. 2021;37(3):422–23. 10.1093/bioinformatics/btaa692. 32745185 PMC8058774

[78] Martin HC, Batty EM, Hussin J, et al. Insights into platypus population structure and history from whole-genome sequencing. Mol Biol Evol. 2018;35(5):1238–52. 10.1093/molbev/msy041. 29688544 PMC5913675

[79] Link V, Aguilar-Gómez D, Ramírez-Suástegui C, et al. Male mutation bias is the main force shaping chromosomal substitution rates in monotreme mammals. Genome Biol Evolut. 2017;9(9):2198–210. 10.1093/gbe/evx155. PMC5604096 28922870

[80] Makova KD, Pickett BD, Harris RS, et al. The complete sequence and comparative analysis of ape sex chromosomes. Nature. 2024;630:1–11. 10.1038/s41586-024-07473-2 PMC11168930 38811727

[81] Ramírez F, Ryan DP, Grüning B, et al. deepTools2: a next generation web server for deep-sequencing data analysis. Nucleic Acids Res. 2016;44:W160–W65. 10.1093/nar/gkw257. 27079975 PMC4987876

[82] Bray NL, Pimentel H, Melsted P, et al. Near-optimal probabilistic RNA-seq quantification. Nat Biotechnol. 2016;34(5):525–27. 10.1038/nbt.3519. 27043002

[83] Love MI, Huber W, Anders S. Moderated estimation of fold change and dispersion for RNA-seq data with DESeq2. Genome Biol. 2014;15(12):1–21. 10.1186/s13059-014-0550-8. PMC4302049 25516281

[84] Yanai I, Benjamin H, Shmoish M, et al. Genome-wide midrange transcription profiles reveal expression level relationships in human tissue specification. Bioinformatics. 2005;21(5):650–59. 10.1093/bioinformatics/bti042. 15388519

[85] Zhou Y, Jin J, Li X, et al. Supporting data for “Chromosome-Level Echidna Genome Illuminates Evolution of Multiple Sex Chromosome System in Monotremes.”. *GigaScience Database* 2024. 10.5524/102609. PMC11710854 39778707

