# Supplementary material for: Chromosome-level echidna genome illuminates evolution of multiple sex chromosome system in monotremes
Source: Gigascience. 2025 Jan 9;14:giae112. doi: 10.1093/gigascience/giae112 (PMC11710854; doi:10.1093/gigascience/giae112)
Supplement: giae112_Supplemental_Files [file giae112_supplemental_files.zip › Supplemental_Text_Figures.final.241014.docx]

Supplemental Text

# Comparison between the echidna assemblies

The two echidna assemblies were aligned by MUMmer (v3.23) (Kurtz, et al. 2004) with parameters set “-maxmatch -c 500 -b 500 -l000. The alignment result was filtered by ‘delta-filter’ with parameters “-m -i 90 -l 100”. We used Syri (v1.5) (Goel, et al. 2019) to find structural variants (SVs) between the two assemblies with default parameters. Only SVs (including inversions, translocations and inverted translocations) larger than 100 Kb were kept as those were likely to be misassembly artifacts. The SVs and the flanking region were further visualized for manual examination to confirm the identified SVs. We defined the 5’ and 3’ end of the SV from the PacBio assembly as a and b, and the syntenic region upstream and downstream the SV from PacBio assembly as c and d (example shown in **Supplemental Notes Supplementary fig. S1**).


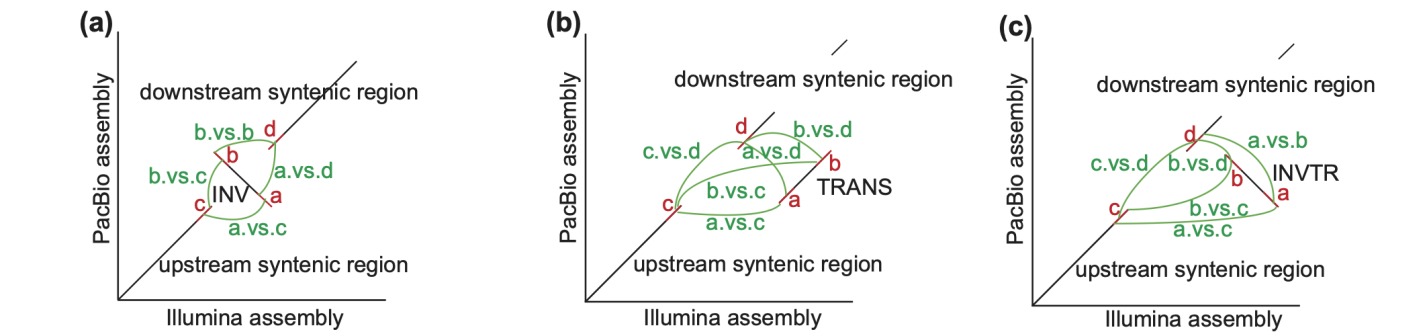


**Supplemental Notes Supplementary fig. S1**. Examples of SV shown from left to right: inversion (INV in a), translocation (TRANS in b) and inverted translocation (INVTR in c). a, b, c and d in red are the regions used for validation: a, b: the 5’ and 3’ end of the SV; c, the 3’ end of the upstream syntenic region; d, the 5’ end of the downstream syntenic region.

Each structural variant was checked with PacBio long reads, 10X-linked reads and Hi-C. PacBio reads were mapped to the PacBio assembly with pbmm2 (v1.4.0). 10X-linked reads were first processed by trimming the 16 bp barcode in read1, and mapped to PacBio assembly with BWA MEM. Hi-C reads were mapped to PacBio assembly with hicpro (v2.10.0) (Servant, et al. 2015). Interaction matrices under 10 Kb, 50 Kb, 100 Kb, 500 Kb were generated and normalized with the ICED method. The matrices were converted into h5 format and visualized with hicexplorer package (v3.7.2).

SV confirmation was performed in the following criteria:

- For PacBio long reads, we examined if PacBio long reads were able to cross c-a and b-d. At least 10 bp overlap between long reads was required.
- For 10X-linked reads, we extracted 10 Kb region from a, b, c, d and counted the shared number of barcodes of each region pair, i.e., a.v.s.c, b.vs.c, a.vs.d and b.vs.d.
- For Hi-C reads, we extracted 100 Kb region from a, b, c, d, and examined the ICED-normalized interaction strength under 50 Kb resolution of each region pair.

For inversion,

- If a.vs.c > a.vs.d and b.vs.d > b.vs.c, the PacBio assembly was correct.
- If a.vs.d < a.vs.c and b.vs.d < b.vs.c, the Illumia assembly was correct

For translocation,

- If a.vs.c > a.vs.d and b.vs.d > b.vs.c, the PacBio assembly was correct.
- If c.vs.d > a.vs.c and c.vs.d > b.vs.d, the Illumia assembly was correct.

# Analysis of the platypus specific sex chromosome

The available chromosome-scale echidna assembly also enables us to study the species-specific change on platypus specific X, the chrX4. On this X chromosome, an ~3.64 Mb region harboring 28 protein-coding genes is evolved into X-Div in the assembled 8.64 Mb sequence (**supplementary supplementary fig. S17c, supplementary supplementary fig. S18b**). Different from the situation of echidna X5, X-Div on platypus X4 is homologous to echidna chr27 and an unplaced scaffold (scaffold_1_arrow_ctg1), both of which share similar male and female sequencing depth thus platypus X4 originated after platypus diverged from echidna (**supplementary supplementary fig. S17d**). X/Y sequence identity was significantly higher at the region close to the PAR boundary (homologous to echidna chr27) than the region distant to PAB (homologous to echidna scaffold_1_arrow_ctg1) (**supplementary supplementary fig. S18d**, one-sided Wilcoxon rank-sum test, *p* < 0.01). Thus, we suggested that, unlike the trajectory of echidna X5, platypus X4 only experienced a series of recombination suppression of the proto sex chromosome, starting from the region distant to the current PAR.

# Reference

Goel M, Sun H, Jiao W-B, Schneeberger KJGb. 2019. SyRI: finding genomic rearrangements and local sequence differences from whole-genome assemblies. Genome biology 20:1-13.

Kurtz S, Phillippy A, Delcher AL, Smoot M, Shumway M, Antonescu C, Salzberg SLJGb. 2004. Versatile and open software for comparing large genomes. Genome biology 5:1-9.

Servant N, Varoquaux N, Lajoie BR, Viara E, Chen C-J, Vert J-P, Heard E, Dekker J, Barillot EJGb. 2015. HiC-Pro: an optimized and flexible pipeline for Hi-C data processing. Genome biology 16:1-11.

Supplemental Figures


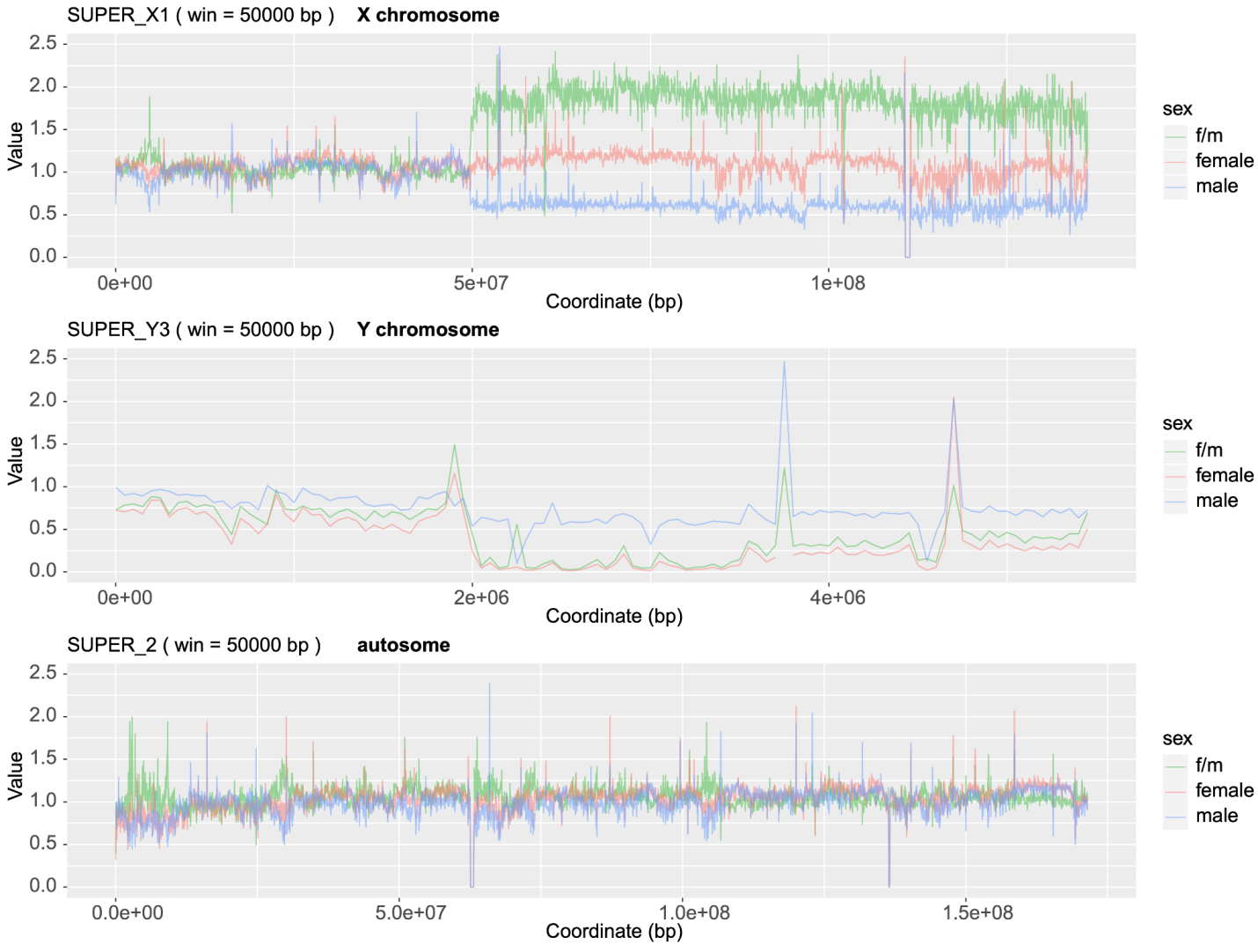


**Supplementary fig. S1. Normalized depth distribution along example X, Y and autosomal sequences.**


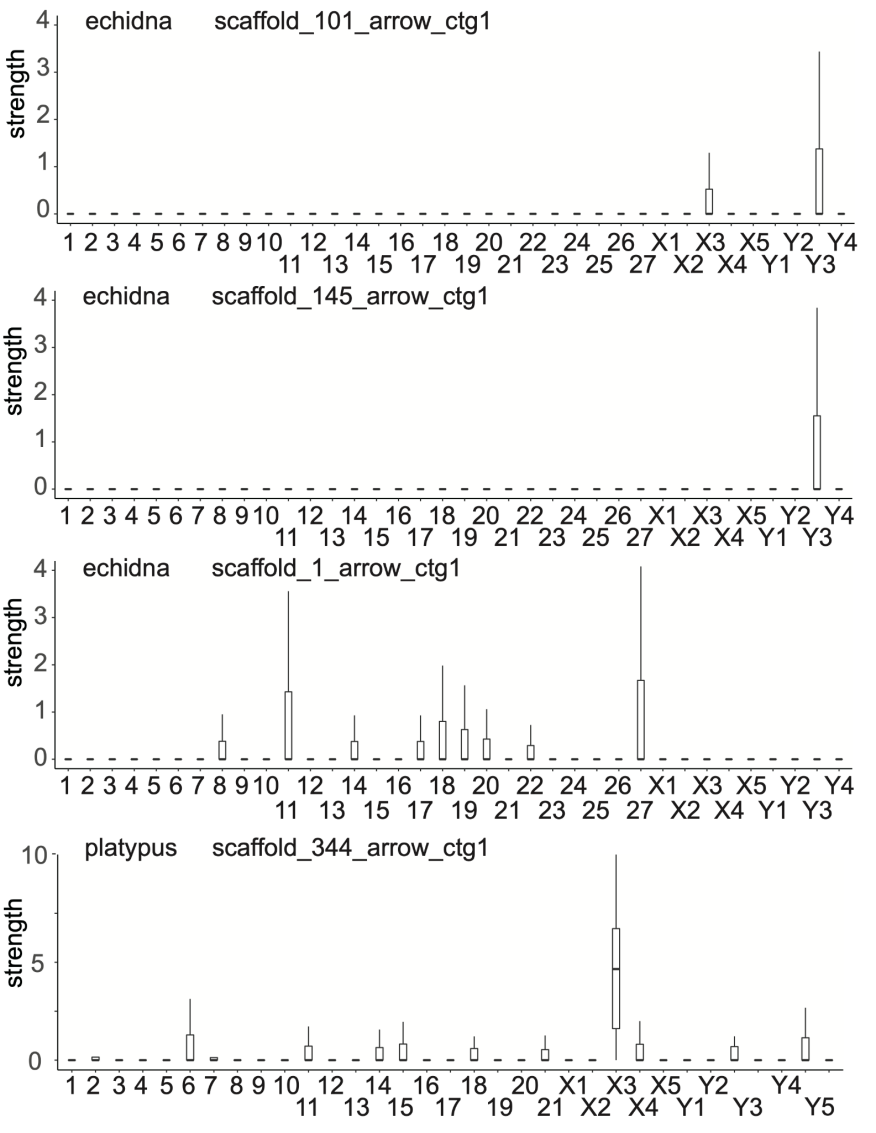


**Supplementary fig. S2. Using Hi-C interaction strength (100 Kb resolution) between unplaced scaffold and anchored chromosomes to confirm which chromosome bears the scaffold.** In echidna, higher interaction strength is observed between scaffold_101_arrow_ctg1 and X3, Y3, between scaffold_145_arrow_ctg1 and Y3, X4, suggesting that scaffold_101_arrow_ctg1 and scaffold_145_arrow_ctg1 could be a X3Y3 PAR and Y3X4 PAR, respectively. Echidna scaffold_1_arrow_ctg1 has higher interaction to both chr11 and chr27, thus its exact origin chromosome is unknown. In platypus scaffold_344_arrow_ctg1 has higher interaction to X3, suggesting that this X-Div scaffold could be on X3.


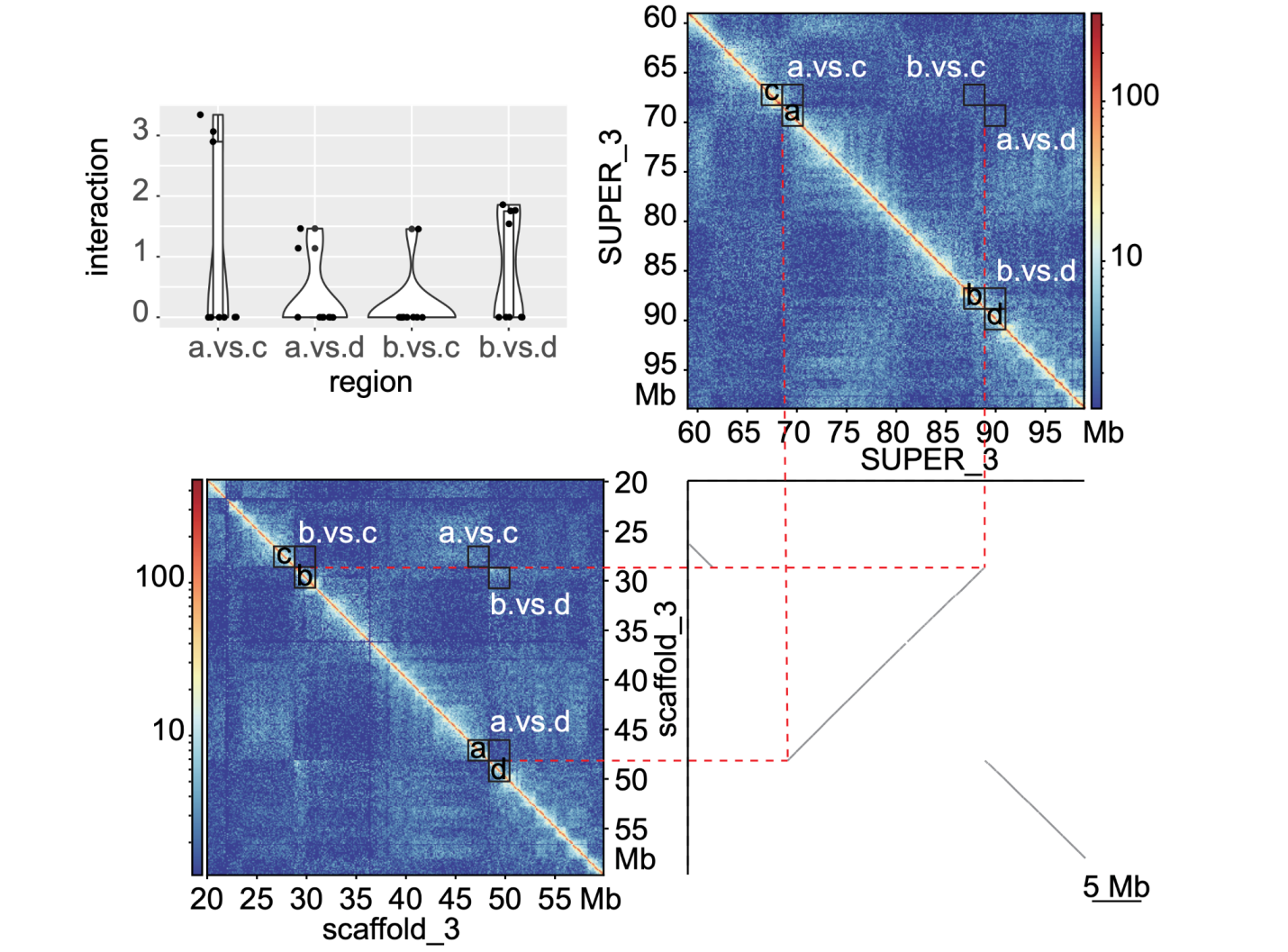


**Supplementary fig. S3. Confirmation of an inversion artifact in the Illumina-based echidna assembly with Hi-C data under 50 Kb resolution.** Top heatmap: Hi-C map of the PacBio assembly, left heatmap: Hi-C map of the Illumina assembly. The SV breakpoints are highlighted with red dash lines. Pairwise interaction strength in the four 200 Kb regions (a, b, c and d) were extracted to confirm the SV. In the PacBio assembly, higher interaction was observed in a.vs.c than a.vs.d and b.vs.c; similarly, b.vs.d was higher than b.vs.c and a.vs.d. Both suggest that the genome structure (c-a-b-d) in PacBio assembly is correct. In the Illumina-based assembly, a.vs.c > b.vs.c, and b.vs.d > a.vs.d, but the order is c-b-a-d, suggesting that this genome structure in the Illumina assembly is wrong.

**Supplementary fig. S4. Improvement of the new echidna assembly, evaluated in contig length distribution (a), number of mis-assembly artifacts (b), BUSCO (c) and the number of bases (gap excluded) in sex chromosomes (d).**


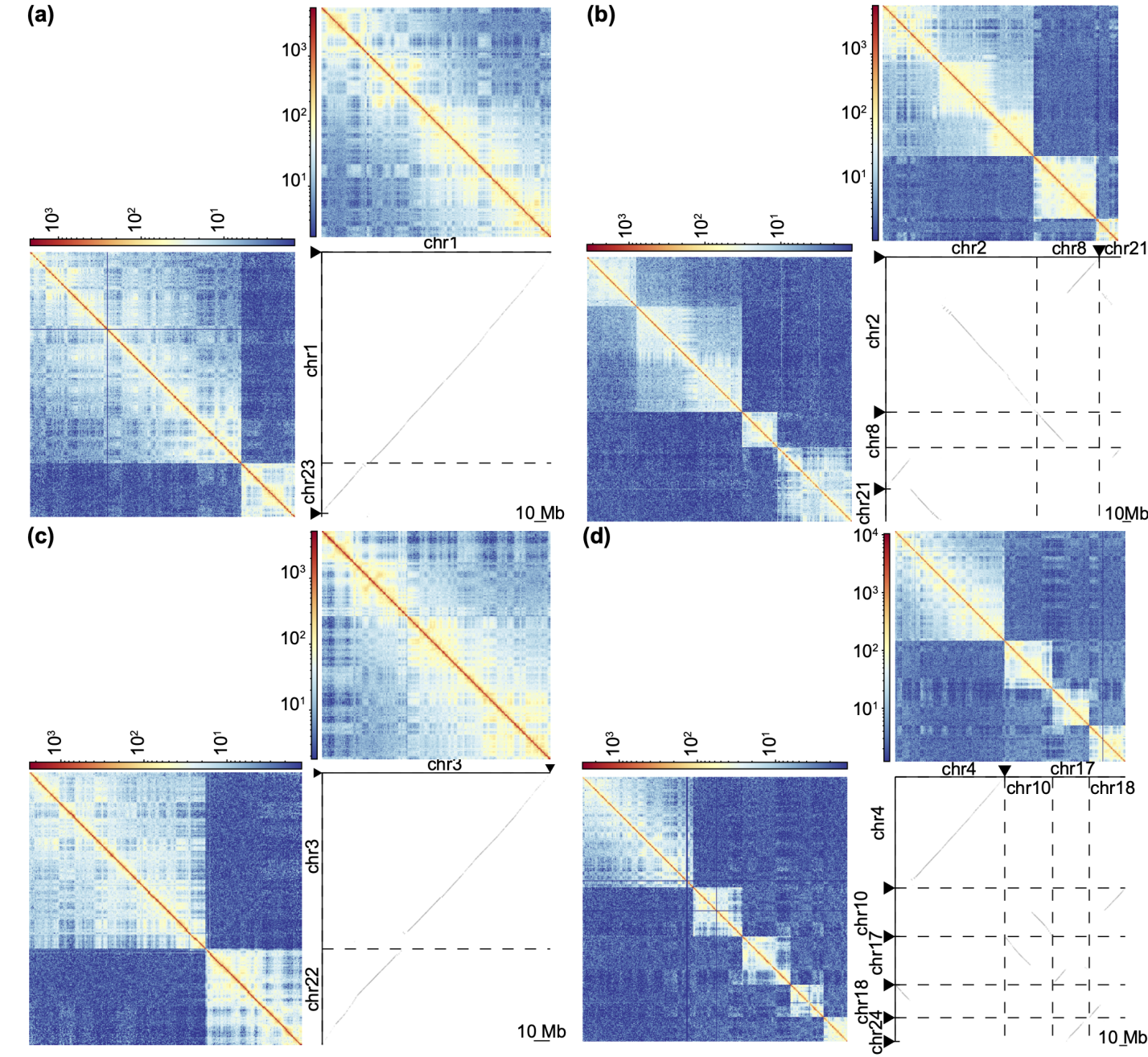


**Supplementary fig. S5. Confirmation of interchromosomal genomic rearrangement between platypus and echidna with Hi-C data under 500 Kb resolution.** Top heatmap: platypus, left heatmap: echidna. Coordinates of assembled telomeres are marked with black triangles.


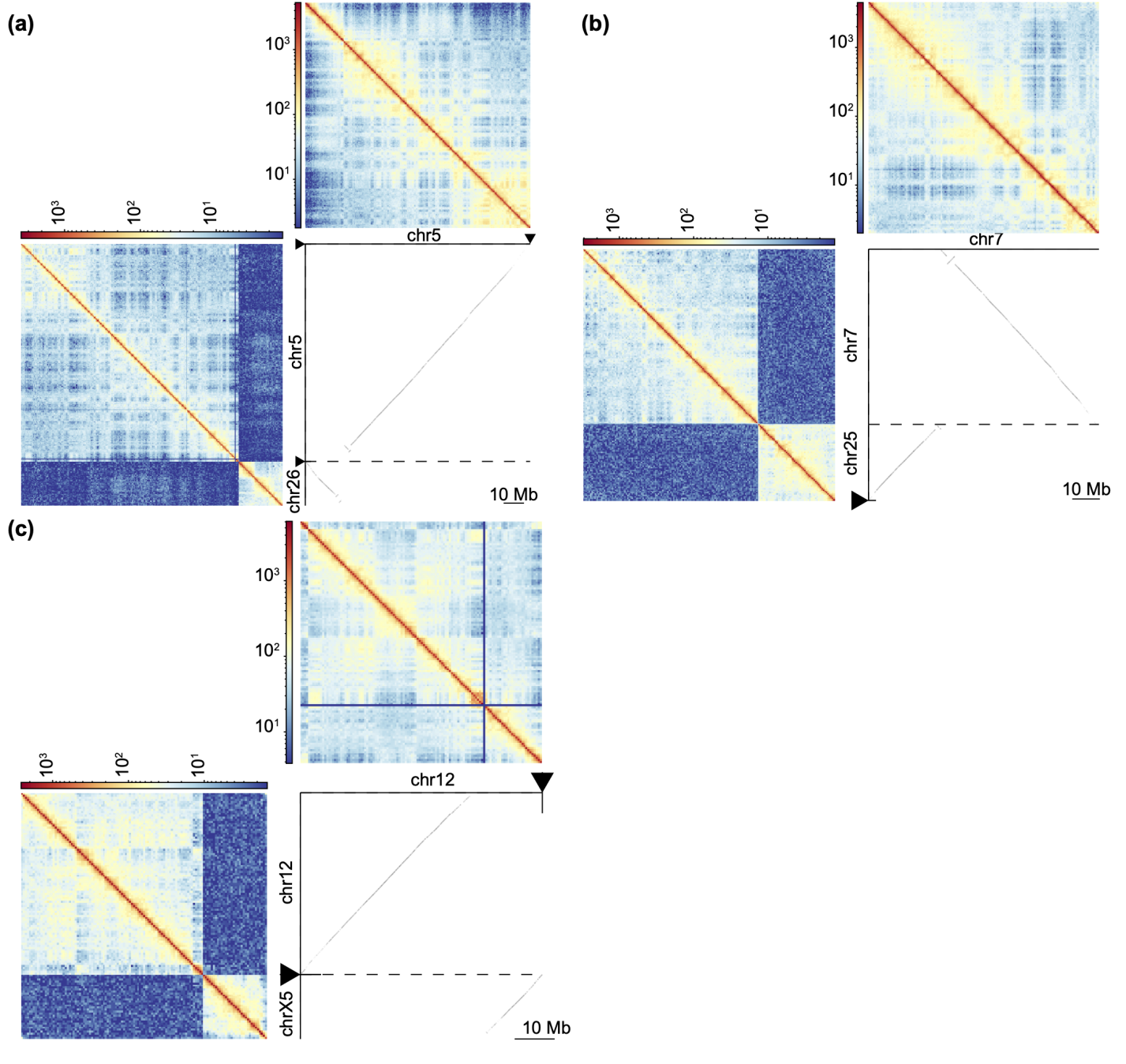


**Supplementary fig. S6. Confirmation of interchromosomal genomic rearrangement between platypus and echidna with Hi-C data under 500 Kb resolution.** Top heatmap: platypus, left heatmap: echidna. Coordinates of assembled telomeres are marked with black triangles.


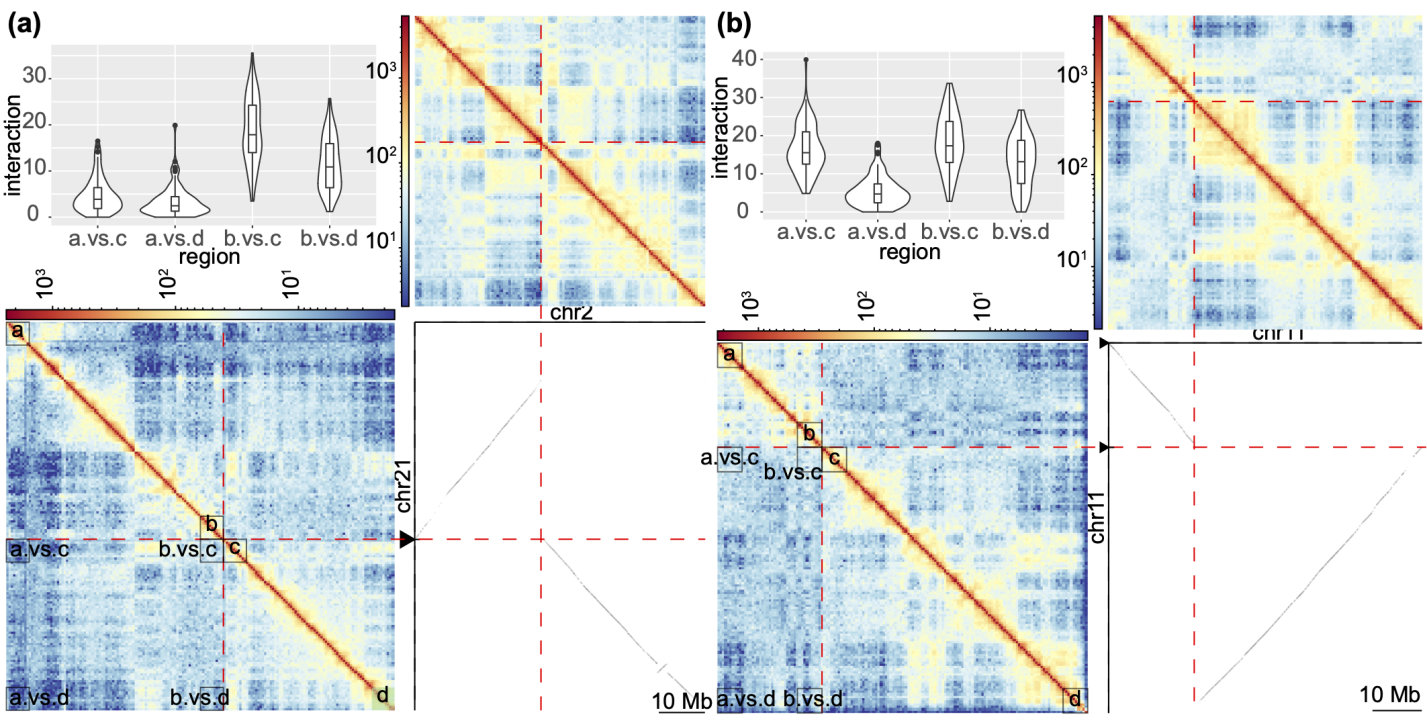


**Figure S7. Confirmation of intrachromosomal genomic rearrangement between platypus and echidna with Hi-C data under 500 Kb resolution.** Top heatmap: platypus, left heatmap: echidna. Coordinates of assembled telomeres are marked with black triangles. Intrachromosomal SV breakpoints are highlighted with red dashlines. Pairwise interaction strength in the four 5 Mb regions (a, b, c and d) were extracted to confirm the SVs.

**
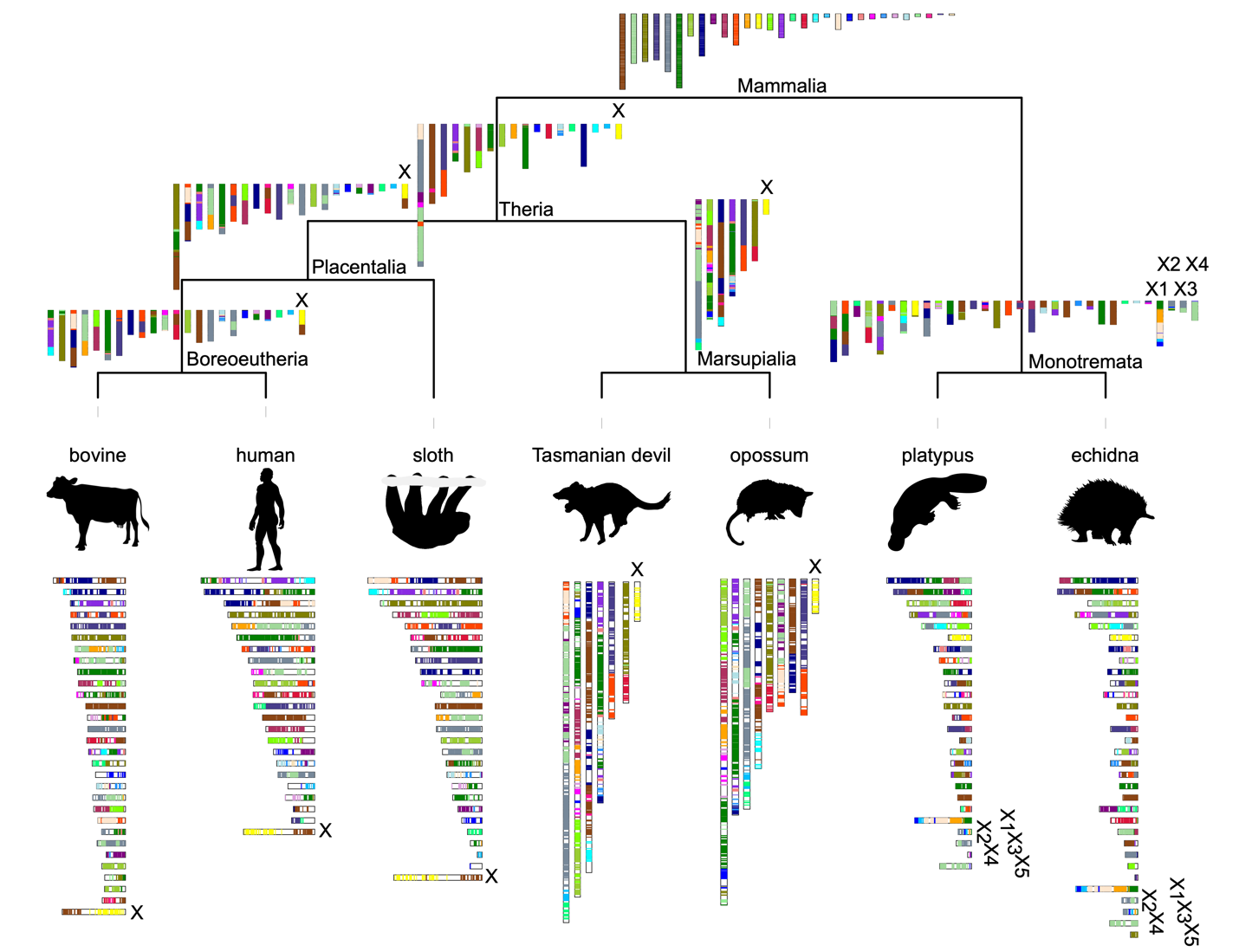
**

**Supplementary fig. S8. Ancestral karyotype reconstruction under 300 Kb resolution.**

**
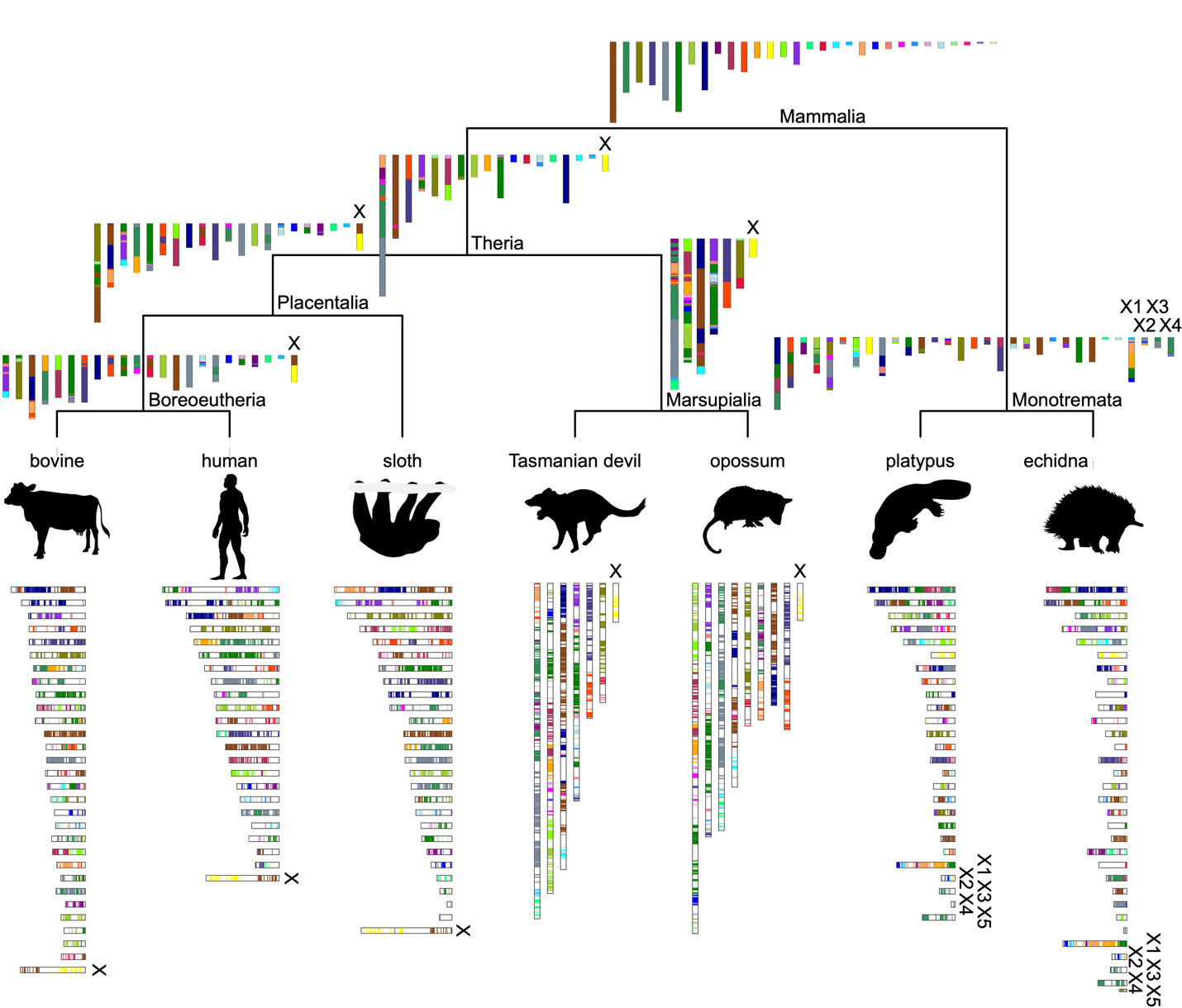
**

**Supplementary fig. S9. Ancestral karyotype reconstruction under 500 Kb resolution.**


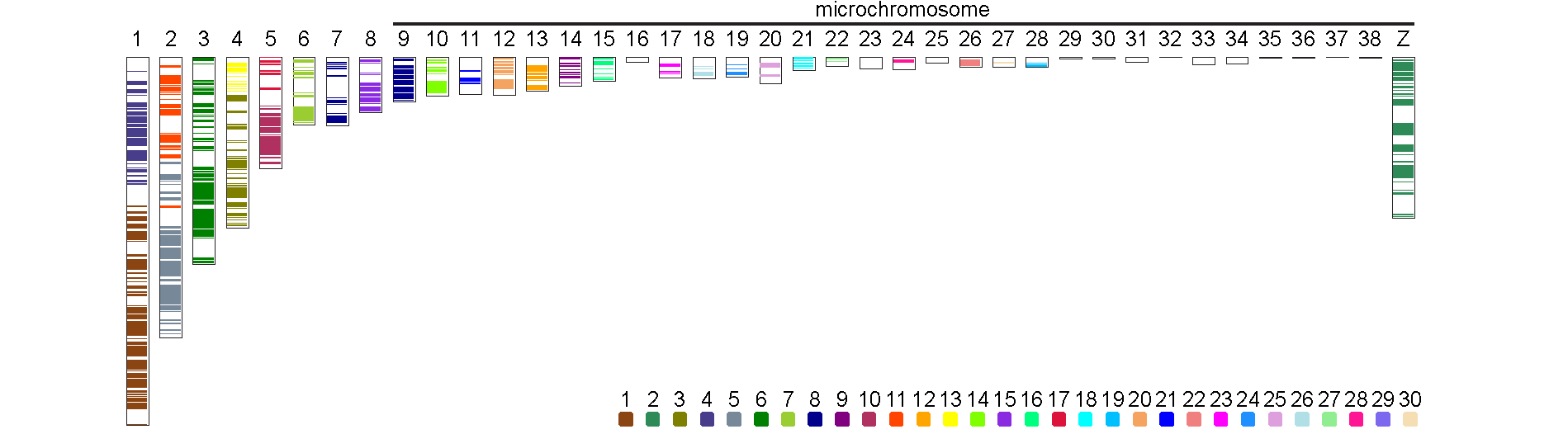


**Supplementary fig. S10. Mapping of mammalian ancestral chromosomes to chicken chromosomes.** Chicken chromosomes are color-coded based on the homology of the mammalian ancestral chromosomes. Some of the microchromosomes have no color as they are unable to map to mammalian ancestral chromosomes.


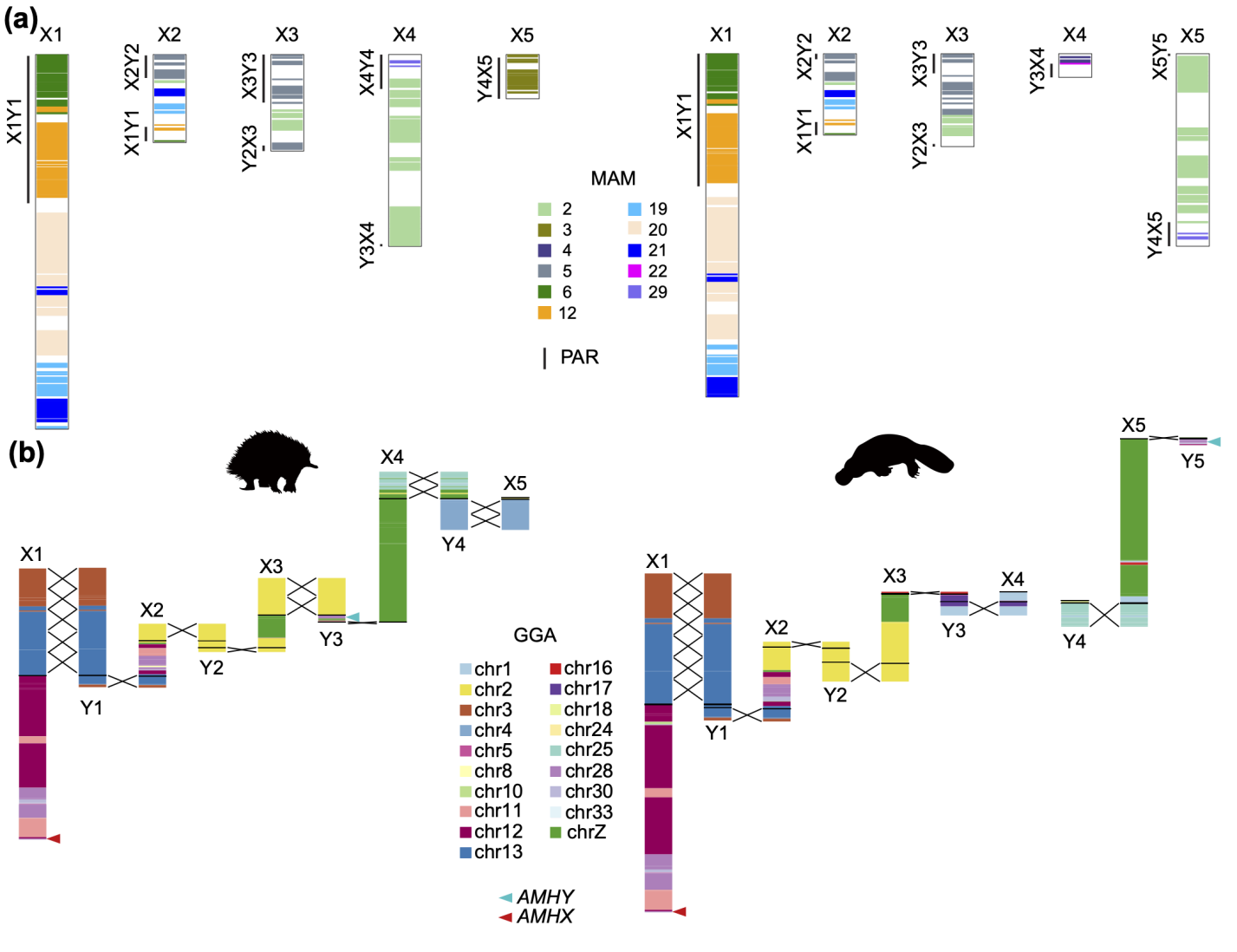


**Supplementary fig. S11. *In silico* chromosome painting of mammalian ancestral karyotype (a) and orthologous chicken sequences (b) to each echidna and platypus sex chromosome.** The recombination between PARs of X and Y are indicated by bar (a) and crosses (b). We also labeled the genomic coordinates of the putative sex-determining gene *AMHX/Y*. Note that since the ancestral reconstruction is built based on the genome with only autosomes and Xs, we were not able to map ancestral chromosomes to the monotreme Y chromosomes.


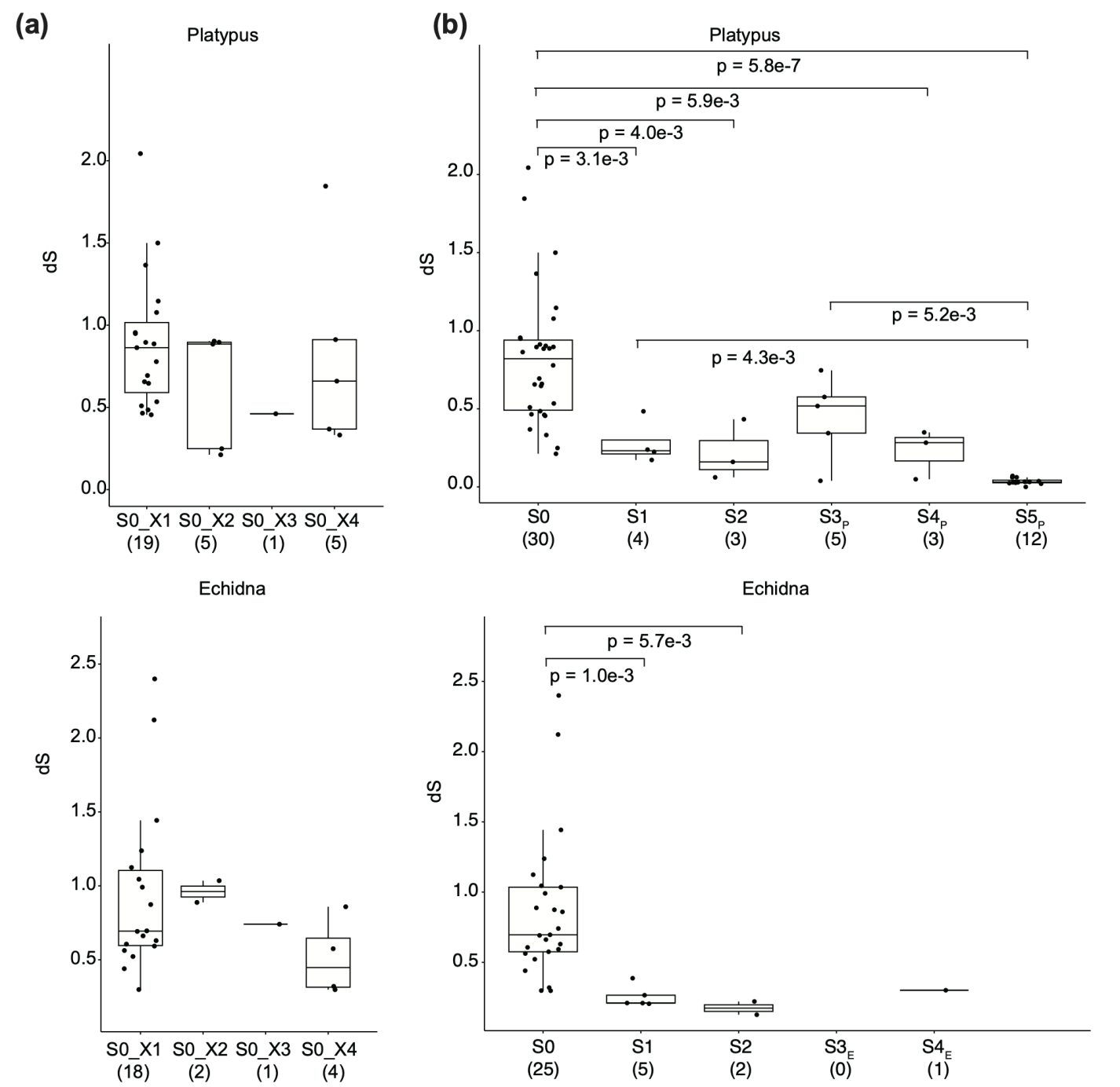


**Supplementary fig. S12. Gametolog pair dS distribution for each stratum.** (a) No siginficant difference can be found among the dS in S0_X1, S0_X2, S0_X3 and S0_X4 (note: platypus S0_X4 is on X5), suggesting that they may form ancetrally on one single chromome but then spread across the four ancestral Xs. (b) dS distribution for each stratum after merging S0_X1, S0_X2, S0_X3 and S0_X4 (or platypus S0_X5). Number in the bracket shows the number of gametolog pairs. The X chromosome locations are also noted for each stratum. Note that in echidna, only X or Y gametologue are found in S3_E_ (**supplementary table S15**) therefore no dS is available in the plot. We considered S1 and S2 as two different strata since they located on two different chromosomes and both are significantly differed from S0, therefore their recombination suppression was unlikely to at the same time. Conclusion was drawn for S3P, S4P and S5P for the similar reason.


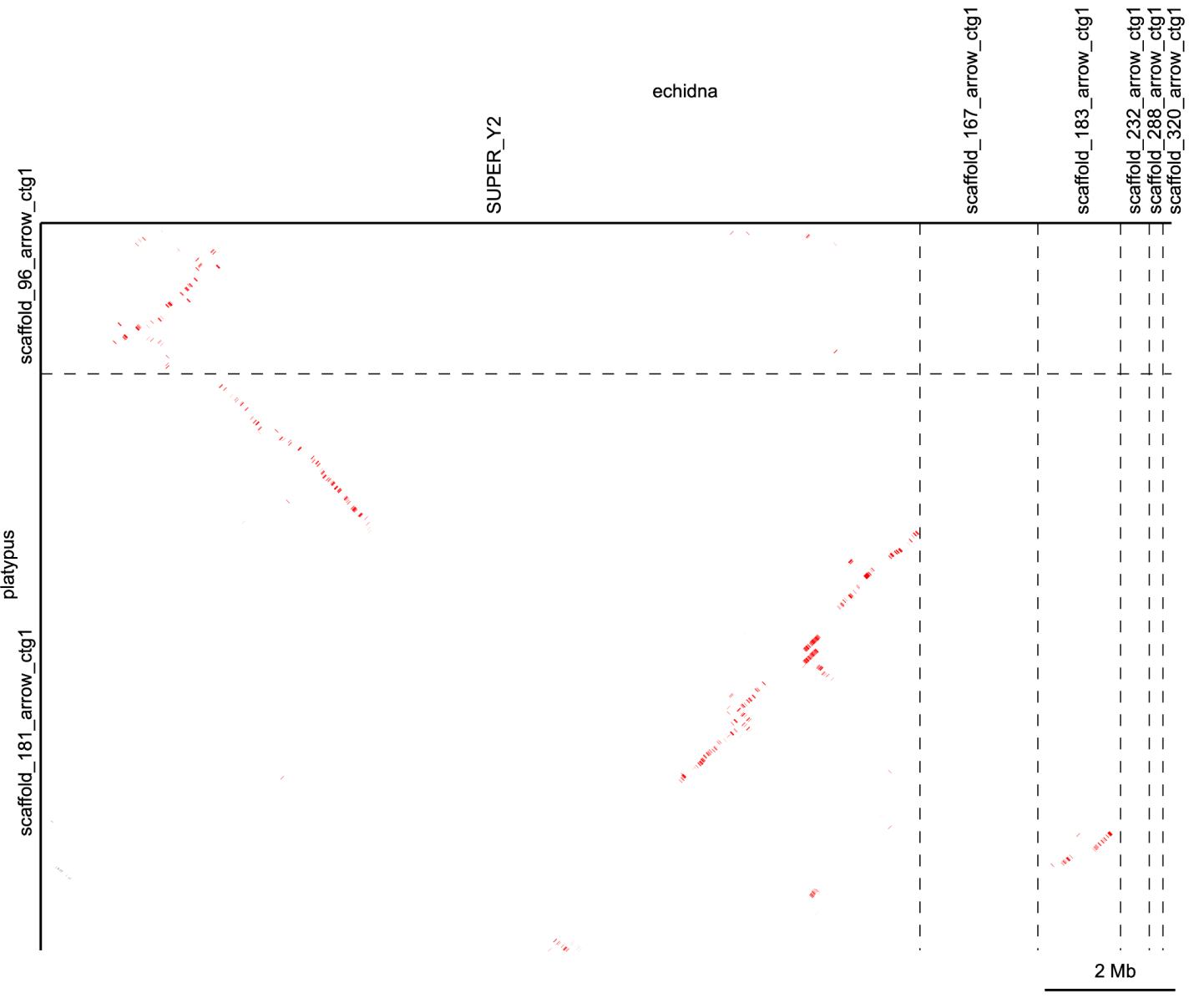


**Figure S13. Alignment between echidna Y3 and platypus Y5.**


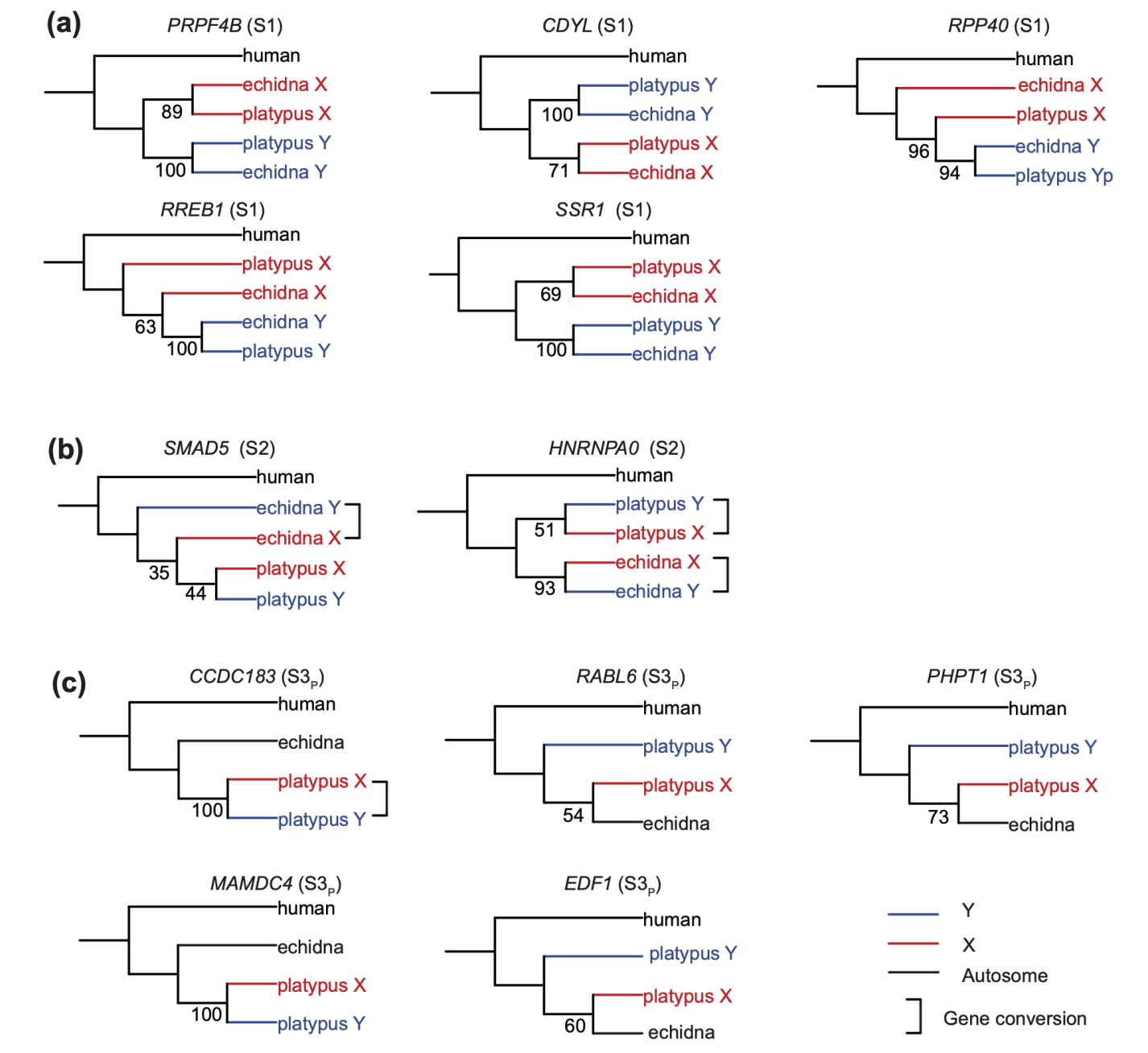


**Supplementary fig. S14. Phylogeny of S1 (a), S2 (b) and S3_P_ (c) X/Y gametolog and the orthologs.** (a) S4 gametolog pairs are clustered by sex chromosome instead of by species and no gene conversion is detected, suggesting that S4 originated before species divergence. (b) While S2 gametolog pairs are clustered by species instead by sex chromosomes, the bootstrap is low and gene conversion is detected between the X and Y of the same species*,* suggesting that S2 likely evolve ancestrally before species divergence. (c) S3_P_ gametologue pairs are clustered by species but the orthologs in echidna locates on autosome, therefore we consider the XY divergence happened independently in platypus. Red, X-linked gene, blue, Y-linked gene, black, autosomal gene. Bootstrap is noted at the internal nodes. Strong gene conversion signal (gene conversion ratio > 10%) is marked by link between genes. Pseudogenes are marked with a “p” suffix.


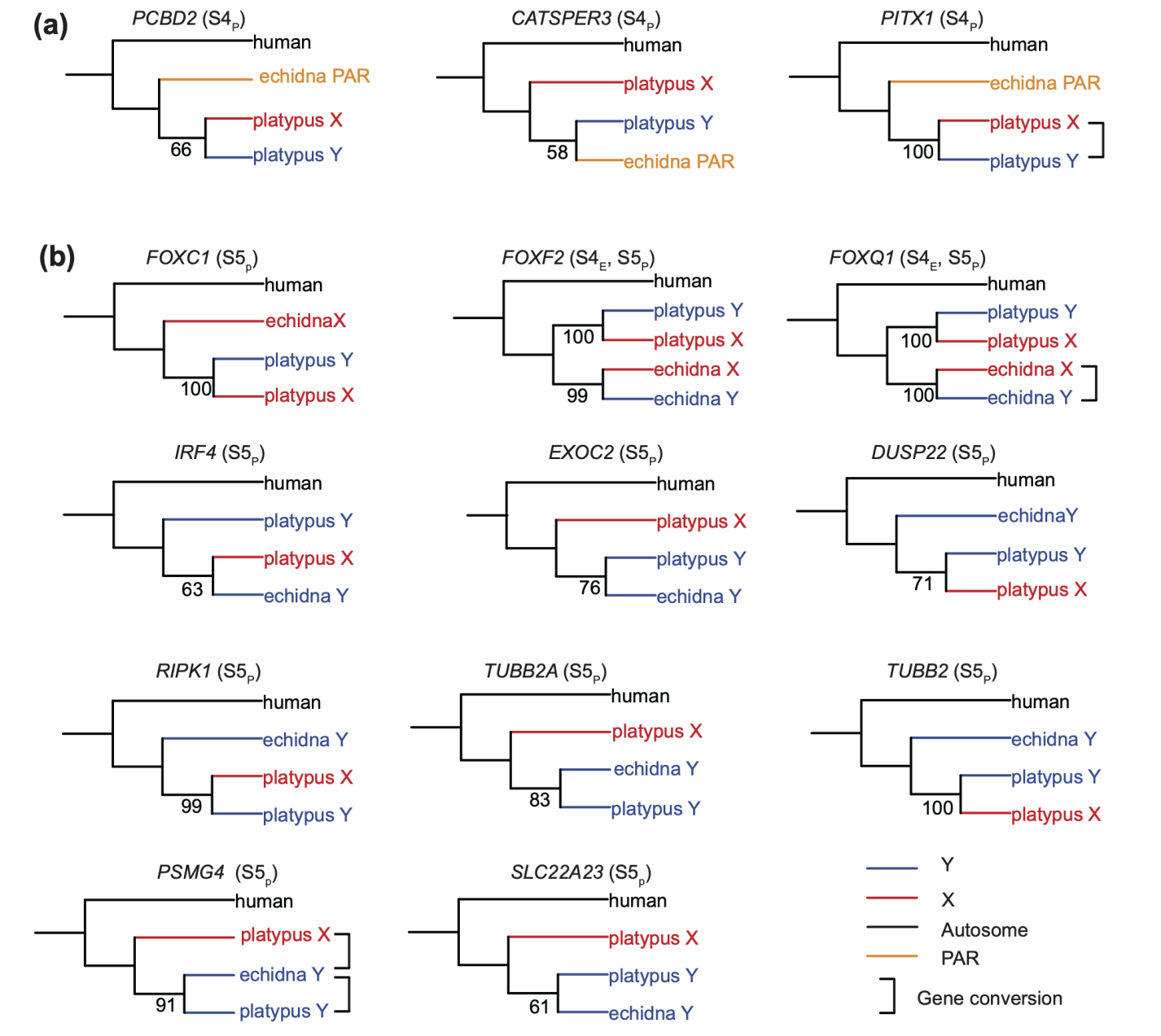


**Supplementary fig. S15. Phylogeny of S4_P_ (a), S5_P_ and S4_E_ (b) X/Y gametolog and the orthologs.** Gametolog pairs are clustered by species instead of by sex chromosomes and little gene conversion signal is detected, suggesting that these strata originated after species divergence. Red, X-linked gene, blue, Y-linked gene, black, autosomal gene, orange, PAR gene. Bootstrap is noted at the internal nodes. Strong gene conversion signal (gene conversion ratio > 10%) is marked by link between genes.


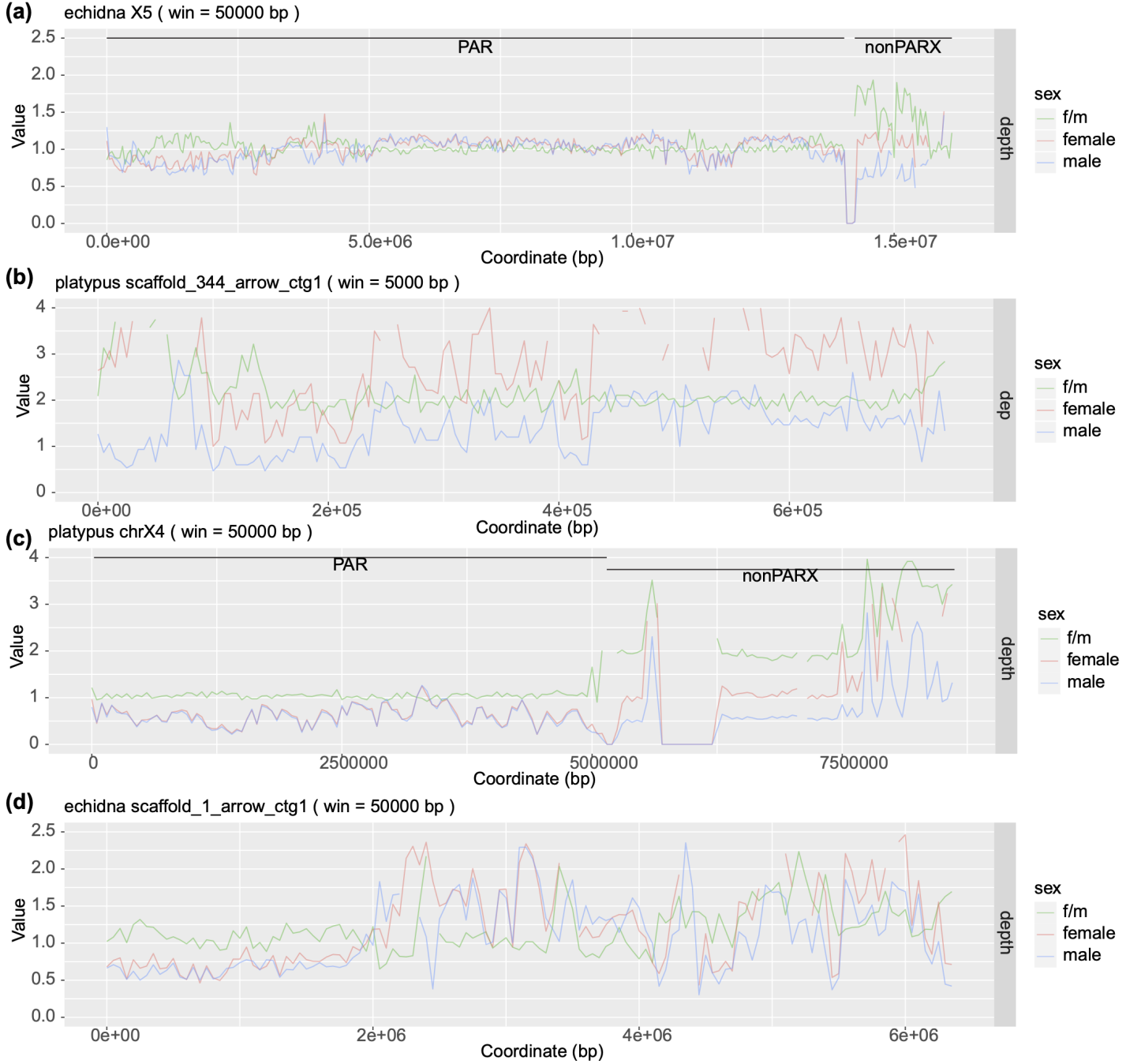


**Supplementary fig. S16. Normalized male and female sequencing depth in echidna chrX5, scaffold_344_arrow_ctg1 and platypus chrX4, scaffold_1_arrow_ctg1.** Red: female, blue: male, green: female-vs-male depth ratio (f/m). PAR and nonPARX on the assembled echidna X5 and platypus X4 are marked in the plot. The female-vs-male depth ratio is around two in echidna scaffold_344_arrow_ctg1, and the normalized depth of female and male is around one four and one, respectively, suggesting that the scaffold is a nonPARX but is collapsed during assembling.


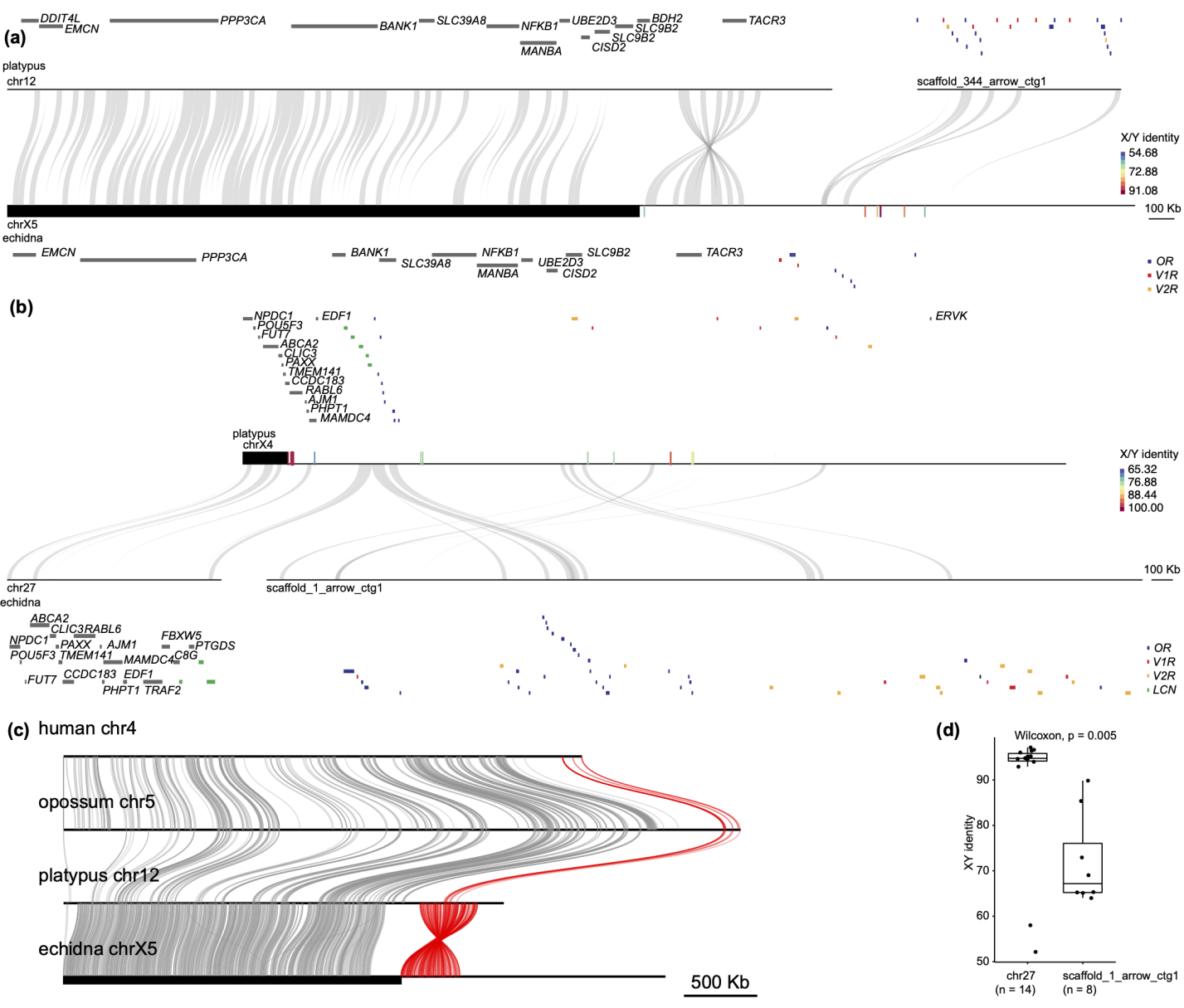


**Supplementary fig. S17. Alignment of the species specific X and the autosomal sequences in the other monotreme.** (a) Alignment between echidna chrX5:12,000,000-16,101,208 and the homologous sequences in platypus chr12:46,200,000-49,200,000 and scaffold_344_arrow_ctg1:1-741,479. Black bar indicates PAR. Duplicated genes encoding olfactory receptor (*OR*), vomeronasal receptor 1 (*V1R*) or 2 (*V2R*) are shown in blue, red and orange, respectively. X/Y identity is calculated in 1 Kb window and color-coded. (b) Alignment between platypus chrX4:4,800,000-8,639,456 and the homologous sequences in echidna chr27:1-1,000,000 and scaffold_1_arrow_ctg1:2,300,000-6,386,367. Black bar indicates PAR. Duplicated genes encoding olfactory receptor (*OR*), vomeronasal receptor 1 (*V1R*), 2 (*V2R*) or lipocalin (*LCN*) are shown in blue, red, orange and green, respectively. X/Y identity is calculated in 1 Kb window and color-coded. (c) Alignment of the inversion and its upstream region in human, opossum, platypus and echidna shows that the inversion happened in echidna. Alignment of the inversion region is highlighted in red. (d) Significant X/Y identity is found between the region closer to the PAR boundary (chr27) than the region distant to PAB (scaffold_1_arrow_ctg1). X/Y sequence identity is calculated in 1 Kb window. Two-sided Wilcoxon rank-sum test was performed.


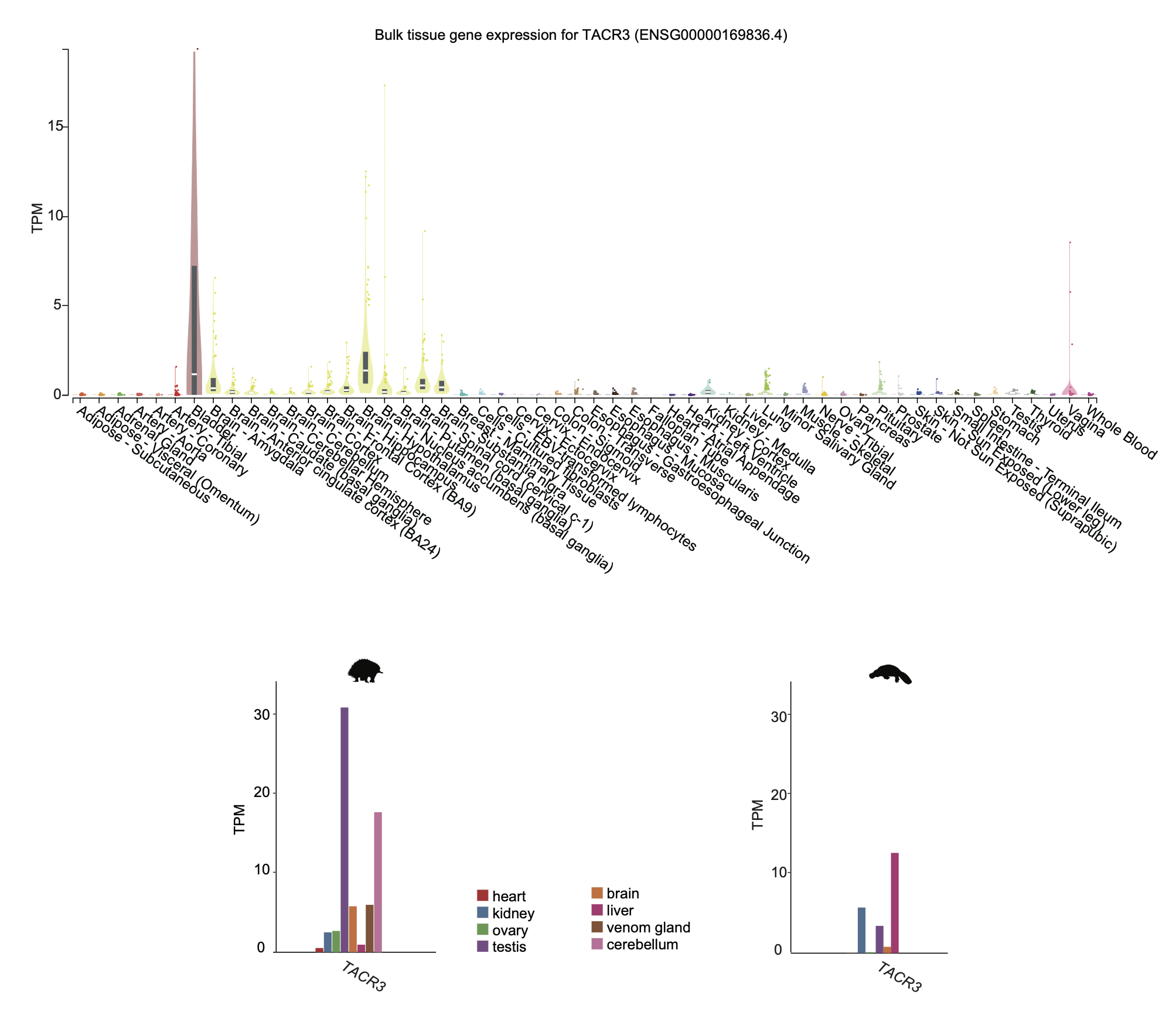


**Supplementary fig. S18. The expression profile of *TACR3* in human, echidna (X-linked) and platypus (autosomal).** Human expression data is obtained from: <https://www.gtexportal.org/home/gene/TACR3>.


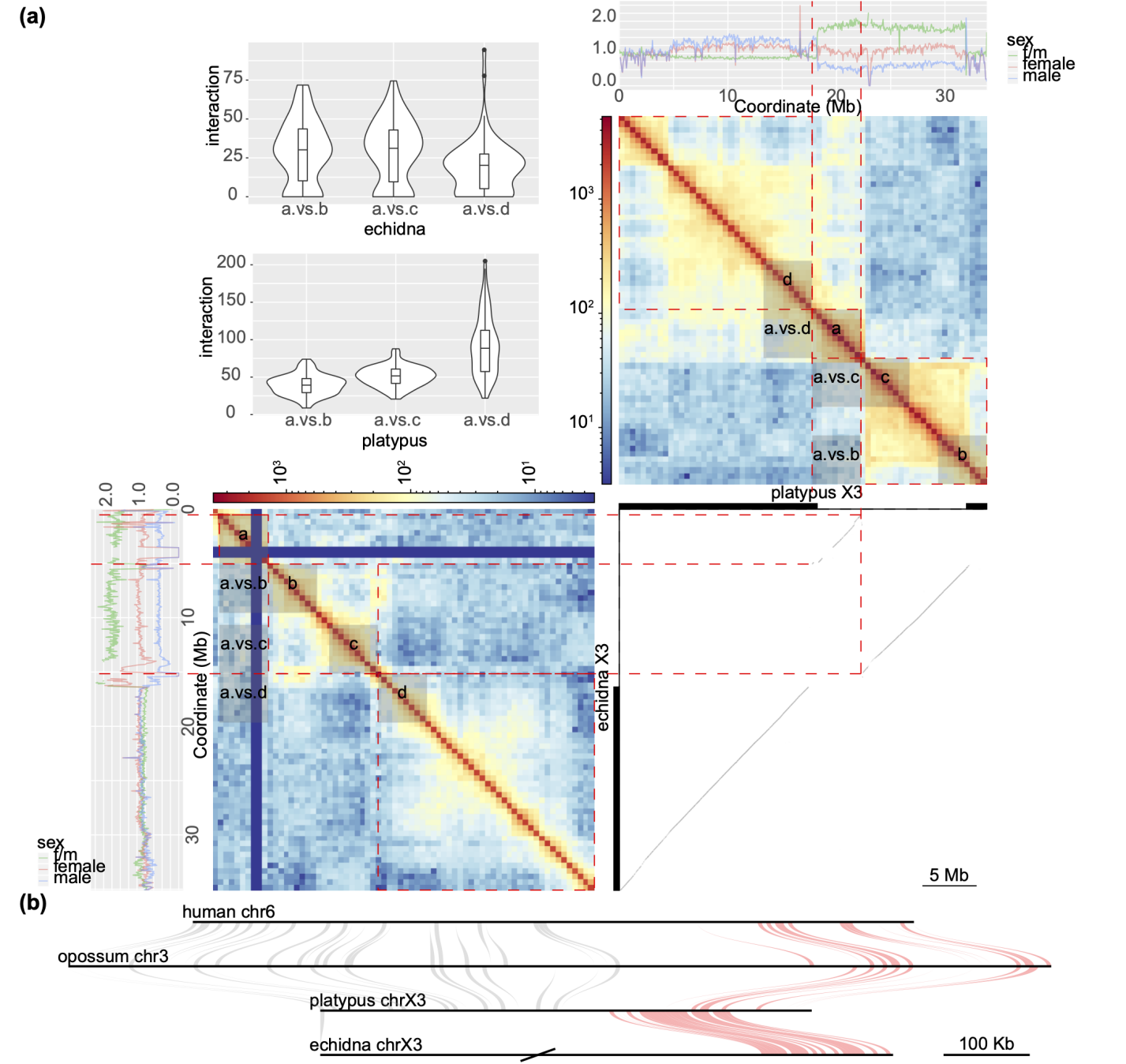


**Supplementary fig. S19. Confirmation of the translocation in X3.** (a) Confirmation of translocation between platypus X3 and echidna X3 with Hi-C data under 500 Kb resolution. Pairwise interaction strength in the four ~5 Mb regions (a, b, c and d) were extracted to confirm the translocation. Normalized male, female sequencing depth are also shown to indicate the involvement of a PAR-nonPARX transition. Normalized male and female depth, as well as female-vs-male depth ratio is also plotted along the two X3. PAR and gaps are marked as black and blue rectangles, respectively. (b) Alignment of the translocation and its upstream region in human, opossum, platypus and echidna. Alignment of the translocation region is highlighted in red. Visualized region include: human chr6:1,949,424-2,785,777, opossum chr3:345,825,794-346,965,677, platypus: chrX3:17,430,000-18,000,000 and echidna chrX3:4,913,378-16,395,920.
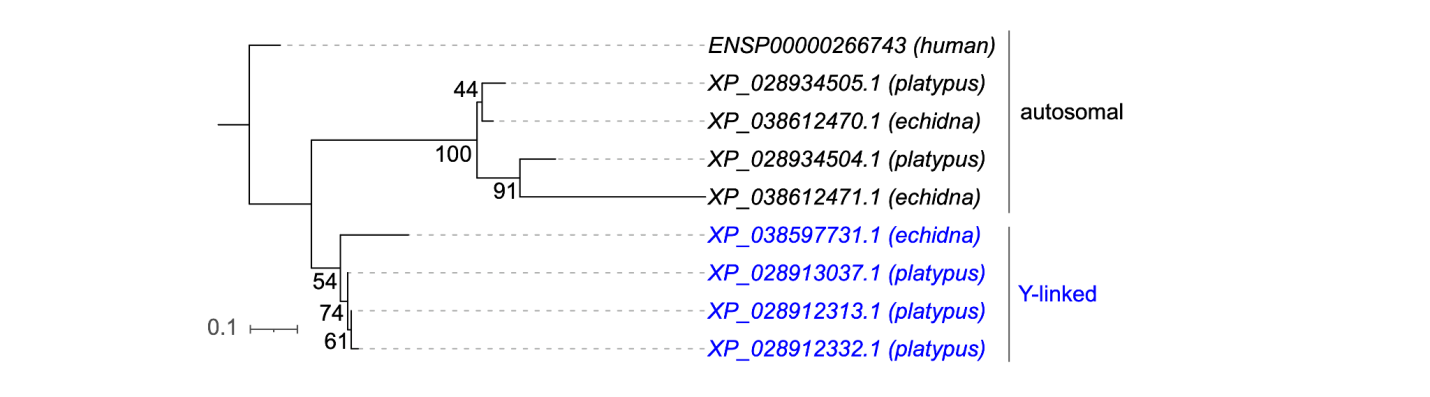


**Supplementary fig. S20. Gene phylogeny of *SYCP3*s and *SYCP3Y*s.**

**
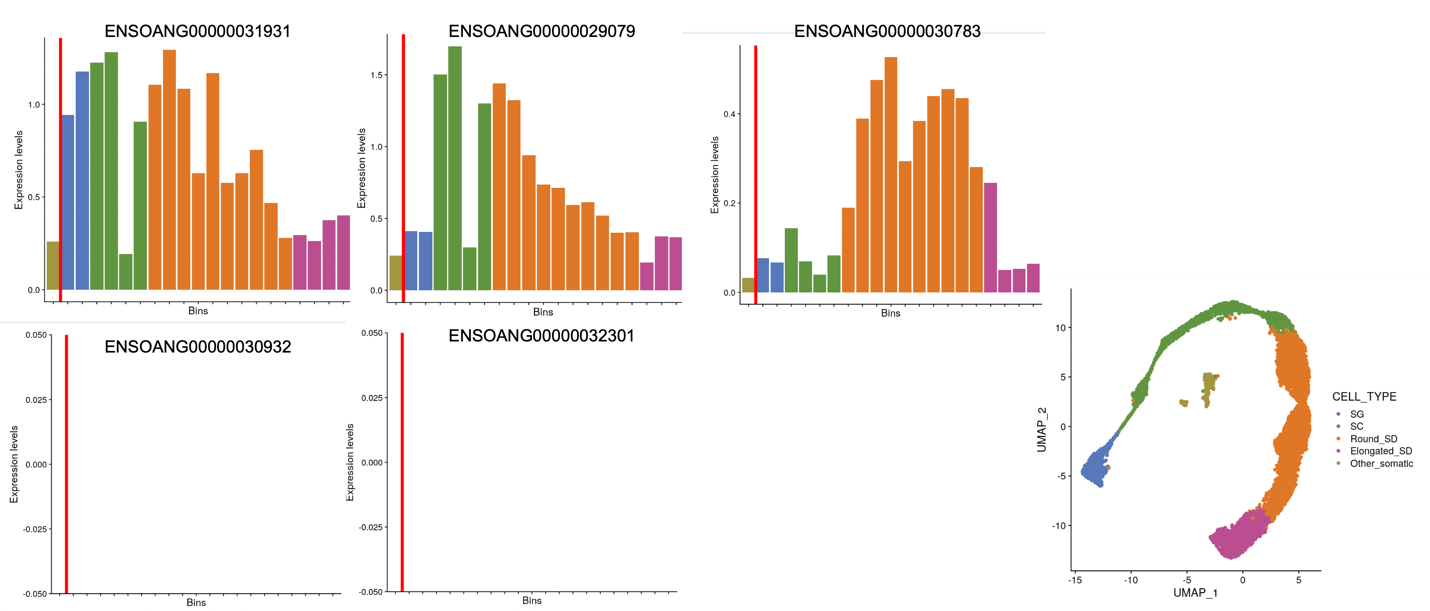
**

**Supplementary fig. S21. The expression profile of *SYCP3Y* in platypus snRNA-seq data.** Different colors indicate different cell types: blue, spermatogonia (SG), green, spermatocytes (SC), orange, round spe rmatids (round_SD), pink, elongated spermatids (elongated SD), yellow, other somatic cells. Plots were obtained from <https://apps.kaessmannlab.org/SpermEvol/>. Note that different annotation are used in Murat et al. and this study, therefore gene ID are different.
