# Supplementary material for: Chromosome-level echidna genome illuminates evolution of multiple sex chromosome system in monotremes
Source: Gigascience. 2025 Jan 9;14:giae112. doi: 10.1093/gigascience/giae112 (PMC11710854; doi:10.1093/gigascience/giae112)
Supplement: giae112_GIGA-D-24-00337_Revision_1 [file giae112_giga-d-24-00337_revision_1.pdf]

GigaScience

Chromosome-level echidna genome illuminates evolution of multiple-sex-chromosome system in monotremes

--Manuscript Draft--

**Manuscript Number:**	GIGA-D-24-00337R1
**Full Title:**	Chromosome-level echidna genome illuminates evolution of multiple-sex-chromosome system in monotremes
**Article Type:**	Research
**Funding Information:**	New Cornerstone Science Foundation — Prof. Guojie Zhang<br>Young Elite Scientists Sponsorship Program by CAST (2023QNRC001) — Dr Yang Zhou<br>Intramural Research Program of the National Human Genome Research Institute, National Institutes of Health — Dr Adam Phillippy, Dr Arang Rhie
**Abstract:**	Background: A thorough analysis of genome evolution is fundamental for biodiversity understanding. The iconic monotremes (platypus and echidna) feature extraordinary biology. However, they also exhibit rearrangements in several chromosomes, especially in the sex chromosome chain. Therefore, the lack of a chromosome-level echidna genome has limited insights into genome evolution in monotremes, in particular the multiple sex chromosomes complex. Results: Here, we present a new long-reads-based chromosome-level short-beaked echidna (Tachyglossus aculeatus) genome, which allowed the inference chromosomal rearrangements in the monotreme ancestor (2n = 64) and each extant species. Analysis of the more complete sex chromosomes uncovered homology between one Y chromosome and multiple X chromosomes, suggesting that it is the ancestral X that has undergone reciprocal translocation with ancestral autosomes to form the complex. We also identified dozens of ampliconic genes on the sex chromosomes, with several ancestral ones expressed during male meiosis, suggesting selective constraints in pairing the multiple sex chromosomes. Conclusion: The new echidna genome provides an important basis for further study of the unique biology and conservation of this species.
**Corresponding Author:**	Guojie Zhang<br>Zhejiang University<br>Hangzhou, Zhejiang CHINA
**Corresponding Author Secondary Information:**	
**Corresponding Author's Institution:**	Zhejiang University
**Corresponding Author's Secondary Institution:**	
**First Author:**	Guojie Zhang
**First Author Secondary Information:**	
**Order of Authors:**	Guojie Zhang
	Yang Zhou
	Jiazheng Jin
	Xuemei Li
	Gregory Gedman
	Sarah Pelan
	Chuan Jiang
	Olivier Fedrigo

Powered by Editorial Manager® and ProduXion Manager® from Aries Systems Corporation

	Kerstin Howe
	Adam Phillippy
	Arang Rhie
	Erich D. Jarvis
	Frank Grutzner
	Qi Zhou
**Order of Authors Secondary Information:**	
**Response to Reviewers:**	Please see the responese in the attached "Response_letter.pdf"
**Additional Information:**	
**Question**	**Response**
Are you submitting this manuscript to a special series or article collection?	No
**Experimental design and statistics**<br><br>Full details of the experimental design and statistical methods used should be given in the Methods section, as detailed in our Minimum Standards Reporting Checklist. Information essential to interpreting the data presented should be made available in the figure legends.<br><br>Have you included all the information requested in your manuscript?	Yes
**Resources**<br><br>A description of all resources used, including antibodies, cell lines, animals and software tools, with enough information to allow them to be uniquely identified, should be included in the Methods section. Authors are strongly encouraged to cite Research Resource Identifiers (RRIDs) for antibodies, model organisms and tools, where possible.<br><br>Have you included the information requested as detailed in our Minimum Standards Reporting Checklist?	Yes
**Availability of data and materials**	Yes

Powered by Editorial Manager® and ProduXion Manager® from Aries Systems Corporation

All datasets and code on which the conclusions of the paper rely must be either included in your submission or deposited in publicly available repositories (where available and ethically appropriate), referencing such data using a unique identifier in the references and in the "Availability of Data and Materials" section of your manuscript.<br><br>Have you have met the above requirement as detailed in our Minimum Standards Reporting Checklist?	

Powered by Editorial Manager® and ProduXion Manager® from Aries Systems Corporation

Manuscript

Click here to access/download;Manuscript;Manuscript_Dec 3.docx

Chromosome-level echidna genome illuminates evolution of multiple-sex-chromosome system in monotremes

Author list

Yang Zhou[1,2,†], Jiazheng Jin[2, †], Xuemei Li[3], Gregory Gedman[4], Sarah Pelan[5], Arang Rhie[6], Chuan Jiang[7], Olivier Fedrigo[8], Kerstin Howe[5], Adam M. Phillippy[6], Erich D. Jarvis[4,9], Frank Grutzner[10], Qi Zhou[*,11,12,13], Guojie Zhang[*,13,14,15]

1. State Key Laboratory of Agricultural Genomics, BGI Research, Shenzhen 518083, China
2. BGI Research, Wuhan 430074, China
3. College of Life Sciences, University of Chinese Academy of Sciences, Beijing 100049, China
4. Laboratory of Neurogenetics of Language, The Rockefeller University, New York, New York, USA 10065
5. Wellcome Sanger Institute, Cambridge, UK
6. Genome Informatics Section, Computational and Statistical Genomics Branch, National Human Genome Research Institute, National Institutes of Health, Bethesda, MD USA
7. College of Wildlife and Protected Area, Northeast Forestry University, Harbin 150040, China

8. The Vertebrate Genome Lab, The Rockefeller University, New York, New York, USA 10065
9. Howard Hughes Medical Institute, Chevy Chase, Maryland, USA 20815
10. School of Biological Sciences, The Environment Institute, The University of Adelaide, 5005 Adelaide, Australia
11. The MOE Key Laboratory of Biosystems Homeostasis & Protection and Zhejiang Provincial Key Laboratory for Cancer Molecular Cell Biology, Life Sciences Institute, Zhejiang University, Hangzhou 310058, Zhejiang
12. Center for Reproductive Medicine, The 2nd Affiliated Hospital, School of Medicine, Hangzhou 310052, Zhejiang
13. Centre for Evolutionary & Organismal Biology, Zhejiang University School of Medicine, Hangzhou 310058, China
14. Liangzhu Laboratory, Zhejiang University Medical Center, Hangzhou, China
15. Women's Hospital, School of Medicine, Zhejiang University, Hangzhou, China

* Correspondence address: Guojie Zhang, Centre for Evolutionary & Organismal Biology, Zhejiang University School of Medicine, Hangzhou 310058, China, Emails: guojiezhang@zju.edu.cn; Qi Zhou, The MOE Key Laboratory of Biosystems Homeostasis & Protection and Zhejiang Provincial Key Laboratory for Cancer Molecular Cell Biology, Life Sciences Institute, Zhejiang University, Hangzhou 310058, Zhejiang, Email: zhouqi1982@zju.edu.cn

† Yang Zhou and Jiazheng Jin contributed equally to this work

ORCID iDs: Yang Zhou [0000-0003-1247-5049]; Jiazheng Jin; Xuemei Li; Gregory Gedman [0000-0001-6819-2019]; Sarah Pelan [0000-0001-8729-685X]; Chuan Jiang [0009-0002-5563-162X]; Olivier Fedrigo [0000-0002-6450-7551]; Kerstin Howe [0000-0003-2237-513X]; Adam Phillippy [0000-0003-2983-8934]; Arang Rhie [0000-0002-9809-8127]; Erich D Jarvis [0000-0001-8931-5049]; Frank

Grutzner [0000-0002-3088-7314]; Qi Zhou [0000-0002-7419-2047]; Guojie Zhang [0000-0001-6860-1521].

Abstract

Background: A thorough analysis of genome evolution is fundamental for biodiversity understanding. The iconic monotremes (platypus and echidna) feature extraordinary biology. However, they also exhibit rearrangements in several chromosomes, especially in the sex chromosome chain. Therefore, the lack of a chromosome-level echidna genome has limited insights into genome evolution in monotremes, in particular the multiple sex chromosomes complex. **Results**: Here, we present a new long-reads-based chromosome-level short-beaked echidna (*Tachyglossus aculeatus*) genome, which allowed the inference chromosomal rearrangements in the monotreme ancestor (2n = 64) and each extant species. Analysis of the more complete sex chromosomes uncovered homology between one Y chromosome and multiple X chromosomes, suggesting that it is the ancestral X that has undergone reciprocal translocation with ancestral autosomes to form the complex. We also identified dozens of ampliconic genes on the sex chromosomes, with several ancestral ones expressed during male meiosis, suggesting selective constraints in pairing the multiple sex chromosomes. **Conclusion**: The new echidna genome provides an important basis for further study of the unique biology and conservation of this species.

Keywords: echidna, monotreme, sex chromosome evolution, multiple sex chromosomes.

Introduction

An understanding of chromosome evolution has been fundamental for mammalian comparative studies [1, 2]. Large-scale chromosomal rearrangement is an important source of genetic variation and has contributed to adaptation and speciation, and dissection of the underlying mechanisms requires high quality genomes [3-5]. High quality genomes are also an important basis for understanding species biology and for long-term application in species conservation [6-

9]. Monotremes, including platypus (*Ornithorhynchus anatinus*) and four echidna species (Tachyglossidae), comprise the sister group of therians and the most basal mammalian lineage. Due to their unique phylogenetic position in mammal evolution, these species hold the key to understanding the evolutionary changes of major mammalian lineages since their divergence from the common ancestor with other mammals [9-11]. In addition, monotremes are iconic in Australia and much of their extraordinary biology is still unexplored. These species have a karyotype with seven or eight pairs of large chromosomes and many small chromosomes reminiscent of the microchromosomes in reptiles but of different origins [12, 13]. Compared to therians, the monotreme karyotypes are highly rearranged [14]. Thus, the monotreme genomes are valuable for gaining the insight of mammalian and monotreme genome evolution, as well as understanding the changes in genome architecture of reptiles and mammals.

One of the most remarkable features of the genome of egg-laying mammals are their special sex chromosome system, consisting of multiple X and Y chromosomes. In most sex chromosome systems, the sex chromosomes typically exist as one pair, with recombination suppression often initially driven by intrachromosomal rearrangement such as inversion [15, 16]. In some lineages, one of the sex chromosomes would fuse with an autosome, leading to a trivalent sex chromosome system. For example, in the male Japan Sea stickleback, the ancestral Y is fused with an ancestral autosome LG9, resulting in a X1X2Y system where the X1 is the ancestral X chromosome (LG19) and X2 is the neo-X chromosome resulted from the fusion event (LG9) [17]. Such fusions may offer evolutionary advantages, such as enabling sex-biased inheritance of genes favored by one sex [18] and driving speciation events [17]. However, it may also introduce difficulty in pairing and segregating the multiple sex chromosomes into offspring cells [19] and disruption the gene expression during spermatogenesis due to meiotic sex chromosome inactivation [20]. The evolutionary process of the multiple sex chromosomes in monotreme and its consequences can be even more complicated. In male there are nine

(echidna) or ten sex chromosomes (platypus), pairing in a head-to-tail manner via the pseudoautosomal regions (PARs) and forming a meiotic chromosome chain [21-23]. It is established now that this system originated independently from the therian XY sex chromosome system [10, 23] and probably evolved via series of reciprocal translocation events between the proto sex chromosomes and autosomes [9, 10, 24]. Therefore, the upstream sex determinant genes are distinct between the two mammalian groups, with *SRY* being the key player in therian mammal and *AMHY* being the most likely candidate in monotreme [10]. This complex system has furthermore undergone independent evolution after the two species diverged from each other. There are five Xs and five Ys in male platypus while there are five Xs but only four Ys in male echidna [23]. Of these chromosomes, the third Y and the fourth X chromosomes (Y3, X4) of platypus and the fifth X chromosome (X5) of echidna are homologous to the autosome in the other species [23], and are considered to evolve via reciprocal translocation after their speciation [25]. Therefore, the complicated system in monotreme serves as a model example to demonstrate the unusual driving force of high frequency reciprocal translocations during sex chromosome evolution, and the resulting constraint of the multiple sex chromosome system such as the need to successfully segregation of multiple X and Y into different sperms.

Previously we have tracked the evolution of monotreme genome and particularly sex chromosome with a chromosome-level platypus genome and a draft echidna genome [9]. However, the draft echidna genome, especially the Y chromosomes, are still incomplete and largely fragmented in sequence. These two major lineages in monotreme have diverged around 55 million years ago [9] with an average dS value in coding regions at around 0.1907, implying a substantial divergence on their genetic properties. A more complete echidna genome is to provide a more comprehensive understanding on the evolution across major mammal groups and the divergence within monotreme lineage. In this study, we produced an improved chromosome-level short-beaked echidna *T. aculeatus* (NCBI:txid9261) assembly, to further

explore the genomic features of these young and unusual sex chromosomes. We also conducted the first genome-wide screen of the ampliconic genes on the monotreme sex chromosomes, unveiling potential selection constraints on the multiple sex chromosome systems.

Results

A chromosome-level short-beaked echidna genome

We utilized PacBio long reads, 10X-linked reads, Bionano and Hi-C data to produce the chromosome-level genome assembly for a male short-beaked echidna, following the VGP assembly pipeline v1.6 (**supplementary tables S1 & S2**). Briefly, PacBio long reads were first used to construct contigs, and scaffolds were generated iteratively with three scaffolding technologies (i.e., 10X, Bionano and Hi-C). We further identified the sex-linked sequence based on the sequencing depth difference between male and female. The new PacBio-based assembly includes 27 autosomes, five X and four Y chromosomes, with a ~966-fold improvement on contig N50 compared to the published short read-based assembly (GCA_015598185.1) (**supplementary table S2**). Telomeres have been assembled on 28 of the total 32 chromosomes (**Fig. 1A, supplementary table S3**). Notably, 183.44 Mb and 9.18 Mb of the X and Y sex differentiated regions on the five X (X-Div, X divergent) and four Y chromosomes (Y-Div, Y divergent) respectively were identified (**supplementary table S4, supplementary fig. S1**). We also utilized the Hi-C data to filter and infer the possible chromosome origin for previously unplaced X, Y and PAR scaffolds (**supplementary table S5, supplementary fig. S2**). In summary, 99.82% and 98.25% of the assembled X-Div and Y-Div sequences can be assigned to the nine sex chromosomes, representing a more continuous and complete sequence compared to the previous assembly (**supplementary table S2**). Based on the

estimation from karyotype images in Rens et al. [23], we found that most of the nine chromosomes have over 98% completeness except Y3 (21.34%) and X5 (22.44%) which have accumulated exceptionally high repeat contents [23] (**supplementary table S6**). Evaluation by male specific transcripts [10] also showed that all male-specific genes are fully covered except only one is fragmented (coverage < 50%) in the new PacBio-based assembly (**supplementary table S7**). In contrast, two were fragmented and three were missing in the previous assembly (**supplementary table S7**).

Alignment between the new and old echidna assemblies (PacBio-based GCA_015852505.1 v.s. Illumina-based GCA_015598185.1) revealed that 66 large putative structural variants (>100 Kb). Although the sequenced individuals are collected from different locations, these large putative structural variants were likely to be mis-assembly artifacts in either assembly. Based on the examination of raw PacBio, 10X-linked reads and Hi-C data, we found that the genome structure of 65 regions were correct in the new echidna genome (**Fig. 1A, supplementary table S8, supplementary fig. S3**); only one was an error in our new assembly which has been manually fixed in the latest release. Moreover, ~74.27% gaps or an estimated size of 179.51Mb sequences in the previous assembly were closed in the new PacBio-based assembly (**supplementary fig. S4, supplementary table S9**), contributing to the new annotation of 21,334 exons from 6,493 protein-coding genes. This is consistent with the improved Benchmarking Universal Single-Copy Orthologs evaluation which shows that 90.80% of the 9,226 mammalian conserved orthologs are complete and presented as single-copy in the PacBio-based assembly, compared to only 59.20% in the Illumina-based one (**supplementary fig. S4, supplementary table S2**).

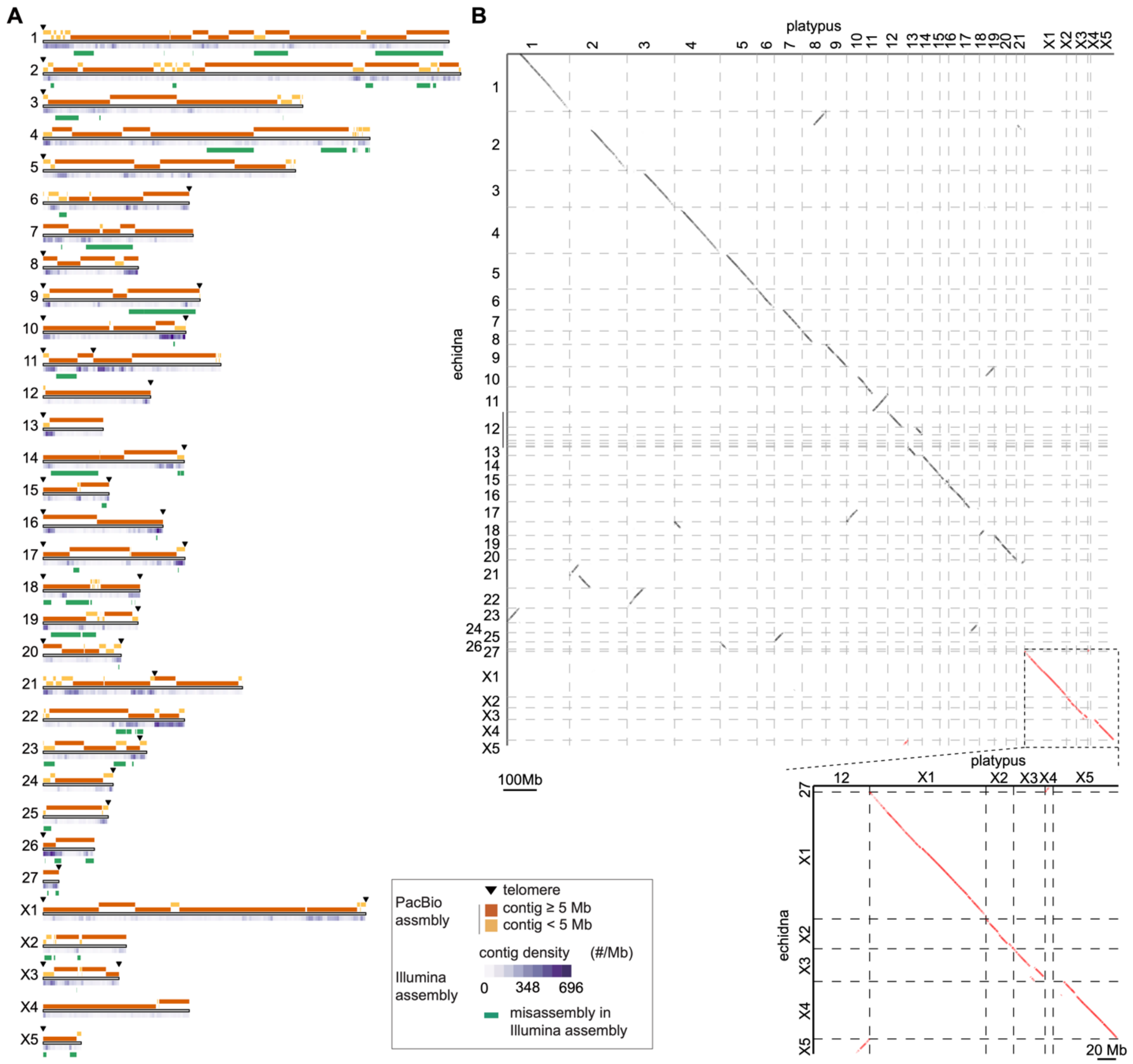

Fig. 1. Genome assembly of short-beak echidna.

(A) Schematic plot mapping of the assembled contigs onto echidna chromosomes. Orange rectangles on top represent contigs ≥5 Mb in the new assembly, and yellow rectangles represent contigs <5 Mb. The heatmap below represents the density of contigs in Illumina-based assembly mapping onto chromosomes counting based on the number of contigs per 1 Mb region. Assembled telomere sequences are shown in black triangles in the plot. Coordinates of Illumina-based assembly artifacts corrected in the PacBio-based assembly are shown in green.

(B) Dotplot showing the genome synteny between platypus and echidna. The overall synteny (86.94%) is well kept between the two species, but there are still 2.60% and 10.46% intra- and inter-chromosomal rearrangements, respectively. Zoom-in alignment shows that most sex chromosomes are in high synteny and homology, except platypus X4 and echidna X5 which are homologous to the autosome in the other species.

Genome evolution of platypus and echidna

There are 2n=63 and 64 chromosomes in male and female short-beaked echidna and long-beaked echidna, respectively, while there are only 2n=52 chromosomes in platypus [23, 26] suggesting that chromosome fusion or fission events might have occurred since platypus-echidna divergence. Direct comparison between the two species uncovered other genomic rearrangement including inversions and translocations (**Fig. 1B, supplementary figs. S5 to S7**). To systematically investigate evolution of the genomic rearrangements during the divergence of monotremes, especially those involved in sex chromosome evolution, we reconstructed the karyotype of monotreme ancestor with chromosomal assemblies of placentals (human, bovine and sloth), marsupials (opossum and Tasmanian devil), monotremes (platypus and echidna) and reptilian outgroups (chicken, turtle and common wall lizard), under 300 Kb and 500 Kb resolution. Based on the genomic data and the previous FISH and *in silico* reconstruction [14, 27-29], we inferred an ancestral karyotype of 2n=64 of the monotreme most recent common ancestor (MRCA), including 28 pairs of autosomes and 4 pairs of sex chromosomes. Although this number is closer to the karyotype number of echidna than that of platypus, the echidna genome experienced more lineage-specific rearrangement than platypus (**Fig. 2A, supplementary figs. S8 & S9, supplementary tables S10 to S12**). Thirteen monotreme ancestral chromosomes (MON8, 11, 14, 18, 19, 20, 24, 25, 28 and X1-4) were preserved as individual chromosomes in both species, while others have experienced genomic rearrangement

events in either or both species, while others have experienced genomic rearrangement events in either or both species (**Fig. 2B**). For example, the breakage of MON4 produced two echidna chromosomes while it has remained intact as chr3 in platypus; the fusion of MON12 and MON22 produce one echidna chromosome while remained separate as chr10 and chr17 in platypus (**Fig. 2B**). These interchromosomal rearrangements were consistent with the previous findings by FISH experiment [23]. However, the whole genome alignment also provided refined details in intrachromosomal rearrangements. For example, the echidna chr11 and chr21 experienced intrachromosomal inversion after divergence from platypus, indicated by both the ancestral reconstruction (**Fig. 2B**) as well as the telomere remnant at the inversion breakpoints (**supplementary fig. S7**). Interestingly, the centromere monomer sequences of the two species are distinct [9], probably associated with the chromosomal rearrangements. Furthermore, recent studies of vertebrate chromosome evolution suggested that the avian microchromosomes can be dated back to the ancestor of the amniote [30], and the mammalian macrochromosomes likely evolved by a series of chromosome fusions and translocations [31]. Our reconstruction confirmed this inference by finding that each single chicken microchromosome can be mapped to one mammalian ancestral chromosome (**supplementary fig. S10**).

The ancestral karyotype reconstruction also provides novel insight into the dynamic evolution of the monotreme sex chromosome complex. Four of the five extant sex chromosomes (platypus chrX1-X3, chrX5 and echidna chrX1-X4) were established in the MRCA (**Fig. 2B**) [23]. The lineage-specific sex chromosomes, i.e., platypus X4 and echidna X5, originated independently from two different ancestral autosomes (**Fig. 2B**) as initially reported by cross species *in situ* hybridization [23]. Specifically, MON28 is maintained as a single autosome chr27 in echidna but becomes chrX4 in platypus (**Fig. 2B**). MON15 remained as a single chromosome chr12 in platypus but was separated into the echidna chr12 and chrX5 (**Fig. 2B**).

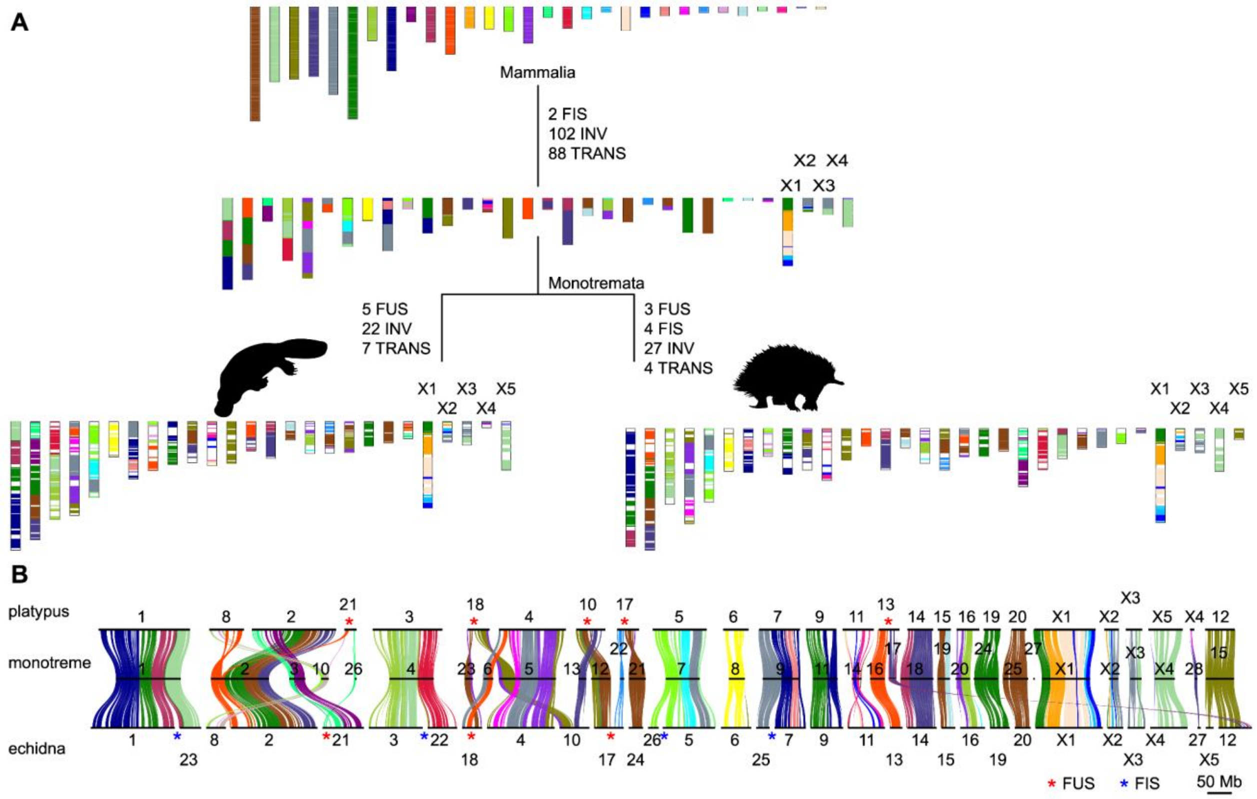

Fig. 2. Karyotype evolution of monotremes.

(A) 2n=64 ancestral karyotypes were inferred in the most recent common ancestor (MRCA) of monotremes, including 28 pairs of autosomes and four pairs of sex chromosomes, under 300 Kb resolution. Conserved blocks were color-coded with the chromosomal source in the mammalian ancestor. The length of the conserved blocks in the ancestors is taken as the length of the conserved blocks in human. Numbers of estimated rearrangements are shown for the evolution of monotreme MRCA to the extant species. FUS: fusion, INV: inversion, TRANS: translocation, FIS: fission. A more complete reconstruction of karyotype evolution is available in **supplementary fig. S8**, and a similar reconstruction under 500 Kb resolution is available in **supplementary fig. S9**. The yellow-colored chromosome is the one that evolved into therian X chromosome.

(B) Conserved block between the monotreme MRCA and the extant monotremes shows the chromosome rearrangement events during evolution. Alignment of the conserved blocks were

color-coded with the chromosomal source in the mammalian ancestor. Fusion (FUS) and fission (FIS) events are marked with red and blue asterisk, respectively.

Monotreme sex chromosomes have both shared and independently formed evolutionary strata

Our previous work suggested that the multiple sex chromosome system in platypus evolved from an ancestral chromosome ring structure, via a series of reciprocal translocations between proto sex-chromosomes and autosomes [10, 24] as well as chromosome fusions [9]. Among the five pairs of monotreme sex chromosomes, four are shared between platypus and echidna, but how each monotreme lineage evolved their distinct sex chromosome complex after they diverged from their common ancestor 55 MYA remains to be elucidated [9]. By projecting our ancestral karyotype reconstruction to the platypus and echidna sex chromosomes, we found that the monotreme ancestral sex chromosomes (i.e., echidna X1-X4 and platypus X1-X3 & X4) consist of homologous fragments from different ancestral chromosomes (**supplementary fig. S11a, supplementary tables S11 & S12**) [9]. Specifically, parts of each two neighboring sex chromosomes are homologous to two adjacent regions of the same ancestral chromosome (**supplementary fig. S11a**), forming the PARs and the sex differentiated regions (SDRs). This suggests that a high number of translocations occurred before the monotremes evolved their extant sex chromosome configuration. The species-specific sex chromosomes, i.e., platypus Y3X4 and echidna X5, originated from different mammalian ancestral chromosomes (MAMs) (**supplementary tables S11 & S12**). Consistent results could be confirmed by the projection using the chicken genome (**supplementary fig. S11b, supplementary table S13**).

In many species, sex chromosome evolution is characterized by stepwise recombination suppression, where would lead to the stratified pattern of different sequence divergence levels

between X and Y sex differentiated regions termed ‘evolutionary strata’ along the sex chromosome [10, 16]. Previously we inferred seven strata in the sex chromosome chain by X/Y gametologues and their phylogeny [9], but this could be impacted by the limited number of gametologue pairs and possible gene conversion between the pair [32]. Here with more gametologue pairs from the more complete echidna genome, we found that the pairwise dS values between gametologue pairs in the previously identified S0-S4 strata located on the X1-X4 chromosomes did not show significant differences (**supplementary fig. S12a**). Interestingly, among these X/Y gametologue pairs, over 80% of the Y gametologues are located on the one Y chromosome echidna Y3 or its homologous platypus Y5 [23] (**supplementary fig. S13**), respectively **(supplementary tables S14 to S16**). The X/Y sequence alignments also revealed that the echidna Y3 (or platypus Y5) exhibit the largest (>60%) aligned region on the X1, followed by smaller alignments with X2, X3 and X4 (or platypus X5) (**Fig. 3A, supplementary table S17**); in contrast, the other Ys are mostly homologous to their neighboring Xs (**supplementary table S17**). On the other hand, we have not found one X chromosome that exhibits as many alignable fragments to many Ys. Instead, when excluding echidna Y3 and platypus Y5, all X chromosomes are aligned most to their neighboring Ys (**supplementary table S17**). Such a pattern of “one Y to many X” can be achieved only via a series of autosome-X translocation (**Fig. 3B**) instead of autosome-Y translocation, which may produce the opposite “one X to many Y” result (**Fig. 3C**). Notably, we found that *AMHX* in platypus should locate near the end of chrX1 (**supplementary fig. S11**) and in the same syntenic region as in echidna (**Fig. 3A**), instead of our previous inference at the middle part of X1 [9].

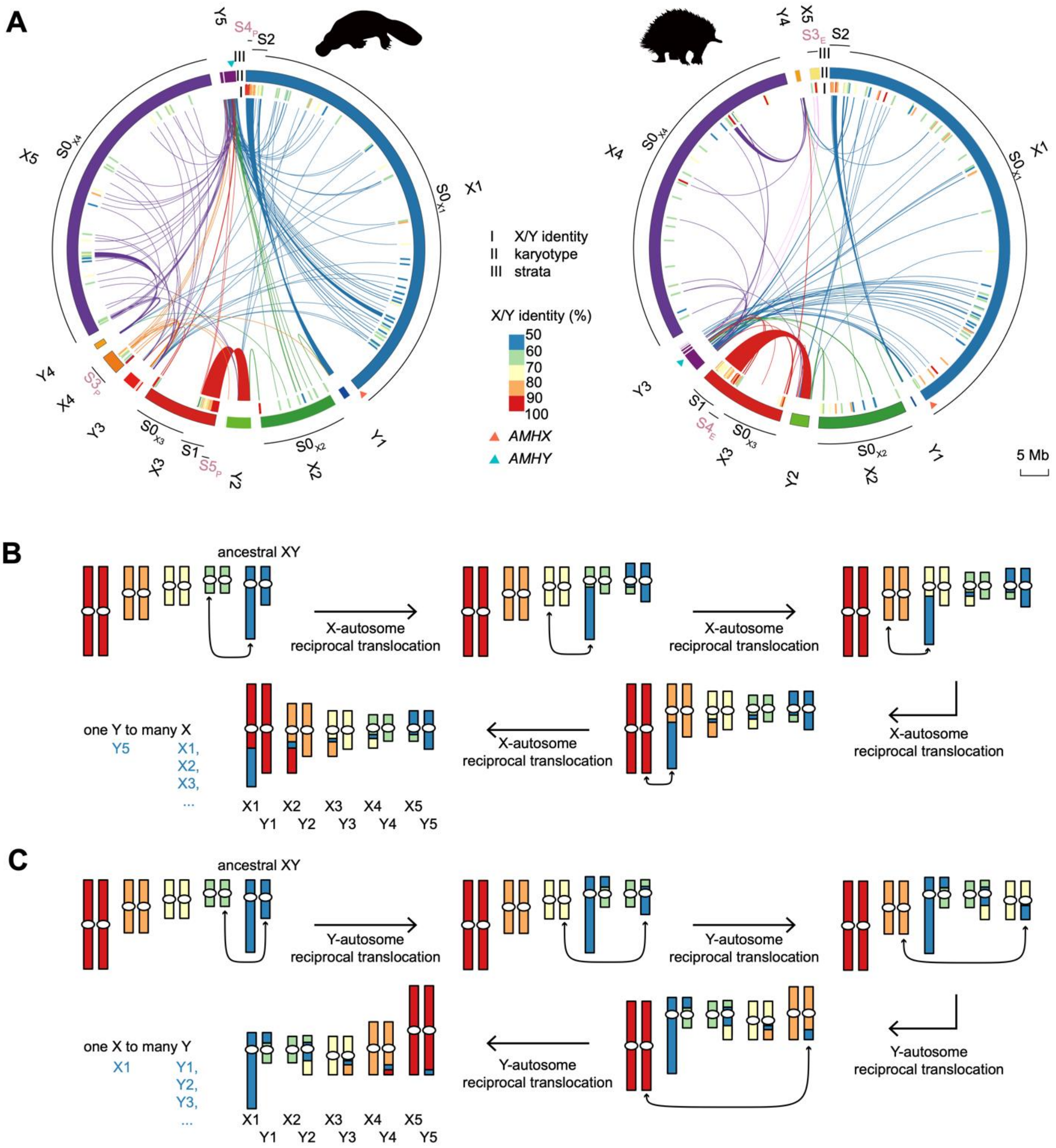

Fig 3. X/Y sequence alignment and the two possible reciprocal translocation scenario in multiple sex chromosome evolution.

(A) Tracks from inside out (I - III): X/Y identity, karyotype (PAR excluded), strata. The orthologous chromosome, echidna Y3 and platypus Y5, are homologous to multiple X chromosomes in both species including X1, X2 and X3 and echidna X4 (platypus X5). The

species-specific sex chromosome is homologous to the sex chromosome it paired with during meiosis. Four strata (S0-S3) are ancestral (black) while the younger four (S4-S6) evolved independently in the two lineages (brown). Only assigned X and Y are shown. Independent strata are marked with a subscript “P” or “E” indicating the strata evolved in platypus or echidna, respectively.

(B, C) Evolution of the sex chromosome chain by a series of reciprocal translocations between ancestral autosomes and X (B) or Y (C). (B) The reciprocal translocation between the ancestral X and the ancestral autosomes will distribute the ancestral X to the ancestral autosomes, resulting in “one Y to many X” homology relationship in the end. (C) The reciprocal translocation between the ancestral Y and the ancestral autosomes will distribute the ancestral Y to the ancestral autosomes, resulting in “one X to many Y” homology relationship in the end. Based on our observation in platypus and echidna, the translocation between autosomes and X is more possible for the evolution in monotreme sex chromosome evolution.

Both the X/Y divergence and X/Y homology pattern suggest an alternative monotreme sex chromosome evolution model contrary to our previous hypothesis that recombination suppression happened after reciprocal translocations. Instead, the recombination suppression might have already initiated on the ancestral X (X1) and Y (echidna Y3 or platypus Y5) in the monotreme ancestor to form the ancestral stratum S0. Subsequently a series of translocations between the non-recombining X and autosome occurred, producing the scattered homology between one ancestral Y and four ancestral X chromosomes (except for the echidna X5 and platypus X4), leaving similar dS levels of X/Y gametologues across different X chromosomes (**Fig. 4**). In addition, by distributing the ancestral non-recombining X to different chromosomes, the pairing Y chromosome can no longer recombine with the X-counterpart (e.g. during meiosis echidna Y3 only pairs with X3 and X4 but not X1 and X2), leading to the accumulation of deleterious mutations on the Y chromosomes. Moreover, such reciprocal translocations may

also initiate the recombination suppression between the neighboring sex chromosomes (e.g. X2-Y2), creating gametologues with younger ages and unlikely to be involved in sex determination. Under such a scenario, we proposed that there were at least six and five evolutionary strata in platypus and echidna, respectively, with the oldest four evolved ancestrally in the monotreme MRCA while the youngest three or two evolved independently in the two lineages (**Fig. 3A, supplementary figs. S12b, S14 & S15; supplementary tables S14 & S16**). The oldest stratum S0 were delineated to be distributed across all four ancestral X chromosomes (named by their extant residing chromosomes as echidna $S0_{X1}$-$S0_{X4}$ and similarly in platypus). According to the gametologue phylogeny while controlling for gene conversion (**supplementary fig. S14, supplementary table S18**), and that both X and Y are from different chromosomes, we considered S1 (X2-Y2) and S2 (X1-Y1) derived from different MAMs as different strata but formed in the monotreme ancestor. An additional translocation further occurred in echidna, leading to a synteny disruption between the two monotremes (see below).

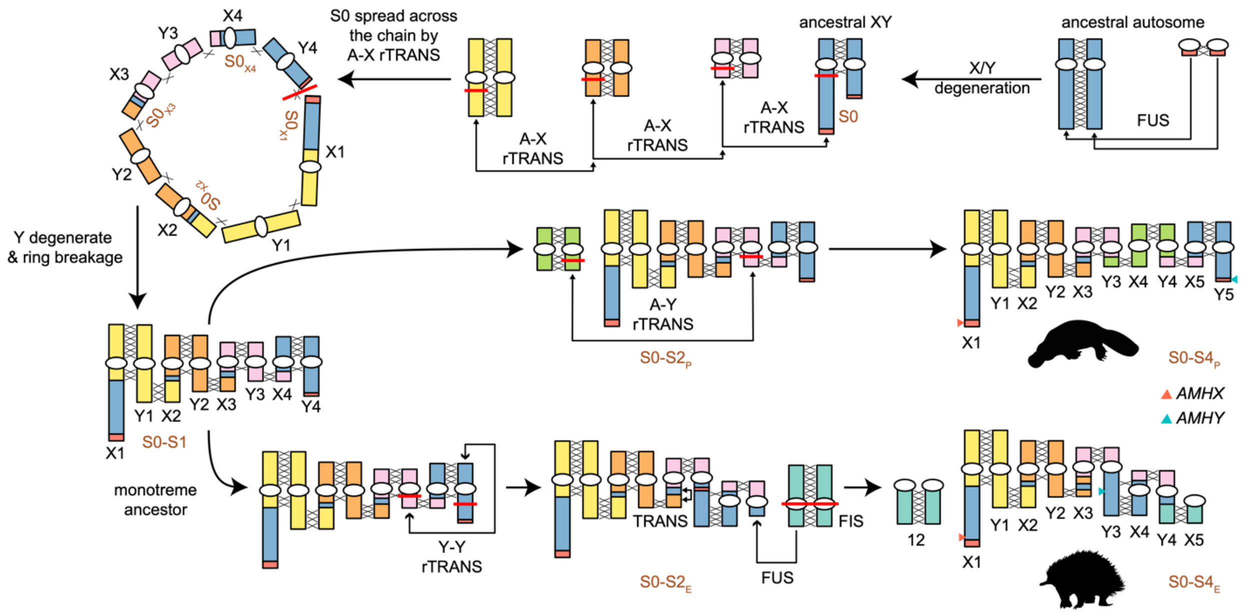

Figure 4. Model for the sex chromosome evolution in monotreme.

The evolution of the sex chromosome complex in monotreme MRCA involves both chromosome fusion and reciprocal translocation between ancestral autosomes or between ancestral autosomes vs. one pair of ancestral XY chromosomes and the oldest stratum S0 evolved. The translocation distributes the ancestral X segments and S0 into many ancestral autosomes, results in "one Y to multiple X" homology relationship and possibly forms a ring structure. The Y sequence degeneration further inhibits the pairing and breaks the ring into a chain. S1 and S2 later evolved in the monotreme ancestor and cause PAR erosion. The system then undergoes different evolutionary trajectories between platypus and echidna by recruiting different autosomes into the complex after they split. In platypus, a reciprocal translocation happened between autosome and ancestral Y3, and recruited the autosomes into X4 and part of Y3/Y4 and evolving into its independent stratum S_P. In echidna, a Y3-Y4 reciprocal translocation happens and alters the X/Y pairing order. The ancestral Y4 in echidna further experienced chromosome fusion with part of autosome sequences and formed $S3_E$. Additional translocation also happens in echidna X3 disrupting its synteny (including S1 and $S4_E$) with platypus. Recombination suppression further happened independently in platypus and echidna on X1 and X3 and formed the youngest two strata. The coordinates of the putative sex-determining gene *AMHX/Y* are also labelled in platypus and echidna. rTRANS: reciprocal translocation, TRANS: translocation, FUS: fusion, FIS: fission. A, autosome. The platypus silhouette is created by S. Werning and is reproduced under the Creative Commons Attribution 3.0 Unported license. Different ancestral chromosomes are filled with different colors.

Among three younger strata (S3-S5) that evolved independently in the two species, $S3_P$ (platypus S5) and $S3_E$ (echidna S5) are located on the species-specific X, i.e., platypus X4 and echidna X5, respectively [23, 25], though the support of independent evolution from gametologue phylogeny are ambiguous (**supplementary fig. S14c, supplementary table S18**). Previous studies and the above ancestral karyotype reconstruction showed that the species-

specific X chromosomes of these two species are homologous to an autosome in the other species, thus providing a unique model to study the lineage-specific genomic changes involved in the sex chromosome evolution. In echidna, 88.81% of the assembled X5 shows a similar sequencing depth between male and female (**supplementary fig. S16a**), which indicates this is a recently evolved X chromosome and only contains a small non-recombining region. Nine genes reside in the remaining 1.8 Mb X-Div on X5 (**supplementary fig. S17a**). Interestingly, an ~300 Kb inversion was identified between the X-Div region of echidna X5 and its orthologous region in platypus chr12, spanning one gene *TACR3* (**supplementary fig. S17a**). This inversion may have contributed to one of the recombination suppressions on echidna X5 (**supplementary fig. S17c**) and led to the degeneration of its Y counterpart. In human, *TACR3* resides on the autosome, encodes receptors for neurokinin B and is found to be associated with hypogonadotropic hypogonadism [33]. In both human and platypus, the gene is mainly expressed in somatic tissues, but in echidna the gene shows the highest (though not specific) expression in testis (**supplementary fig. S18**), suggesting recent adaptation for a testis-related function. The remaining X-Div on echidna X5 is homologous to a platypus scaffold (scaffold_344_arrow_ctg1) located on platypus X3 by our Hi-C analysis (**supplementary figs. S2 and S16a**), and only contains genes encoding olfactory receptors and vomeronasal receptors (**supplementary fig. S17a**). Thus, in addition to the previous FISH experiment showing that echidna X5 is mapped to the platypus chr12 [23], our observation here suggests that the evolution of echidna X5 may also involve some rearrangement with a part of the ancestral X3. We found longer X/Y alignment remained in the region homologous to platypus scaffold_344_arrow_ctg1 (5,239 bp, 0.95% of the X-Div) than that in the inversion region (1,000 bp, 0.25% of the X-Div), while the sequence divergence level is similar between the two regions (two-sided Wilcoxon rank-sum test, p = 0.8571). Thus, we hypothesized that echidna X5 first experienced an inversion on the X, then fused with the monotreme ancestral X3 sex chromosome. We also performed similar analysis to platypus X4 (**supplementary text**). In

contrast to echidna X5, platypus X4 did not undergo such inversion. The recombination suppression on X4 started at the chromosome end distant to the current PAR, and eroded to the current boundary (**supplementary fig. S17**).

Platypus' second youngest stratum $S4_P$ located in X1 where the orthologous region in echidna remains as PAR (**supplementary fig. S15**). The youngest platypus and echidna stratum $S5_P$ and $S4_E$ are located near the respective PAR boundary of the ancestral X3 with supports from various gametologues (**supplementary fig. S15, supplementary table S18**). Interestingly, besides an overall high-level of synteny between platypus and echidna of the ancestral Xs (**Fig. 1B**), we identified one translocation on X3 between the two species. Such translocation spans two strata, the ancestral S1 and $S5_P$ (or $S4_E$) **(Fig. 3A, supplementary fig. S19a**), with a length of at least 4.5 Mb and 29 protein-coding genes. This pattern, and our ancestral karyotype reconstruction (**supplementary tables S8 and S9**) and alignment with other mammals demonstrated that the translocation is more likely to happen specifically in echidna (**supplementary fig. S19b**).

Based on these observations and Dohm et al. [25] we also proposed a model to explain the evolution of the complex sex chromosome system in monotreme after platypus-echidna split (**Fig. 4**). After speciation, in platypus, a reciprocal translocation may happen between an autosome and the ancestral Y3, creating its X4-Y4 containing a new stratum $S3_P$. In echidna an ancestral Y3-Y4 translocation first happened to exchange the pairing relationship with X. This follows a chromosome fission of an ancestral autosome and a Y-autosome fusion to form the current chr12 and Y4, recruiting the extant X5 into the sex chromosome system similar to the case of neo-X evolution in *Drosophila miranda* [34] and creates its specific $S3_E$. The two youngest strata ($S4_P$, $S5_P$ and $S4_E$) further evolved independently in the two species. A translocation also happened on echidna X3, changing the genomic coordinate of two strata (S1

and $S4_E$). Based on X/Y sequence divergence, we estimate the ages of the evolutionary strata. The multiple sex chromosome started since the very first recombination suppression on the ancestral sex chromosome at approximately 80 million years ago (MYA) (**supplementary table S19**), follows by spreading of the ancestral X fragments spread across the complex via a series of X-autosome translocations. The species-specific X, platypus X4 and echidna X5, stops its recombination around 19 and 27 MYA, respectively (**supplementary table S19**).

The evolution of sex-linked ampliconic genes

One of the notable features of the sex chromosome is that some genes have undergone amplifications to produce highly identical (>99%) copies termed ampliconic genes (AGs) [35]. These genes have been observed to be organized as tandem arrays [36, 37], or inverted repeats described as palindromes [38]. Previous studies have revealed the existence of AGs in both X and Y chromosomes of therian and the Z chromosome in chicken [23, 38-42] as well as on the recently evolved X and Y chromosomes of *Drosophila miranda* [43]. However, to date only limited information about genome architecture is available for the Y chromosomes of the egg-laying mammals [10]. Utilizing the gene annotation from the long-read assemblies and the male sequencing depth information, in platypus and echidna, we found 10 and 5 X-linked AGs; and 12 and 11 Y-linked AGs, respectively (**supplementary tables S20 & S21**), in contrast with the large number of ampliconic genes in eutherian mammals and chicken [40, 44]. Our platypus and echidna Y-linked AG dataset each contains 3 and 4 types of newly reported Y-linked AGs (**supplementary tables S20 and S21**). However, these AG numbers might be underestimated because some may have been collapsed during the genome assembly. As found in human, great apes, mouse and chicken, in monotreme both X and Y AGs were found to be predominantly expressed in testis (**supplementary table S22**), consistent with the previous finding from a small subset of these families [10]. Interestingly, only a few of them were also

testis-specific expressed in human, suggesting that most of the genes were masculinized in monotremes only after it become sex-linked (**supplementary table S23**).

Similar to the observation in the comparison between human and mouse ampliconic genes, in monotremes most ampliconic genes are independently amplified after their divergence about 55 million years [9] (**Fig. 5A & B**). Only one X-linked (*DYNLRB2X*s) and three Y-linked (*SYCP3Y*s, *RNF17Y*s and *MED26Y*s) AGs was shared between echidna and platypus. As expected, all these shared X-linked and Y-linked AGs are located on the ancestral sex chromosomes shared by platypus and echidna. The AGs shared between the two monotremes should have evolved in their common ancestor and are likely to be important for both species and have been maintained through the degeneration process of the Y chromosomes. For example, we found the Y-linked AG *SYCP3Y* are amplified in both platypus and echidna. *SYCP3Y*s are thought to evolve from an autosomal copy *SYCP3* [10] which encodes protein to form the synaptonemal complex at meiotic prophase I [45]. In this study we further confirmed that such duplications from autosomes were ancestral in monotreme MRCA at the early stage of sex chromosome evolution (*SYCP3-SYCP3Y* dS ~0.7, **supplementary table S24, supplementary fig. S20**). Interestingly, monotreme *SYCP3Y*s share higher sequence identity with *SYCP3* in other mammals than its autosomal paralog *SYCP3,* and harbors a newly evolved motif that enables self-association and normal function in synaptonemal complex [46]. Both *SYCP3Y*s are expressed predominantly in testis (**Fig. 5C; supplementary tables S20 and S21**). Many proteins that act in meiotic and post-meiotic cells are highly transcribed in pre-meiotic cells. Analysis of the platypus spermatogenesis single-nucleus RNA-seq data [47] revealed that *SYCP3Y*s mainly expressed in spermatocytes, which are in the meiosis I stage where the sex chromosomes are paired and chained [48] (**supplementary fig. S21**). It may be that the amplified *SYCP3Y* genes evolved a male specific function at meiosis associated with the formation of the complex sex chromosome chain. We

hypothesized that these amplifications may be due to the need for the unique pairing and segregation of the multiple sex chromosomes during male meiosis [21, 22].

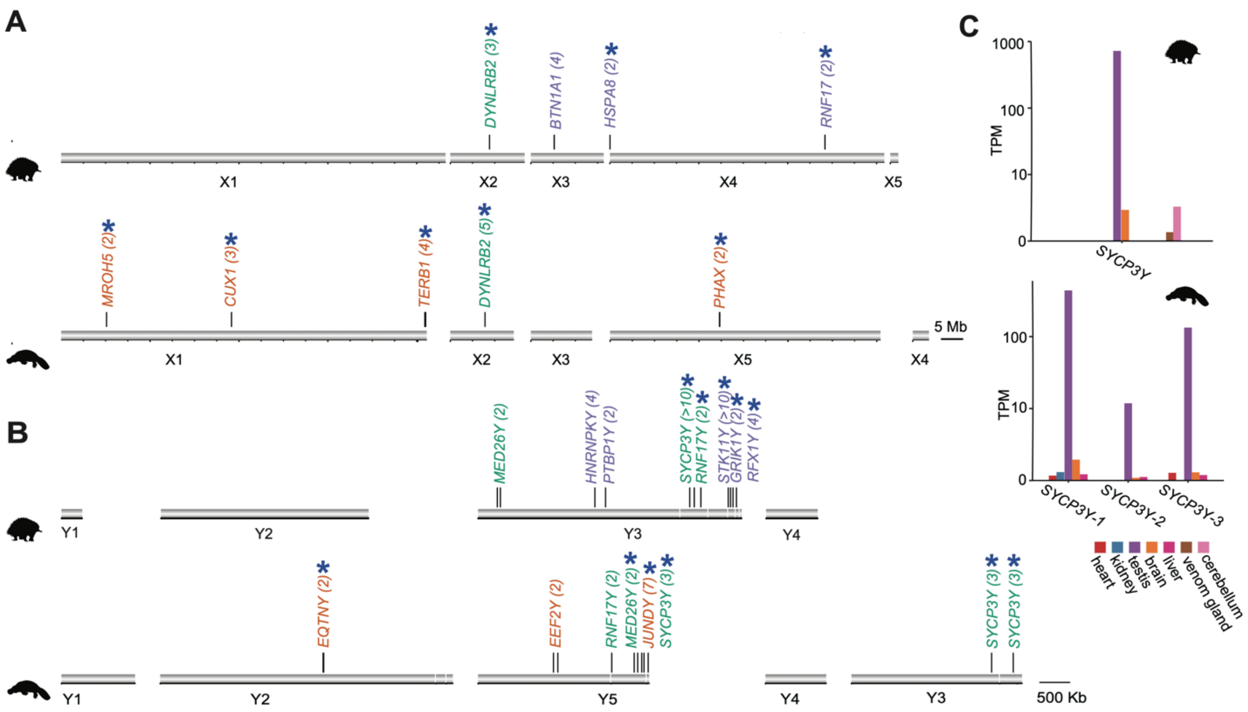

Figure 5. Ampliconic genes in monotremes.

(A) Distribution of ampliconic genes in echidna (purple) and platypus (orange) X chromosomes. Green, genes that are ampliconic in both species. The estimated copy number for each ampliconic gene is shown in parentheses. Ampliconic genes with testis-specific expression are marked by asterisks. Homologous chromosomes are shown in the same column.

(B) Distribution of ampliconic genes in echidna (purple) and platypus (orange) Y chromosomes. Green, genes that are ampliconic in both species. The estimated copy number for each ampliconic gene is shown in parentheses. Ampliconic genes with testis-specific expression are marked by asterisks. Homologous chromosomes are shown in the same column.

(C) Testis specific expression pattern of ampliconic genes *SYCP3Y* in both echidna and platypus.

Discussion

A high-quality genome is important for the understanding of evolution, particularly the sex chromosome since it is difficult to sequence and assemble [8]. Analysis on monotreme genomes has revolutionized our understanding of mammalian sex chromosome evolution but we still lack a good understanding of how the complex monotreme sex chromosome system evolved. Here we presented an improved chromosome-level short-beaked echidna assembly constructed from the latest sequencing technologies. This enables us to reconstruct the monotreme ancestral karyotype and investigate the evolutionary trajectory of monotreme sex chromosomes in unprecedented detail. With the newly improved echidna assembly, we explored the different evolution trajectories of the species specific and on the ancestral sex chromosome in greater detail than was possible before. The discovery of homologies for echidna Y3 and platypus Y5 with multiple X chromosomes, while other Ys are homologous only to their neighboring Xs supports the idea of reciprocal translocations between the ancestral autosomes and the ancestral X instead of the Y [24, 49]. In addition, no significant difference in dS (or X/Y identity) are found among the gametologues on the four ancestral X chromosomes (**supplementary fig. S12**), suggesting that the sex chromosome has already diverged ancestrally and the ancestral evolutionary stratum was spread across the chain via a series of autosome-X reciprocal translocations (**Fig. 4**).

Multiple sex chromosome systems have been found in a variety of species including therians, avians, frogs, insects and plants, forming chain or ring like structures [24, 50-52]. These systems are typically composed of three (trivalent) or four (quadrivalent) chromosomes resulting from one or two translocation events. In contrast, the monotreme sex chromosomes complex evolved over a longer period of time is more complex and evolved over more than 80 million years with recent changes after platypus and echidnas diverged. Such a complex requires precise formation of a

chain at meiosis and alternate segregation. Indeed, previous studies have shown that the sex chromosome chain is assembled in an order starting from Y5 and ending with X1 during meiosis [53]. In addition, dynamic cohesin was observed in platypus prophase I, where the protein differentially loaded at the paired and unpaired regions [54]. Here, we found gene family expansion signals, potentially arising from the evolution of the multiple sex chromosome system. We confirmed *SYCP3Y* amplification in platypus [46], and also found such amplification in echidna, suggesting that the gene expansion is ancestral and may associate with the evolution of the sex chromosome complex or play a role in its organization. Interestingly, pairwise dS values between *SYCP3* and *SYCP3Y* are around 0.7, which falls within the range of the pairwise dS value of the S0 gametologues (**supplementary table S15**). This suggests that the duplication of *SYCP3Y* from *SYCP3* likely occurred around the same time as the early divergence of the ancestral X and Y chromosomes, predating the reciprocal translocation events. It is possible that the pre-adaptation by *SYCP3Y* acquisition facilitated the reciprocal translocations in sex chromosome evolution by fulfilling the requirement for alternate segregation of the sex chromosome complex. Ampliconic genes have been discovered on the sex chromosomes of many other species such as mammals and fruit flies [40, 43]. Several evolutionary processes, including male beneficial mutation and meiotic conflict, have been proposed as the cause for this genomic event [43]. In monotremes, the special need for pairing and segregation of the multiple sex chromosome system in male, may provide additional evolutionary drive to gene amplification.

In conclusion, our results provide a comprehensive evolutionary history of monotreme sex chromosomes, and uncovered novel aspects of its genetic composition including the sex-linked gene amplification. Future work still needs to uncover the mechanisms of alternative segregation and sex specific function of genes in particular those that have undergone ampliconic expansion. Expression of those genes at specific stages in spermatogenesis is indicative of reproductive

function. This new and more complete echidna genome will continue to refine our understanding of sex chromosome evolution, organization and function in monotremes and other mammals.

Methods

Sample collection, genome sequencing, assembling and sex-linked sequence identification

Echidna sample Emale12 were collected under AEC permits S-492006, S-032-2008 and S-2011-146 at Upper Barnard River (New South Wales, Australia) during the breeding season and the muscle sample was frozen into liquid nitrogen and was used for PacBio sequencing. Other echidna genomic sequencing data, including 10X, Bionano and Hi-C, were obtained from Zhou et al. [9]. The genome was assembled following the VGP assembly pipeline v.1.6. Genome completeness was evaluated using BUSCO (v5.7.1) (RRID:SCR_015008) [55] and compleasm (v0.2.6) [56], a faster and more accurate reimplementation of BUSCO, with mammalia_odb10 as the database. Male and female Illumina short reads were obtained from NCBI (male: PRJNA576333, female: PRJNA202404) and mapped to the genome using BWA MEM (v0.7.17) [57]. Sex-linked sequences were identified with the same procedure described in Zhou et al. [9]. Briefly, male and female Illumina short reads were mapped to the new echidna assembly using BWA MEM with default parameters. Coverage was extracted with samtools (v1.9) (RRID:SCR_002105) [58], normalized by the peak coverage, and was then calculated in 5 kb windows with bedtools (v2.29.2) (RRID:SCR_006646) [59]. Scaffolds (>10 kb) of over 60% of windows with normalized F/M coverage ratio between 1.5 and 2.5 were identified as X-linked,

and between 0.0 and 0.3 as Y-linked. Coverage of candidate X- and Y-linked scaffolds was also visualized with ggplot2 (v3.3.5) (RRID:SCR_014601) and manually examined to delineate the PAR within each scaffold. In addition, we further remove possible false positives of the unplaced sex-linked scaffolds, based on the interaction strength under 100 Kb resolution obtained from Hi-C, with the same method described in Yang et al. [60]. Briefly, interaction strength between each candidate unplaced sex-linked scaffold and the assigned autosome and X/Y were compared. We only kept the unplaced sex-linked scaffolds if its interaction with the assigned X/Y was significantly higher than that with the assigned autosome (one-sided Wilcoxon rank-sum test). We also visualized the Hi-C maps of each of these scaffolds and their assigned chromosomes with hicexplorer package (v3.7.2) (RRID:SCR_022111) [61] and manually confirmed the results with the maps. The estimated sex chromosome sizes were inferred using the same method as described in Rhie et al. [8]. PAR were included for both X and Y completeness evaluation. For example, X1Y1 PAR and Y1X2 PAR were summed with Y1 Y-Div and compared with the expected Y1 size to evaluate the completeness of Y1. We also collected echidna male specific transcripts from Cortez et al. [10] to evaluate the completeness of the Y gene dataset. Transcript sequences were mapped to the reference genome with BLAT (v319) (RRID:SCR_011919) [62] with parameter “-fine”. Only mapping results to Y-Div were kept.

Examination of the Illumina-based assembly gap filling status in PacBio-based assembly

We used a similar method as Bickhart et al. [63] to identify the gap filling status in the PacBio assembly. Briefly, 500 bp fragments upstream and downstream of each gap in the Illumina assembly were extracted and then aligned back to PacBio assembly by BWA MEM (RRID:SCR_010910) [57]. If a gap is too close (<200 bp) to the end of the scaffold, or its size <5 bp, the gap is excluded in further analysis. If both fragments aligned successfully (aligning

rate > 70%) to the same scaffold in the PacBio assembly and the intervening sequence of PacBio assembly did not contain any ambiguous base (N), the gap was considered closed. If the two fragments were aligned to different scaffolds, the gap was considered a trans-scaffold break. If one or both fragments did not align to PacBio assembly, or the intervening sequence contained the ambiguous bases, the gap was considered open.

Pseudoautosomal regions (PARs) identification with Hi-C

Above method of sex-linked sequence identification can only identify PAR which is assembled with X-Div or Y-Div. We found that two pseudoautosomal regions (PAR), i.e., X3Y3 and Y3X4 PAR, cannot be identified based on the above depth method from the echidna genome. Under the general Hi-C assumption that the intrachromosomal interaction is larger than the interchromosomal interaction [64], we therefore used the Hi-C interaction matrix to identify PAR sequences from the unplaced scaffolds. We assume that, if an unplaced scaffold is X3Y3 (or Y3X4) PAR, its interaction with X3 and Y3 (or Y3 and X4) should be stronger than the interaction with other sex chromosomes and autosomes. Thus, for each unplaced scaffold, we extracted its Hi-C interaction under 100 Kb resolution with X3, Y3 (or Y3, X4), and compared the dataset with the Hi-C interaction with each other anchored sex chromosomes as well as autosomes. If the unplaced scaffold has significantly higher Hi-C interaction with X3 and Y3 (or Y3 and X4) than the Hi-C interaction with every other anchored chromosome under one-sided Wilcoxon rank-sum test, we consider it as the X3Y3 (or Y3X4) PAR. We also tried this method in platypus to identify X4Y4 PAR, but no unplaced scaffolds showed significantly higher interaction with the anchored X4 and Y4 when compared to other chromosomes.

Comparison between the platypus and echidna assembly

We used lastz (v1.04.00) (RRID:SCR_018556) [65] to align the new echidna assembly to the platypus assembly with parameter set “--hspthresh=4500 --gap=600,150 --ydrop=15000 --notransition”. Only alignments over 10 Kb were kept for plotting in Fig. 1. Dotplot was generated with the custom python script. To generate the pairwise alignment between sex-linked sequences, we also performed lastz alignment between the two assemblies, with the parameter set the same as mentioned above and a matrix for closely related species. We confirmed the structural variants between the two genomes with PacBio, 10X-linked reads and Hi-C data. Since the homology between echidna and platypus are not available for all chromosomes [23], in this study we assigned scaffolds to chromosomes based on the mashmap alignment between the two species, except for the sex chromosome whose nomenclature is based on Rens et al. [23] (**supplementary table S3**).

Ancestral karyotype reconstruction

We utilized the genomic information to reconstruct the ancestral karyotype of monotremes with a similar method as in Zhou et al. [9]. The *Ornithorhynchus anatinus* genome (GCF_004115215.2) was used as reference and genomes of *Bos taurus* (GCF_002263795.1), *Choloepus didactylus* (GCF_015220235.1), *Gallus gallus* (GCF_016699485.2), *Homo sapiens* (GCA_000001405.28), *Monodelphis domestica* (GCA_000002295.1), *Podarcis muralis* (GCA_004329235.1), *Sarcophilus harrisii* (GCA_902635505.1), *Tachyglossus aculeatus* and *Trachemys scripta elegans* (GCF_013100865.1) was aligned to the reference using lastz with parameter set “--step=19 --hspthresh=2200 --inner=2000 --ydrop=3400 --gappedthresh=10000” and a matrix for distantly related species. Genomes were softmasked before running lastZ. Conserved segments among the species was extracted from the NET result with DESCHRAMBLER (git commit 28686dda39144f9d8223dce663aadf0621002643) [29] under 300 Kb resolution, with the tree

was obtained from Timetree [66]. We required conserved segments to be uniquely and universally presented in all mammals, but allowed segments missing or duplicated in the reptilian outgroups. Ancestral karyotype reconstruction was performed with ANGES (v1.01) [67] for all nodes after mammal radiation, and we further curated the results according to previous reconstruction by FISH or bioinformatic method [9, 14, 27-29]. We also incorporated pairiwise gene synteny information inferred from MCScanX (RRID:SCR_022067) [68] to link the contiguous ancestral regions (CARs) at monotreme MRCA (**supplementary table S10**). The length of the ancestral chromosome was based on the length of the conserved blocks in human. We also performed a reconstruction under 500 Kb resolution. The overall results were similar, except that there was no conserved segment for platypus chrX4 and echidna chr27 due to the 500 Kb threshold in monotreme ancestral karyotype reconstruction, thus MON28 was not available in the result (**supplementary table S12, supplementary fig. S9**). Manual curation was performed to link PAR with X/Y-Div that were separately assembled in the genome. Rearrangement events from monotreme MRCA to extant species were then inferred with GRIMM (v2.1) [69].

Sex chromosome evolution

Chromosome painting with chicken genome sequence

To obtain the orthologous information between monotremes sex chromosomes and chicken genome, we aligned the chicken genome (GCF_016699485.2) to each of the monotreme genome with lastZ under parameter set “--step=19 --hspthresh=2200 --inner=2000 --ydrop=3400 --gappedthresh=10000” and a matrix for distantly related species. We only kept alignment ≥ 100 Kb. Gaps between alignment were filled with adjacent alignment results and visualized with ggplot2 (v3.3.6). Since each PAR was assembled in one copy in the haploid genome, we

duplicated the PAR alignments and placed each to X and Y chromosomes for visualization. Y-linked scaffolds were ordered based on its length during visualization.

Confirmation of platypus *AMHX* genomic coordinate

Platypus *AMHX* is not assembled in the genome (GCF_004115215.2) used in this study. To locate the position of *AMHX* on chrX1, we extracted the *AMHX* located scaffold (Contig22983) from another platypus genome (OANA5), and combined it with GCF_004115215.2 to obtain a more complete assembly. Platypus Hi-C reads were aligned to this more complete genome with juicer (v1.6) and a hic file was generated. We split chrX1 into 100 Kb non-overlapping windows, and calculated the sum of the interaction strength (normalized with SCALE method) of each window under 10 Kb with Contig22983 using straw (v0.08). Juicebox (v1.11.08) (RRID:SCR_021172) was used for Hi-C matrix visualization.

Strata

We used the similar method in Zhou et al. [9] to identify the strata in echidna and platypus sex chromosomes. Briefly, repeat annotation was obtained from NCBI; we performed additional repeat annotation using the Tandem Repeat Finder (v4.09) (RRID:SCR_022193) [70] and RepeatMasker (v4.1.0) [71] where the library was generated based on the respective monotreme genome with RepeatModeler (v1.0.8) (RRID:SCR_015027). Repeat in Y-Div and X-Div were then N-masked, aligned with lastZ, and the maf results were used to calculate X/Y identity in 1 Kb windows. We also performed additional lastZ alignment between Y-Div and other genomic regions (autosome + X-Div + PAR). X/Y alignment would be filtered out if the Y segments can be better aligned to autosome/PAR, defined as having higher identity and longer alignment to autosome/PAR than to X-Div. Circos (v0.69-9) [72] were used to visualize X/Y alignment and sequence identity. X/Y gametolog pairs were identified by BLASTP the Y gene protein sequences to all X+autosome gene protein sequences. Only Y gene best hit to X genes

were kept and we further examined the gene name to confirm their homology. X/Y gametolog CDS alignment was built PRANK (v170427) [73] and dS was calculated using PAML codeml (v4.8) [74]. To confirm if the gametologue pairs originated ancestrally or independently in the two species, we obtained the protein sequences of the X and Y gametologues, performed multiple sequence alignment by PRANK, converted back to CDS alignment and then constructed each phylogeny tree by RAxML (v8.2.4) (RRID:SCR_006086) [75] with parameters "-f a -x 12345 -p 12345 -# 100 -m PROTGAMMALGX". Geneconv (1.81a) [76] was used to detect gene conversion signal from the alignment.

Species specific X evolution

The platypus and echidna lastZ result generated above was used here to obtain the alignment of the Xs between the two species. Gene distribution on the region was visualized with pyGenomeTracks (v3.7) [77]. N-masked X sequences were aligned to N-masked Y with lastZ under parameter set "--step=19 --hspthresh=2200 --inner=2000 --ydrop=3400 --gappedthresh=10000" and a matrix for distantly related species. We further filtered the alignment to remove the redundancy on the X, and on the basis of the 'net' and 'maf' results, the identity of each alignment block was calculated in 1 Kb non-overlapped windows. X/Y identity on different regions of echidna X5 and platypus X4 was classified according to the X alignment to the other species, and we performed a one-sided Wilcoxon rank-sum test if there's significant difference between the two regions. X/Y alignment was also visualized with circos (RRID:SCR_011798) [72], color-coded according to the Xs.

Age calculation of the monotreme strata

We used a similar method as Zhou et al. [15] to infer the age of each stratum. Since the mutation rates of male and female are different, the rate of XY sequence divergence is not the same as the rate of divergence of an autosomal duplication. However, they can be connected by

the male mutation rate α, which is the ratio of the male and female mutation rates. Assuming the female mutation rate is μ_f, the evolutionary rate of different chromosomes are:

A: $\frac{\alpha+1}{2}\mu_f$

X: $\frac{2+\alpha}{3}\mu_f$

Y: $\alpha\mu_f$

The divergence rate of autosome and XY are:

Autosome: $\frac{\alpha+1}{2}\mu_f + \frac{\alpha+1}{2}\mu_f = (1+\alpha)\mu_f$

XY: $\frac{2+\alpha}{3}\mu_f + \alpha\mu_f = \frac{2+4\alpha}{3}\mu_f$

Thus as Ross et al. [41], the ratio of rates of XY and autosome sequence is:

$$\frac{2+4\alpha}{3}\mu_f/(1+\alpha)\mu_f = \frac{2+4\alpha}{3+3\alpha}$$

We took the platypus autosomal divergence rate μ_{AA}, i.e., the mutation rate, 7×10^{-9}/site/year from Martin et al. [78], and the average male mutation bias $\alpha = 2.95$ estimated by Link et al. [79]. The platypus XY divergence rate μ_{XY} is thus 8.15×10^{-9}/site/year.

Assuming the molecular clock, the age of each stratum T can be calculated as $T = div/\mu_{XY}$, where the divergence between X and Y div was inferred based on the pairwise X/Y lastZ alignments generated above. We extracted all alignments of each stratum, removed alignments that fell in coding regions or repetitive sequences identified by RepeatMasker and Tandem Repeats Finder (v4.09) [70], and concatenated them into one single sequence alignment. We only used X-Y3/Y5 alignment for the calculation of S0. Divergence was estimated with baseml in PAML package (v4.8) (RRID:SCR_014932) [74] under JC69 model, and the 95% confidence interval was estimated after 1000 bootstraps. Divergence time of each stratum was calculated for each monotremes, and for the ancestral shared strata, we took the divergence time calculated from the larger alignment of the two monotremes in the main text.

Ampliconic region analysis

We mainly followed Makova et al. [80] to identify the ampliconic region by three methods, lastz, blastn and sequencing depth. To detect palindrome (≥98% identity, arm length ≥ 8 Kb, spacer ≤ 500 Kb), we first performed lastz alignment with parameter set "--self --format=general:name1,zstart1,end1,name2,strand2,zstart2+,end2+,id%,cigarx" and palindrover obtained from were used for palindrome detection. We further required the repeat content in candidate palindrome to be < 80%. Ampliconic region arranged in array were detected with the BLASTN method. Basically, the X-linked (or Y-linked) sequences were repeat-masked and split into 5 Kb windows with 2 Kb overlaps. We BLASTNed the sequences to itselft and only alignments with > 50% aligning rate and >99% identity were kept. We further merged the segments and required merged length ≥ 10 Kb. We also considered depth information to identify ampliconic regions since the ampliconic regions might have collapsed during assembling. Briefly, we mapped male re-sequencing reads to the genome with BWA MEM, and calculated the mean sequencing depth of each 5 Kb window after correcting with GC content with deepTools (v3.5.1) (RRID:SCR_016366) [81]. If the corrected sequencing depth of a nonPARX/Y window was larger or equal to that of the autosomes, the window would be considered as a candidate ampliconic region. We required the repeat content in candidate ampliconic region identified by depth to be < 80%. Ampliconic regions of the three methods were then merged with bedtools (v2.29.2) to obtain the final ampliconic region set. Genes with >80% of the length overlapping with the ampliconic regions were considered as ampliconic genes. Olfactory receptor and vomeronasal receptor genes were excluded since they were found amplified in the whole genome and were not specifically sex-linked [9].

RNA-seq data of platypus and echidna was obtained from NCBI with accession code SRP000120, SRP102989, SRP233233 and SRP027593. Expression level as transcripts-per-million (TPM) was estimated with Kallisto (v0.46.1) [82] with parameters '--bias'. Expression were normalized with DESeq2 (v1.31.16) (RRID:SCR_015687) [83] and the gene expression tissue specificity was quantified as 'tau' following the formula in Yanai et al. [84]. The expression

profile of AGs in snRNA-seq data of spermatogenesis were obtained from [47]. Human expression data was obtained from GTEx (RRID:SCR_013042) and the tissue specificity index tau was calculated with the same approach described above.

Additional Files

Additional File 1: supplementary text & supplementary figures S1-S21

Additional File 2: supplementary tables S1-S24.

Abbreviations

BUSCO: Benchmarking Universal Single-Copy Orthologs; PAR: pseudoautosomal region; SDR: sex-differentiated region; MRCA: most recent common ancestor; MON: monotreme ancestral chromosome; AG: ampliconic gene.

Acknowledgements

We thank BGI-Research and China National GeneBank for the computational resources in our analysis.

Author Contributions

G.Z. conceived the project. F.G., E.D.J., O.F., involved in sample collection, extraction and sequencing. G.G., S.P., A.R., A.M.P., K.H., Y.Z, J.J. performed genome assembling and curation. Y.Z., J.J., X.L. performed the evolutionary analyses. G.Z., Q.Z. supervised the project. Y.Z., G.Z., Q.Z., F.G., J.J., wrote the manuscript with input from all the authors.

Funding

This work was supported by the New Cornerstone Science Foundation through the XPLORER PRIZE and Kunpeng Program to G.Z., Young Elite Scientists Sponsorship Program by CAST (2023QNRC001) to Y.Z, and Intramural Research Program of the National Human Genome Research Institute, National Institutes of Health (A.R. and A.M.P.).

Data Availability

The genomic data generated in this study have been submitted to the NCBI BioProject database under accession number PRJNA1191144. The genome assembly is available at NCBI under BioProject PRJNA607237. All additional supporting data are available in the *GigaScience* repository, GigaDB [85].

Competing interests

The authors declare that they have no competing interests.

References

1. Damas J, Corbo M and Lewin H. Vertebrate chromosome evolution. Annual Review of Animal Biosciences. 2021;9:1-27. DOI: 10.1146/annurev-animal-020518-114924
2. Ferguson-Smith MA and Trifonov V. Mammalian karyotype evolution. Nature Reviews Genetics. 2007;8 12:950-62.
3. Guerrero RF and Kirkpatrick M. Local adaptation and the evolution of chromosome fusions. Evolution. 2014;68 10:2747-56.
4. Rieseberg L. Box 1. chromosomal rearrangements and meiosis. Trends in Ecology & Evolution. 2001;7 16:351-8. DOI: 10.1016/s0169-5347(01)02187-5
5. Yin Y, Fan H, Zhou B, Hu Y, Fan G, Wang J, et al. Molecular mechanisms and topological consequences of drastic chromosomal rearrangements of muntjac deer. Nature Communications. 2021;12 1:6858.
6. Dussex N, Van Der Valk T, Morales HE, Wheat CW, Díez-del-Molino D, Von Seth J, et al. Population genomics of the critically endangered kākāpō. Cell Genomics. 2021;1 1.
7. Jebb D, Huang Z, Pippel M, Hughes GM, Lavrichenko K, Devanna P, et al. Six reference-quality genomes reveal evolution of bat adaptations. Nature. 2020;583 7817:578-84.
8. Rhie A, McCarthy SA, Fedrigo O, Damas J, Formenti G, Koren S, et al. Towards complete and error-free genome assemblies of all vertebrate species. Nature. 2021;592 7856:737-46.
9. Zhou Y, Shearwin-Whyatt L, Li J, Song Z, Hayakawa T, Stevens D, et al. Platypus and echidna genomes reveal mammalian biology and evolution. Nature. 2021;592 7856:756-62.
10. Cortez D, Marin R, Toledo-Flores D, Froidevaux L, Liechti A, Waters PD, et al. Origins and functional evolution of Y chromosomes across mammals. Nature. 2014;508 7497:488-93.
11. Wesley C. Warren, LaDeana W. Hillier, A. J, Marshall Graves, Ewan Birney and Ponting CP. Genome analysis of the platypus reveals unique signatures of evolution. Nature. 2008;453 7192:175-83.
12. Deakin J, Graves J, Rens WJC and Research G. The evolution of marsupial and monotreme chromosomes. Cytogenetic and Genome Research. 2012;137 2-4:113-29.
13. McMillan D, Miethke P, Alsop AE, Rens W, O'Brien P, Trifonov V, et al. Characterizing the chromosomes of the platypus (Ornithorhynchus anatinus). Chromosome Research. 2007;15:961-74.
14. Ruiz-Herrera A, Farré M and Robinson T. Molecular cytogenetic and genomic insights into chromosomal evolution. Heredity. 2012;108 1:28-36.
15. Zhou Q, Zhang J, Bachtrog D, An N, Huang Q, Jarvis ED, et al. Complex evolutionary trajectories of sex chromosomes across bird taxa. Science. 2014;346 6215:1246338.
16. Lahn BT and Page DC. Four evolutionary strata on the human X chromosome. Science1999;286 5441:964-7. DOI: 10.1126/science.286.5441.964
17. Kitano J, Ross JA, Mori S, Kume M, Jones FC, Chan YF, et al. A role for a neo-sex chromosome in stickleback speciation. Nature. 2009;461 7267:1079-83.
18. Charlesworth D and Charlesworth B. Sex differences in fitness and selection for centric fusions between sex-chromosomes and autosomes. Genetics Research. 1980;35 2:205-14.
19. Ashley T. X-Autosome translocations, meiotic synapsis, chromosome evolution and speciation. Cytogenetic and genome research. 2002;96 1-4:33-9.
20. Eicher EM. X-autosome translocations in the mouse: total inactivation versus partial inactivation of the X chromosome. Advances in genetics. 1970;15:175-259.

21. Grützner F, Rens W, Tsend-Ayush E, El-Mogharbel N, O'Brien PC, Jones RC, et al. In the platypus a meiotic chain of ten sex chromosomes shares genes with the bird Z and mammal X chromosomes. Nature. 2004;432 7019:913-7.
22. Rens W, Grützner F, O'brien PC, Fairclough H, Graves JA and Ferguson-Smith M. Resolution and evolution of the duck-billed platypus karyotype with an X1Y1X2Y2X3Y3X4Y4X5Y5 male sex chromosome constitution. Proceedings of the National Academy of Sciences. 2004;101 46:16257-61.
23. Rens W, O'Brien PC, Grützner F, Clarke O, Graphodatskaya D, Tsend-Ayush E, et al. The multiple sex chromosomes of platypus and echidna are not completely identical and several share homology with the avian Z. Genome Biol. 2007;8:1-21.
24. Gruetzner F, Ashley T, Rowell DM and Marshall Graves. How did the platypus get its sex chromosome chain? A comparison of meiotic multiples and sex chromosomes in plants and animals. Chromosoma. 2006;115:75-88.
25. Dohm JC, Tsend-Ayush E, Reinhardt R, Grützner F and Himmelbauer H. Disruption and pseudoautosomal localization of the major histocompatibility complex in monotremes. Genome biology. 2007;8:1-16.
26. Wrigley JM and Graves J. Karyotypic conservation in the mammalian order Monotremata (subclass Prototheria). Chromosoma. 1988;96 3:231-47.
27. Deakin JE, Delbridge ML, Koina E, Harley N, Alsop AE, Wang C, et al. Reconstruction of the ancestral marsupial karyotype from comparative gene maps. BMC Evolutionary Biology 2013;13 1:1-15.
28. Froenicke L. Origins of primate chromosomes–as delineated by Zoo-FISH and alignments of human and mouse draft genome sequences. Cytogenetic and genome research. 2004;108 1-3:122-38. DOI: 10.1159/000080810
29. Kim J, Farré M, Auvil L, Capitanu B, Larkin DM, Ma J and Lewin H. Reconstruction and evolutionary history of eutherian chromosomes. Proceedings of the National Academy of Sciences. 2017;114 27:E5379-E88.
30. Uno Y, Nishida C, Tarui H, Ishishita S, Takagi C, Nishimura O, et al. Inference of the protokaryotypes of amniotes and tetrapods and the evolutionary processes of microchromosomes from comparative gene mapping. PloS one. 2012;7 12:e53027.
31. Waters PD, Patel HR, Ruiz-Herrera A, Álvarez-González L, Lister NC, Simakov O, et al. Microchromosomes are building blocks of bird, reptile, and mammal chromosomes. Proceedings of the National Academy of Sciences. 2021;118 45:e2112494118.
32. Marais G and Galtier N. Sex chromosomes: how XY recombination stops. Current Biology. 2003;13 16:R641-R3.
33. Topaloglu AK, Reimann F, Guclu M, Yalin AS, Kotan LD, Porter KM, et al. TAC3 and TACR3 mutations in familial hypogonadotropic hypogonadism reveal a key role for Neurokinin B in the central control of reproduction. Nature genetics. 2009;41 3:354-8.
34. Zhou Q and Bachtrog D. Sex-specific adaptation drives early sex chromosome evolution in Drosophila. Science. 2012;337 6092:341-5.
35. Hughes JF and Page DC. The biology and evolution of mammalian Y chromosomes. Annual review of genetics. 2015;49:507-27.
36. Miga KH, Koren S, Rhie A, Vollger MR, Gershman A, Bzikadze A, et al. Telomere-to-telomere assembly of a complete human X chromosome. Nature. 2020;585 7823:79-84.
37. Rhie A, Nurk S, Cechova M, Hoyt SJ, Taylor DJ, Altemose N, et al. The complete sequence of a human Y chromosome. Nature. 2023:1-11.
38. Skaletsky H, Kuroda-Kawaguchi T, Minx PJ, Cordum HS, Hillier L, Brown LG, et al. The male-specific region of the human Y chromosome is a mosaic of discrete sequence classes. Nature. 2003;423 6942:825-37.

39. Bellott DW, Skaletsky H, Pyntikova T, Mardis ER, Graves T, Kremitzki C, et al. Convergent evolution of chicken Z and human X chromosomes by expansion and gene acquisition. Nature. 2010;466 7306:612-6.
40. Mueller JL, Skaletsky H, Brown LG, Zaghlul S, Rock S, Graves T, et al. Independent specialization of the human and mouse X chromosomes for the male germ line. Nature genetics. 2013;45 9:1083-7.
41. Ross MT, Grafham DV, Coffey AJ, Scherer S, McLay K, Muzny D, et al. The DNA sequence of the human X chromosome. Nature. 2005;434 7031:325-37.
42. Soh YS, Alföldi J, Pyntikova T, Brown LG, Graves T, Minx PJ, et al. Sequencing the mouse Y chromosome reveals convergent gene acquisition and amplification on both sex chromosomes. Cell. 2014;159 4:800-13.
43. Bachtrog D, Mahajan S, Bracewell R. Massive gene amplification on a recently formed Drosophila Y chromosome. Nature ecology & evolution. 2019;3 11:1587-97.
44. Bhowmick BK, Satta Y and Takahata N. The origin and evolution of human ampliconic gene families and ampliconic structure. Genome research. 2007;17 4:441-50.
45. Yuan L, Pelttari J, Brundell E, Björkroth B, Zhao J, Liu J-G, et al. The synaptonemal complex protein SCP3 can form multistranded, cross-striated fibers in vivo. The Journal of cell biology. 1998;142 2:331-9.
46. Casey AE, Daish TJ and Grutzner F. Identification and characterisation of synaptonemal complex genes in monotremes. Gene. 2015;567 2:146-53.
47. Murat F, Mbengue N, Winge SB, Trefzer T, Leushkin E, Sepp M, et al. The molecular evolution of spermatogenesis across mammals. Nature. 2023;613 7943:308-16.
48. Page SL and Hawley R. The genetics and molecular biology of the synaptonemal complex. Annu Rev Cell Dev Biol. 2004;20:525-58.
49. Tsend-Ayush E, Kortschak RD, Bernard P, Lim SL, Ryan J, Rosenkranz R, et al. Identification of mediator complex 26 (Crsp7) gametologs on platypus X1 and Y5 sex chromosomes: a candidate testis-determining gene in monotremes? Chromosome research. 2012;20:127-38.
50. Blackmon H, Ross L and Bachtrog D. Sex determination, sex chromosomes, and karyotype evolution in insects. Journal of Heredity. 2017;108 1:78-93.
51. Gunski RJ, Cañedo AD, Garnero ADV, Ledesma MA, Coria N, Montalti D and Degrandi T. Multiple sex chromosome system in penguins (Pygoscelis, Spheniscidae). Comparative Cytogenetics. 2017;11 3:541.
52. Miura I, Shams F, Lin S-M, de Bello Cioffi M, Liehr T, Al-Rikabi A, et al. Evolution of a multiple sex-chromosome system by three-sequential translocations among potential sex-chromosomes in the Taiwanese frog Odorrana swinhoana. Cells. 2021;10 3:661.
53. Daish T, Casey A, Grützner F. Platypus chain reaction: directional and ordered meiotic pairing of the multiple sex chromosome chain in Ornithorhynchus anatinus. Reproduction, Fertility and Development. 2009;21 8:976-84. DOI: 10.1071/RD09085.
54. Casey AE, Daish TJ, Barbero JL and Grützner F. Differential cohesin loading marks paired and unpaired regions of platypus sex chromosomes at prophase I. Scientific Reports. 2017;7 1:4217.
55. Manni M, Berkeley MR, Seppey M, Simão FA, Zdobnov E. BUSCO update: novel and streamlined workflows along with broader and deeper phylogenetic coverage for scoring of eukaryotic, prokaryotic, and viral genomes. Molecular biology and evolution. 2021;38 10:4647-54.
56. Huang N and Li H. compleasm: a faster and more accurate reimplementation of BUSCO. Bioinformatics. 2023;39 10:btad595.
57. Li H and Durbin R. Fast and accurate short read alignment with Burrows–Wheeler transform. Bioinformatics. 2009;25 14:1754-60.

58. Danecek P, Bonfield JK, Liddle J, Marshall J, Ohan V, Pollard MO, et al. Twelve years of SAMtools and BCFtools. Gigascience. 2021,10(2):giab008. doi: 10.1093/gigascience/giab008
59. Quinlan AR and Hall I. BEDTools: a flexible suite of utilities for comparing genomic features. Bioinformatics. 2010;26 6:841-2. DOI: 10.1093/bioinformatics/btq033
60. Yang C, Zhou Y, Marcus S, Formenti G, Bergeron LA, Song Z, et al. Evolutionary and biomedical insights from a marmoset diploid genome assembly. Nature. 2021;594 7862:227-33.
61. Ramírez F, Bhardwaj V, Arrigoni L, Lam KC, Grüning BA, Villaveces J, et al. High-resolution TADs reveal DNA sequences underlying genome organization in flies. Nature communications. 2018;9 1:189.
62. Kent WJ. BLAT—the BLAST-like alignment tool. Genome Research. 2002;12 4:656-64. DOI: 10.1101/gr.229202.
63. Bickhart DM, Rosen BD, Koren S, Sayre BL, Hastie AR, Chan S, et al. Single-molecule sequencing and chromatin conformation capture enable de novo reference assembly of the domestic goat genome. Nature genetics. 2017;49 4:643-50.
64. Dudchenko O, Batra SS, Omer AD, Nyquist SK, Hoeger M, Durand NC, et al. De novo assembly of the Aedes aegypti genome using Hi-C yields chromosome-length scaffolds. Science. 2017;356 6333:92-5.
65. Harris RS. Improved pairwise alignment of genomic DNA. The Pennsylvania State University; Ph.D. Thesis, 2007.
66. Kumar S, Stecher G, Suleski M, Hedges S. TimeTree: a resource for timelines, timetrees, and divergence times. Molecular biology and evolution. 2017;34 7:1812-9.
67. Jones BR, Rajaraman A, Tannier E and Chauve C. ANGES: reconstructing ANcestral GEnomeS maps. Bioinformatics. 2012;28 18:2388-90.
68. Wang Y, Tang H, DeBarry JD, Tan X, Li J, Wang X, et al. MCScanX: a toolkit for detection and evolutionary analysis of gene synteny and collinearity. Nucleic acids research. 2012;40 7:e49-e.
69. Tesler G. GRIMM: genome rearrangements web server. Bioinformatics. 2002;18 3:492-3.
70. Benson G. Tandem repeats finder: a program to analyze DNA sequences. Nucleic acids research. 1999;27 2:573-80. DOI: 10.1093/nar/27.2.573.
71. Smit A, Hubley R, Green P. RepeatMasker Open-4.0. http://www.repeatmasker.org.
72. Krzywinski M, Schein J, Birol I, Connors J, Gascoyne R, Horsman D, et al. Circos: an information aesthetic for comparative genomics. Genome research. 2009;19 9:1639-45.
73. Löytynoja AJMsam. Phylogeny-aware alignment with PRANK. Methods Mol Biol. 2014:155-70.
74. Yang Z. PAML 4: phylogenetic analysis by maximum likelihood. Molecular biology and evolution. 2007;24 8:1586-91. https://doi.org/10.1093/molbev/msm088.
75. Stamatakis AJB. RAxML version 8: a tool for phylogenetic analysis and post-analysis of large phylogenies. Bioinformatics. 2014;30 9:1312-3.
76. Sawyer S. Statistical tests for detecting gene conversion. Mol Biol Evol 1989;65:526-38 doi:10.1093/oxfordjournals.molbev.a040567.
77. Lopez-Delisle L, Rabbani L, Wolff J, Bhardwaj V, Backofen R, Grüning B, et al. pyGenomeTracks: reproducible plots for multivariate genomic datasets. Bioinformatics. 2021;37 3:422-3.
78. Martin HC, Batty EM, Hussin J, Westall P, Daish T, Kolomyjec S, et al. Insights into platypus population structure and history from whole-genome sequencing. Molecular Biology and Evolution. 2018;35 5:1238-52.
79. Link V, Aguilar-Gómez D, Ramírez-Suástegui C, Hurst LD, Cortez D. Male mutation bias is the main force shaping chromosomal substitution rates in monotreme mammals. Genome biology and evolution. 2017;9 9:2198-210.

80. Makova KD, Pickett BD, Harris RS, Hartley GA, Cechova M, Pal K, et al. The complete sequence and comparative analysis of ape sex chromosomes. Nature. 2024:1-11.
81. Ramírez F, Ryan DP, Grüning B, Bhardwaj V, Kilpert F, Richter AS, et al. deepTools2: a next generation web server for deep-sequencing data analysis. Nucleic acids research. 2016; https://doi.org/10.1093/nar/gkw257.
82. Bray NL, Pimentel H, Melsted P and Pachter L. Near-optimal probabilistic RNA-seq quantification. Nature biotechnology. 2016;34 5:525-7. DOI: 10.1038/nbt.3519
83. Love MI, Huber W and Anders S. Moderated estimation of fold change and dispersion for RNA-seq data with DESeq2. Genome biology. 2014;15 12:1-21. DOI: 10.1186/s13059-014-0550-8.
84. Yanai I, Benjamin H, Shmoish M, Chalifa-Caspi V, Shklar M, Ophir R, et al. Genome-wide midrange transcription profiles reveal expression level relationships in human tissue specification. Bioinformatics 2005;21 5:650-9.
85. Zhou Y, Jin J, Li X, Gedman G, Pelan S, Rhie A, et al. Supporting data for "Chromosome-level echidna genome illuminates evolution of multiple-sex-chromosome system in monotremes" GigaScience Database. 2024. https://doi.org/10.5524/102609

Figure1

Click here to access/download;Figure;Fig1.pdf

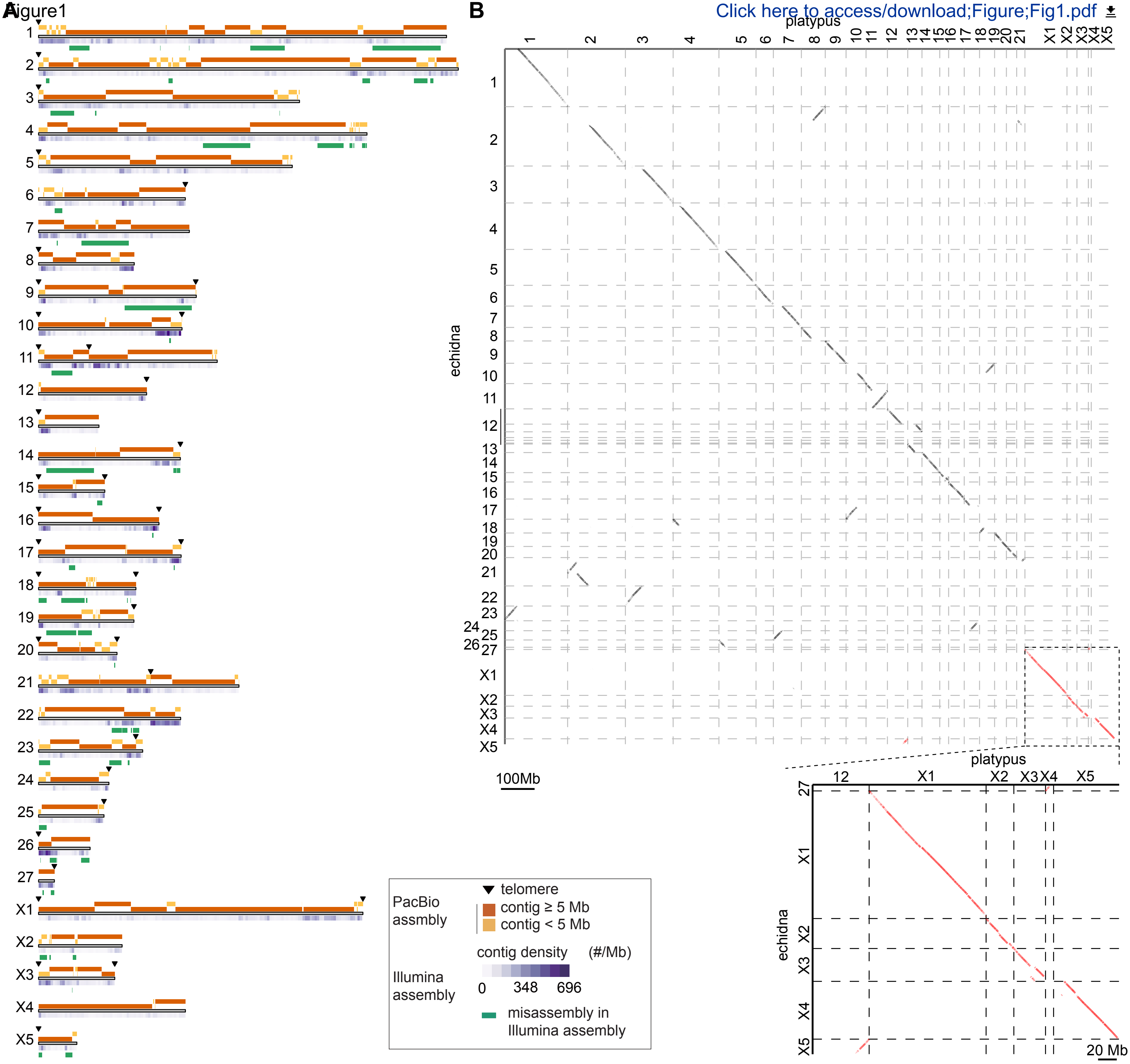

Figure4

Click here to access/download;Figure;Fig4.pdf

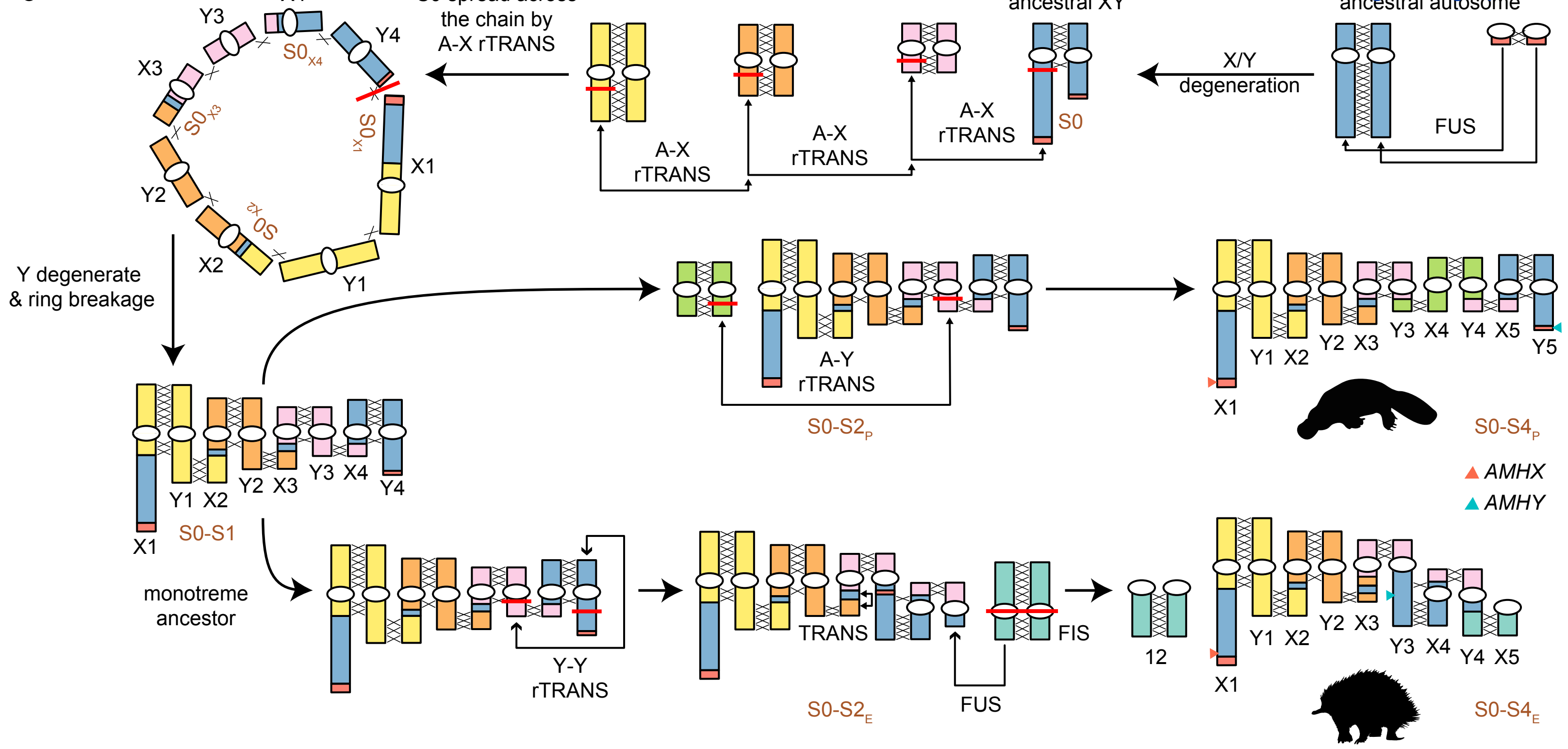

Figure5

Click here to access/download;Figure;Fig5.pdf

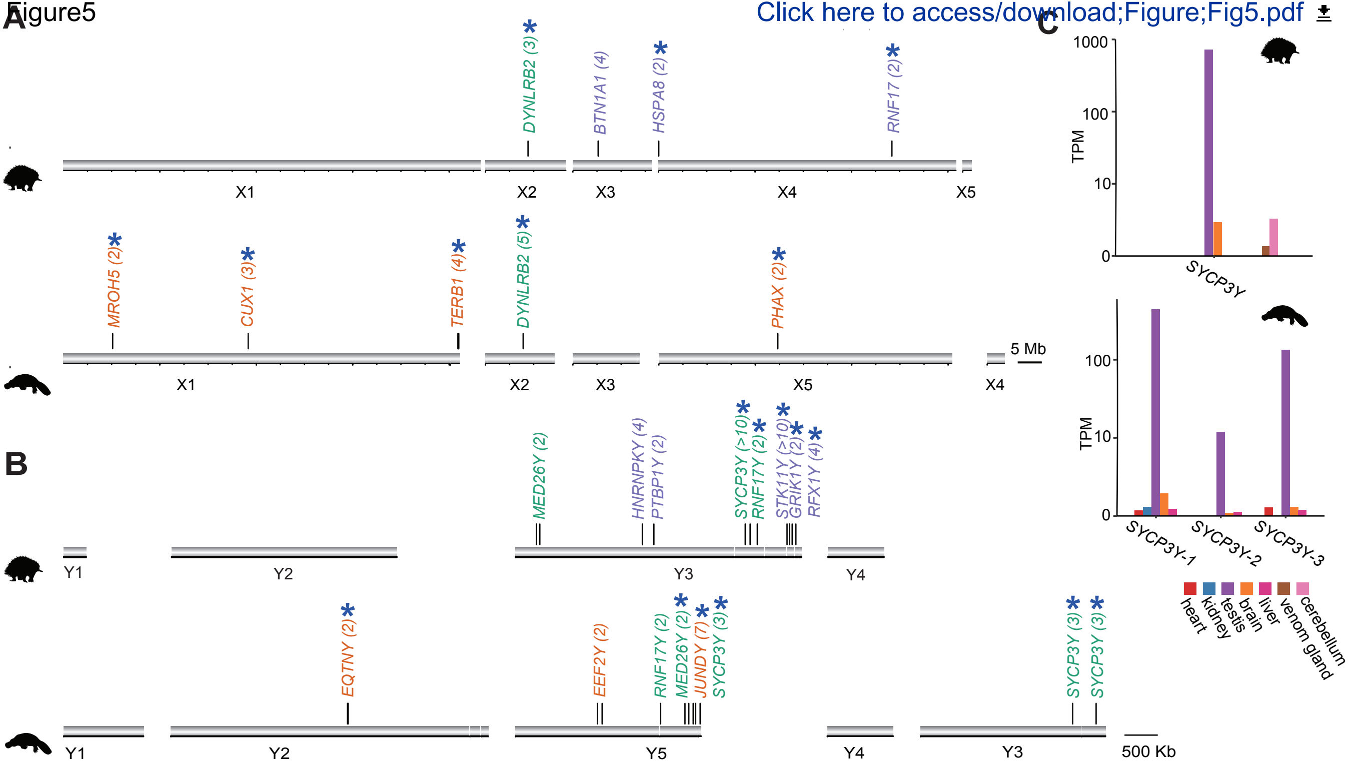

Figure3

Click here to access/download;Figure;Fig3.pdf

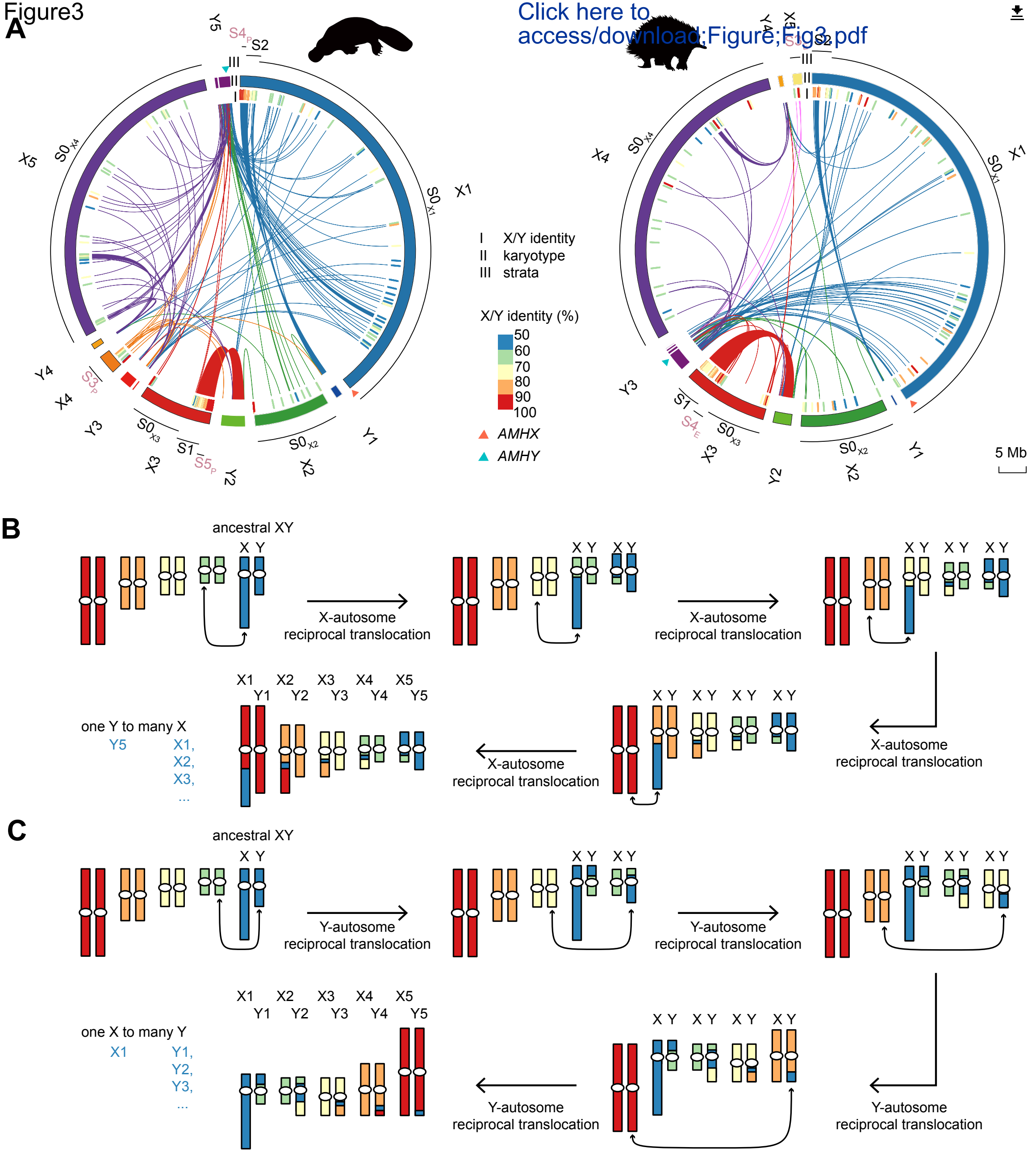

Figure2 Click here to access/download;Figure;Fig2.pdf

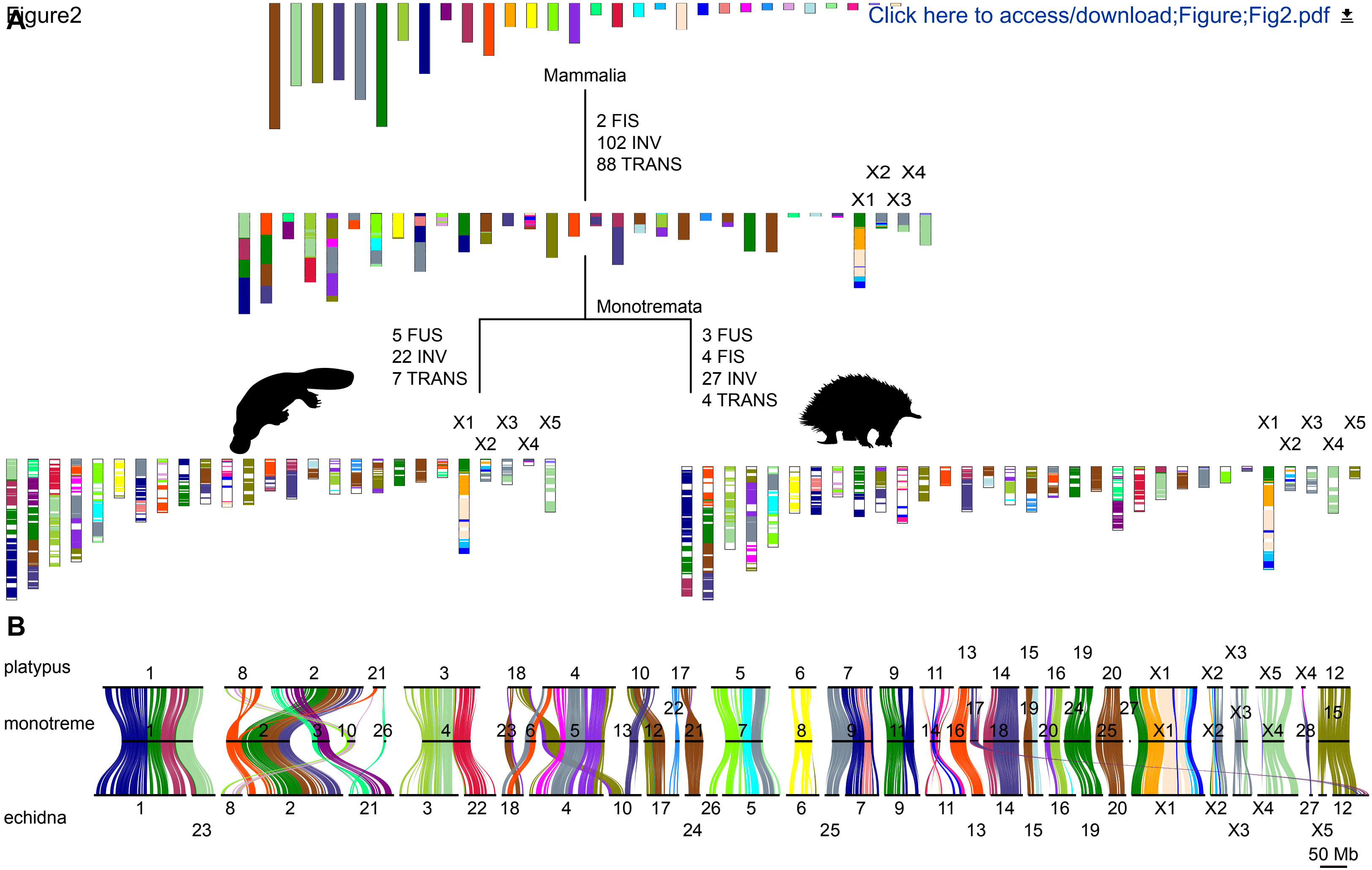

Click here to access/download
Supplementary Material
Supplemental_Text_Figures.final.241014.docx

Click here to access/download
Supplementary Material
Supplemental_Tables.final.240820.v1.xlsx

Response letter

Click here to access/download
Supplementary Material
Response_letter.pdf

 Click here to access/download;Personal Cover;cover_letter.docx

Dear Hongfang Zhang,

Thanks for handling our manuscript. We have revised the manuscript according to the comments from the reviewers. We also organized a response letter to address all referees' comments point-by-point. We hope that you will find this revision can be accepted for publication in Gigascience.

Sincerely,

Guojie Zhang, Ph.D.
Professor
Centre for Evolutionary & Organismal Biology, Zhejiang University School of Medicine
Hangzhou, China
E-mail: guojiezhang@zju.edu.cn
