# Supplementary material for: Chromosome-level echidna genome illuminates evolution of multiple sex chromosome system in monotremes
Source: Gigascience. 2025 Jan 9;14:giae112. doi: 10.1093/gigascience/giae112 (PMC11710854; doi:10.1093/gigascience/giae112)
Supplement: giae112_Response_to_Reviewer_Comments_Original_Submission [file giae112_response_to_reviewer_comments_original_submission.pdf]

Author's Response To Reviewer Comments

Please see the responese in the attached "Response_letter.pdf"
