# Supplementary material for: Chromosome-level echidna genome illuminates evolution of multiple sex chromosome system in monotremes
Source: Gigascience. 2025 Jan 9;14:giae112. doi: 10.1093/gigascience/giae112 (PMC11710854; doi:10.1093/gigascience/giae112)
Supplement: giae112_Reviewer_2_Report_Original_Submission — Dmitry A Filatov --10/4/2024 [file giae112_reviewer_2_report_original_submission.pdf]

Reviewer Report

Title: Chromosome-level echidna genome illuminates evolution of multiple-sex-chromosome system in monotremes

Version: Original Submission **Date:** 10/4/2024

Reviewer name: Dmitry A Filatov

Reviewer Comments to Author:

Overall, this is a nice paper reporting a chromosome-level assembly of echidna genome and comparing with platypus and other mammals to reconstruct the evolution of peculiar system with multiple sex chromosomes in monotremes. I do not have major criticisms, but quite a few minor ones regarding to lack of clarity in the text.

Introduction and discussion are relatively brief and could do a better job with placing the results of this paper in wider context in the field of sex chromosome evolution.

The paper makes comparisons of the genomes of echidna and platypus but never clearly states how divergent platypus and echidna are. What is the average nucleotide divergence between these species at synonymous sites in coding regions (which is the standard measure of neutral sequence divergence)? This needs to be clearly stated somewhere, preferably early in the results or in the introduction.

line 103: "new PacBio-based assembly includes 27 autosomes, five X and four Y" PacBio assembly produces contigs and scaffolds rather than chromosomes. Chromosomes and sex chromosomes are inferred with additional analyses. At this stage in the paper it is not clear how the authors decided which of their scaffolds correspond to which chromosomes and which of them are sex chromosomes. A few words about this have to be added just before the quoted sentence on line 103.

lines 107-108: "five X (X-Div) and four Y chromosomes (Y-Div)" Would be helpful to clarify here why "Div" is used to refer to sex chromosomes

line 114: "Most of the nine chromosomes have over 98% completeness" Need to clarify here how the "98% completeness" was estimated.

Line 115: "98% completeness except Y3 and X5" Please add % completeness for Y3 and X5

Line 120: "two were fragmented and three were missing in the previous assembly. Therefore, this new echidna sex chromosome dataset allows us to provide insight into the evolution of the echidna sex chromosome system." Surprising conclusion about insight into evolution. I do not question the value of the new assembly, but the way text written here is odd - it is not immediately clear how the slight improvement in the assembly ("two genes fragmented and three missing in the previous assembly") helps to infer how sex chromosomes evolved. Evolution can be inferred in comparisons with something (e.g. platypus genome) and the text here needs to mention such comparisons to make it clear how this new genome assembly helps to study evolution.

Line 123: "66 large putative structural variants" These differences may be real genomic polymorphisms, or artefacts. This raises the question about the echidna accessions used for the older and the new assemblies - were these genomes sequenced for different individuals samples in different (possibly quite remote?) locations? Would be helpful to clarify this in the text of the results.

line 152: drop "high frequency". The difference between 63 and 52 chromosomes is not that large. There are many species (e.g. in plants) where chromosome number changes, fusions and fissions occur much more frequently.
Line 225: "In many species, sex chromosome evolution is characterized by recombination suppression. " This is incorrect or incomplete recombination suppression in the heterogametic sex is present in SDR of ALL, rather than many sex chromosomes. It is the EXPANSION of recombination suppression that is observed in many species. Better change this sentence to something like characterised by step-wise or gradual expansion of recombination suppression.
Line 231: " there was no significant difference" between what and what? I presume this sentence means there was no difference in dS between the S0 strata on X1-X4 chromosomes, but this is not clear from this sentence please rephrase.
Line 232: change "previous" to "previously"
Line 267: "suggest an alternative... model" an alternative to what?
Figure2A: Please show on the figure which of the inferred ancestral chromosomes (or regions of chromosomes) corresponds to the X chromosome of mammals (e.g. humans, mice and in marsupials).
Figure 3b and 3c: please label the chromosomes on the diagrams with X and Y to make it clear whether translocations occur to the X or to the Y
Suppl Figs S8 and S9. Please write the names of the species as the black shapes are not so clear e.g. what are the two marsupial species and what is the creature to the right from human (a sloth?)?

Methods

Are the methods appropriate to the aims of the study, are they well described, and are necessary controls included? Choose an item.

Conclusions

Are the conclusions adequately supported by the data shown? Choose an item.

Reporting Standards

Does the manuscript adhere to the journal’s guidelines on minimum standards of reporting? Choose an item.

Choose an item.

Statistics

Are you able to assess all statistics in the manuscript, including the appropriateness of statistical tests used? Choose an item.

Quality of Written English

Please indicate the quality of language in the manuscript: Choose an item.

Declaration of Competing Interests

Please complete a declaration of competing interests, considering the following questions:

- Have you in the past five years received reimbursements, fees, funding, or salary from an organisation that may in any way gain or lose financially from the publication of this manuscript, either now or in the future?
- Do you hold any stocks or shares in an organisation that may in any way gain or lose financially from the publication of this manuscript, either now or in the future?
- Do you hold or are you currently applying for any patents relating to the content of the manuscript?
- Have you received reimbursements, fees, funding, or salary from an organization that holds or has applied for patents relating to the content of the manuscript?
- Do you have any other financial competing interests?
- Do you have any non-financial competing interests in relation to this paper?

If you can answer no to all of the above, write 'I declare that I have no competing interests' below. If your reply is yes to any, please give details below.

I declare that I have no competing interests

I agree to the open peer review policy of the journal. I understand that my name will be included on my report to the authors and, if the manuscript is accepted for publication, my named report including any attachments I upload will be posted on the website along with the authors' responses. I agree for my report to be made available under an Open Access Creative Commons CC-BY license (http://creativecommons.org/licenses/by/4.0/). I understand that any comments which I do not wish to be included in my named report can be included as confidential comments to the editors, which will not be published.

Choose an item.

To further support our reviewers, we have joined with Publons, where you can gain additional credit to further highlight your hard work (see: https://publons.com/journal/530/gigascience). On publication of this paper, your review will be automatically added to Publons, you can then choose whether or not to claim your Publons credit. I understand this statement.

Yes Choose an item.
