# Supplementary material for: Chromosome-level echidna genome illuminates evolution of multiple sex chromosome system in monotremes
Source: Gigascience. 2025 Jan 9;14:giae112. doi: 10.1093/gigascience/giae112 (PMC11710854; doi:10.1093/gigascience/giae112)
Supplement: giae112_Reviewer_3_Report_Original_Submission — Vladimir Trifonov --10/10/2024 [file giae112_reviewer_3_report_original_submission.pdf]

**Reviewer Report**

**Title: Chromosome-level echidna genome illuminates evolution of multiple-sex-chromosome system in monotremes**

**Version: Original Submission** **Date:** 10/10/2024

**Reviewer name: Vladimir Trifonov**

**Reviewer Comments to Author:**

The article reports on the chromosome-level genome assembly of the echidna and reconstructs the details of the monotreme's unique sex chromosome system. The article is very well written, and I suggest only minor revisions to further improve it.

First, I think it is important to emphasize the uniqueness of the high number of reciprocal translocations. These types of rearrangements are very rare in evolution (inversions, fusions, and fissions are more typical). Even in monotremes, we observe these rearrangements only on sex chromosomes, but not on autosomes.

Second, it is important to mention that these reciprocal translocations have only involved the non-recombining parts of the ancestral X chromosomes, and never the recombining parts on the other side. Thus, it may be helpful to distinguish between the "true Ys" - echidna Y3 and platypus Y5 - which carry the sex-determining genes, and other Ys as terms that describe actual autosomal recombining chromosome regions. These regions are not truly involved in sex determination and do not differ in dosage between the sexes.

Perhaps the article would also benefit from a deeper discussion on evolutionary pre-adaptation responsible for the alternate segregation of sex chromosomes in meiosis, which allows for subsequent reciprocal translocations.

Figure 2: I cannot find some species-specific fissions in the figure. For example, echidna chromosomes 22, 23, 25, and 26 appear to result from echidna-specific fission events, but the diagram tracing the lineage from the Monotremata ancestor to the echidna only indicates 25 inversions and 14 translocations.

**Methods**

Are the methods appropriate to the aims of the study, are they well described, and are necessary controls included? Choose an item.

**Conclusions**

Are the conclusions adequately supported by the data shown? Choose an item.

**Reporting Standards**

Does the manuscript adhere to the journal's guidelines on minimum standards of reporting? Choose an item.

Choose an item.

**Statistics**

Are you able to assess all statistics in the manuscript, including the appropriateness of statistical tests used? Choose an item.

**Quality of Written English**

Please indicate the quality of language in the manuscript: Choose an item.

**Declaration of Competing Interests**

Please complete a declaration of competing interests, considering the following questions:

- Have you in the past five years received reimbursements, fees, funding, or salary from an organisation that may in any way gain or lose financially from the publication of this manuscript, either now or in the future?
- Do you hold any stocks or shares in an organisation that may in any way gain or lose financially from the publication of this manuscript, either now or in the future?
- Do you hold or are you currently applying for any patents relating to the content of the manuscript?
- Have you received reimbursements, fees, funding, or salary from an organization that holds or has applied for patents relating to the content of the manuscript?
- Do you have any other financial competing interests?
- Do you have any non-financial competing interests in relation to this paper?

If you can answer no to all of the above, write 'I declare that I have no competing interests' below. If your reply is yes to any, please give details below.

I declare that I have no competing interests

I agree to the open peer review policy of the journal. I understand that my name will be included on my report to the authors and, if the manuscript is accepted for publication, my named report including any attachments I upload will be posted on the website along with the authors' responses. I agree for my report to be made available under an Open Access Creative Commons CC-BY license (http://creativecommons.org/licenses/by/4.0/). I understand that any comments which I do not wish to be included in my named report can be included as confidential comments to the editors, which will not be published.

Choose an item.

To further support our reviewers, we have joined with Publons, where you can gain additional credit to further highlight your hard work (see: https://publons.com/journal/530/gigascience). On publication of this paper, your review will be automatically added to Publons, you can then choose whether or not to claim your Publons credit. I understand this statement.

Yes Choose an item.
